# Eigenvalue based spectral classification

**DOI:** 10.1371/journal.pone.0283413

**Published:** 2023-04-06

**Authors:** Piotr Borkowski, Mieczysław A. Kłopotek, Bartłomiej Starosta, Sławomir T. Wierzchoń, Marcin Sydow

**Affiliations:** Institute of Computer Science, Polish Academy of Sciences, Warsaw, Poland; Ulm University, GERMANY

## Abstract

This paper describes a new method of classification based on spectral analysis. The motivations behind developing the new model were the failures of the classical spectral cluster analysis based on combinatorial and normalized Laplacian for a set of real-world datasets of textual documents. Reasons of the failures are analysed. While the known methods are all based on usage of eigenvectors of graph Laplacians, a new classification method based on eigenvalues of graph Laplacians is proposed and studied.

## 1 Introduction

Graph Spectral Analysis (GSA) is a known technique for clustering of objects whose relations can be best described by a graph linking these objects on the grounds of their similarity [1–3]. This is in particular true for text data, where the similarity of documents can be expressed by the number of common words or in terms of more sophisticated descriptions (e.g. cosine similarity), see e.g. [4]. GSA exploits eigen-decomposition of the so-called graph Laplacians, being a transformation of the similarity matrix.

A known disadvantage of the original GSA is that its output does not comprise a method for assignment of new data items to the existing clusters. In practice, either a clustering from scratch or training of some external classification model is needed. Re-clustering from scratch may be a serious problem for large data collections, while the classification by the external model raises the question: does the subcluster added by the classifier to the original cluster fit the cluster definition? Still another approach may consist in performing GSA for a (bigger) portion of new data and then in an attempt to assign the clusters of the new data to the old clusters. Therefore, several approaches were proposed to handle this issue, like [5–8]. This paper can be seen as a contribution to this type of research. The mentioned approaches concentrate on transforming eigenvectors, while our method relies on eigenvalues only.

The issues with GSA may become more grievant if we expect that the clustering should fit some predefined concepts that is the data comes with (at least partial) labeling. It turns out that the label may be derived based on the textual contents of the data item (we call them *endogenous labels*) or may at least partially represent external knowledge (we call them *exogenous labels*). The question that we investigate in this paper is: Does there exist a GSA based characterization of data set common to endogenous and exogenous labeling as well as to unlabelled data such that new data groups can be correctly assigned to existent data categories (either clusters or classes). This task can be viewed as a specific case of classification. We investigated this issue for several real-world data sets, described in section 2. These datasets were chosen to represent endogenous and exogenous labeling as well as unlabelled data.

The Graph Spectral Analysis was used in the past not only for purposes of cluster analysis (unsupervised learning) but also in classification tasks (supervised learning). GSA was harnessed for such tasks in a number of ways, including:

- “natural classification”—the clusters resulting from spectral clustering are labeled with majority classes from the labeled data set [9]
- “cluster-based classification”—a large number of clusters is generated from the spectral analysis and then a classifier is applied to clusters, trained by majority labels of the clusters [10]
- spectral eigenvector based classification—in the process of spectral clustering the step of clustering by e.g. *k*-means in the space spanned by lowest eigenvalue related eigenvectors is replaced with a classifier trained in that space [11].

**Fig 1:**
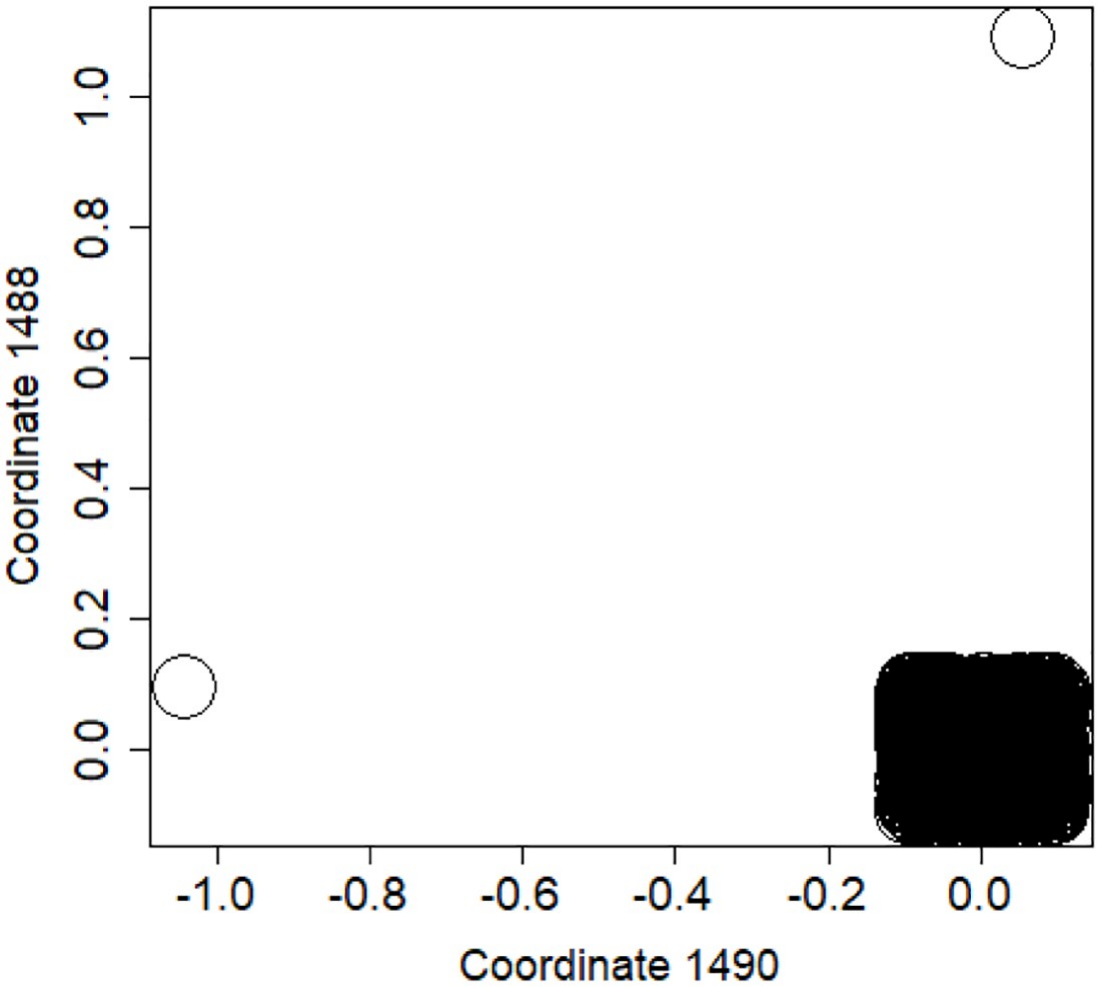
Distribution of objects in the space spanned by the eigenvectors of combinatorial Laplacian corresponding to some of the lowest eigenvalues (no. 1490 and 1488)—TWT.PL dataset: In two corners there are two objects, while the rest is located in the third corner (mass concentration). The positions of datapoints are slightly blurred so that the mass concentration is visible.

While each of these approaches has its own advantages, we have encountered datasets (examples listed in Sect.2) where many of them perform poorly. We investigate in section 4.1 ten different GSA methods and show that reasonable results are obtained by some of them only for datasets with endogenous labeling. It also turns out that none of the GSA methods is superior to the other for each dataset. “Natural classification” and “cluster-based classification” rely on GSA returning clusters with relatively high purity which is not achieved for several investigated sets (see sections 4.2 and 4.3 resp.). “Spectral eigenvector based classification” requires reliability in posing all data points (training set and the test set) in a common space which is a problem for GSA, as visible in section 4.4. In section 5, we show why relying on eigenvectors turned to be so ineffective on the real-world datasets. We found out that in many eigenvectors the mass is concentrated in a few elements. This effect is visible e.g. in Fig 1, where, in the space spanned by eigenvectors with lowest eigenvalues, the vast majority of datapoints concentrates in a single point, while only a few of them reside elsewhere. In section 6 we show that there is an issue with noise in the eigenvectors for lowest eigenvalues even for the easiest datasets (with endogenous labeling). This means that spectral clustering cannot work for this type of data. Same applies to any kind of traditional spectral classification, based on eigenvectors.

These insights led us to an investigation whether or not there exists a different face of GSA that can be used for classification purposes. We turned our attention to the spectrum of eigenvalues and studied their applicability to classification task. The algorithm proposed in this paper allows to classify portions of documents into predefined classes. The algorithm has the following structure (details in section 7):

- Compute the vector of combinatorial or normalized Laplacian eigenvalues of all classes and of the new data set.
- Then make a decision based on some dissimilarity criteria between the class spectra and the new data set spectrum.
- The class is selected for which the difference between these vectors is the lowest.

**Fig 2:**
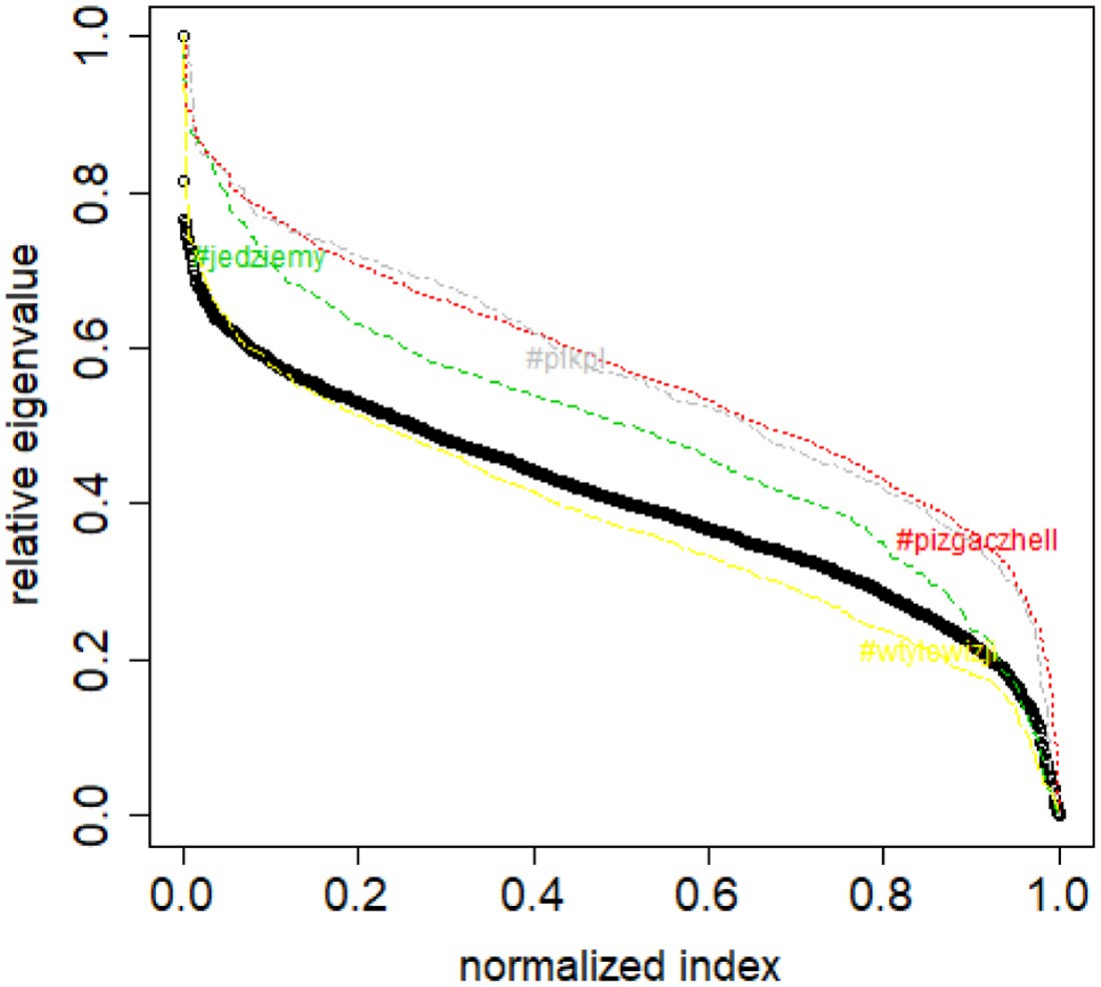
Spectral normalization in the Combinatorial Laplacian Relative Lambda Method method. The TWT.PL dataset.

**Fig 3:**
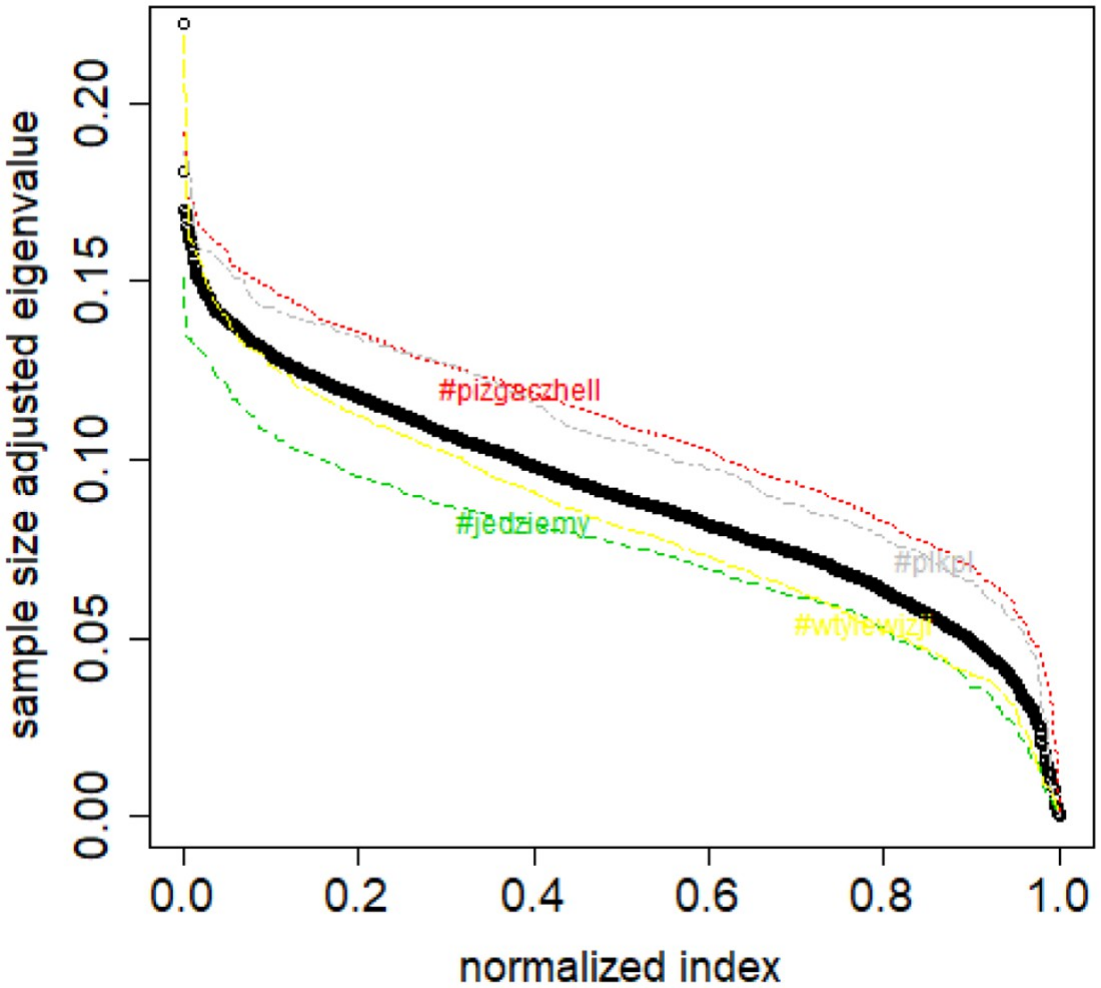
Spectral normalization in the Combinatorial Laplacian Sample Size Adjusted Lambda Method method and Combinatorial Laplacian Sample Size Adjusted Maximum Lambda Method method. The TWT.PL dataset.

**Fig 4:**
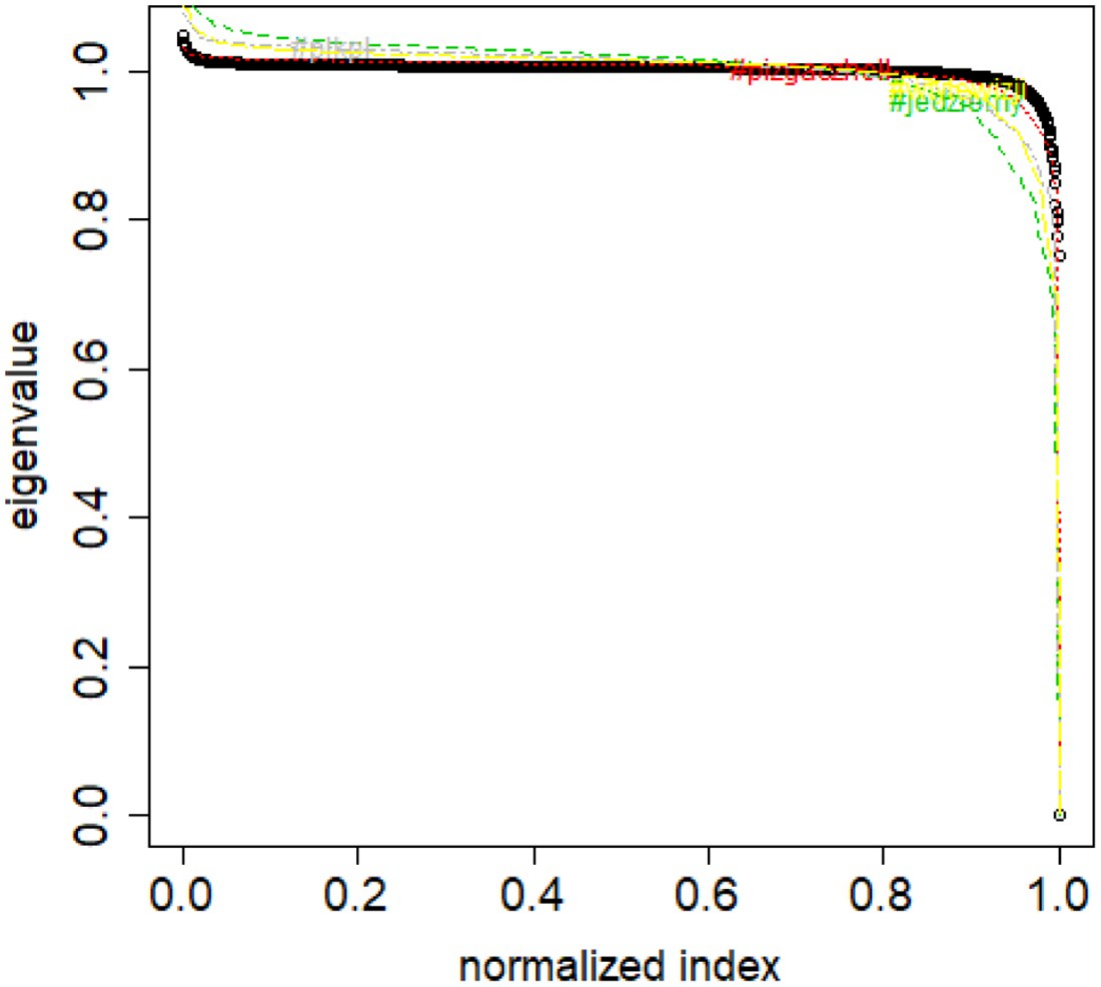
Spectral normalization in the Normalized Laplacian Method method The TWT.PL dataset.

We investigated the following (dis)similarity criteria:

- normalize the spectra by dividing by the largest eigenvalue, then the dissimilarity is equal to an (approximate) integral between the class spectrum and the new data set spectrum (Combinatorial Laplacian Relative Lambda Method, CLRL)); see Fig 2,
- normalize the spectra by dividing by the dataset size (class or new data set), then the dissimilarity is equal to an (approximate) integral between the class spectrum and the new data set spectrum (Combinatorial Laplacian Sample Size Adjusted Lambda Method, CLSSAL); see Fig 3,
- normalize the spectra by dividing by the dataset size (class or new data set), then the dissimilarity is equal to the absolute difference between largest eigenvalues (Combinatorial Laplacian Sample Size Adjusted Maximum Lambda Method, CLMXL); see Fig 3,
- compute not the combinatorial Laplacian but rather the Normalized Laplacian (which has always by definition the largest eigenvalue equal to 1), then the dissimilarity is equal to an (approximate) integral between the class spectrum and the new data set spectrum (Normalized Laplacian Method, NLL); see Fig 4.

The experimental study of the effectiveness of our method is presented in section 8 and the conclusions are described in section 10 after a discussion in section 9. Let us first provide with an overview of application of spectral clustering in classification tasks in section 3.

## 2 The datasets

For purposes of our investigation, we have chosen several real-world datasets from the following domains: tweets, product descriptions for retail enterprises and news headlines. They are characterized by either endogenous or exogenous labeling, with varying number of categories and in two different natural languages (English and Polish) to avoid the situation that the methods are language specific. Also an artificial dataset was added to see the effects of predefined properties on the classification outcome.

We refrained from using standard datasets and used instead real-world examples so that the evaluation is not affected by any “curing” methods.

**Fig 5:**
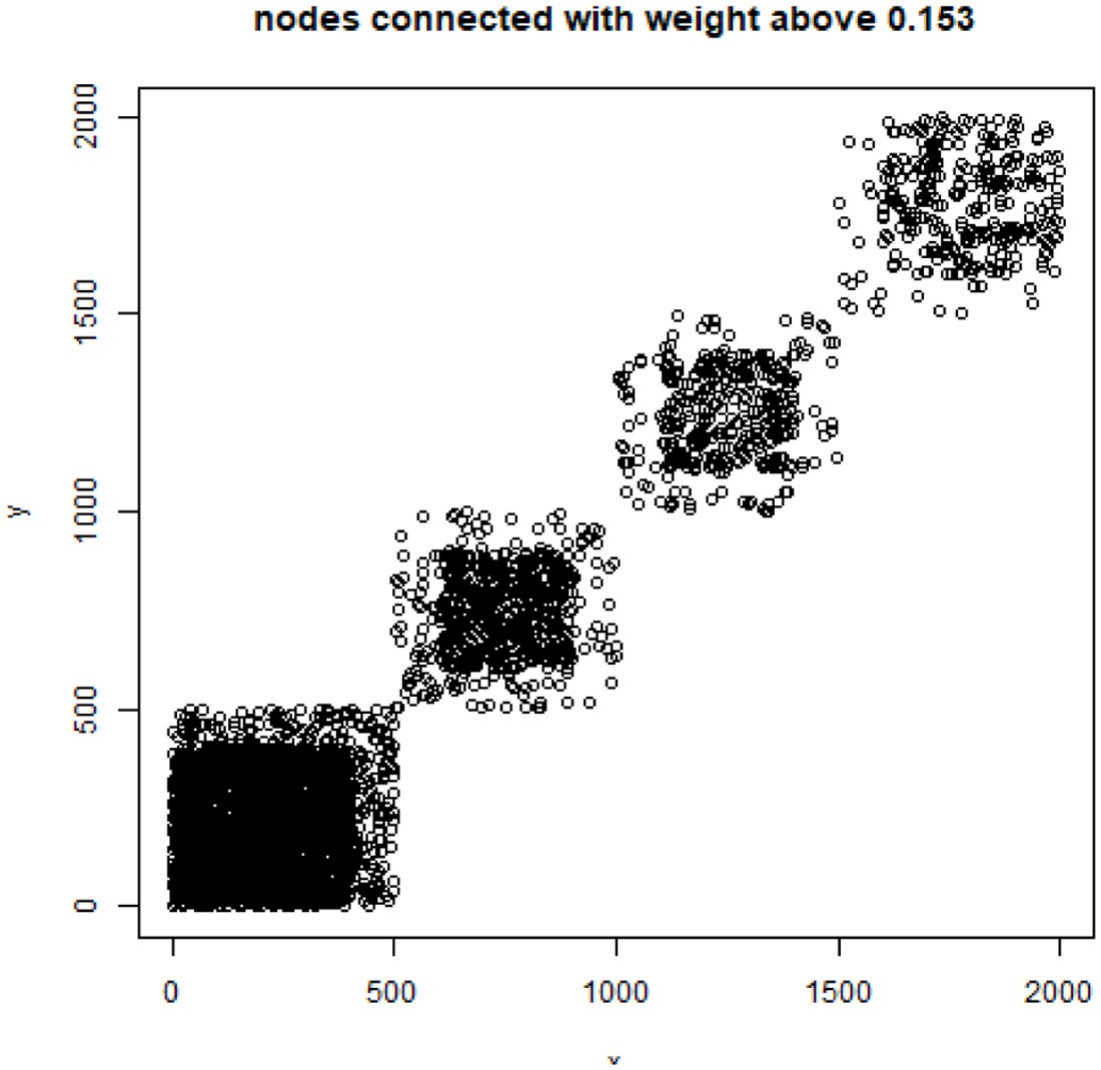
The artificial data set BLK.4_0.2_0.5—adjacency matrix for documents.

We investigated the following datasets:

- TWT.EN—a collection of English language tweets with 1844 records, 5 classes, named #aewdynamite, #demdebate, #puredoctrinesofchrist, #tejran, #trump2020 with minimal cardinality 300 and maximal cardinality 454—choice from manually selected tweet tag list for tweet lengths without tags min. 132 (which implied max. length 270).
- TWT.PL—a collection of Polish language tweets, 1491 records, 4 classes, named #jedziemy, #pizgaczhell, #plkpl, #wtylewizji with minimal cardinality 221 and maximal cardinality 622—choice from manually selected tweet tag list for tweet lengths without tags min. 77 (which implied max. length 274). TWT datasets are available from the authors upon request.
- SEN.EN.ent—a manually labeled publicly available collection of 1000 EN language news headlines, described in [12], divided by the attribute ent into 3 classes named Biden, Sanders, Trump with minimal cardinality 117 and maximal cardinality 755—choice of classes driven by minimum requested cardinality 100.
- SEN.EN.maj—a manually labeled publicly available collection of 564 EN language news headlines, described in [12], divided by the attribute maj into 2 classes named neg, pos with minimal cardinality 178 and maximal cardinality 386—choice of classes driven by minimum requested cardinality 100.
- SEN.PL.ent—a manually labeled publicly available collection of 877 PL language news headlines, described in [12], divided by the attribute ent into 5 classes named Duda, Morawiecki, Polska, Putin, Trump with minimal cardinality 102 and maximal cardinality 371—choice of classes driven by minimum requested cardinality 100.
- SEN.PL.maj—a manually labeled publicly available collection of 585 PL language news headlines, described in [12], divided by the attribute maj into 2 classes named neg, pos with minimal cardinality 260 and maximal cardinality 325—choice of classes driven by minimum requested cardinality 50.
- ANO.8—a manually labeled collection of product descriptions (anonymized) from a chain shop, divided into 8 (anonymized) COICOP classes, 364 records, 5 classes were considered, named 0, 1, 4, 6, 8 with minimal cardinality 31 and maximal cardinality 110—choice driven by the minimum class cardinality set to 30.
- ANO.26—a manually labeled collection of product descriptions (anonymized), from a chain shop, divided into 26 (anonymized) COICOP classes, 95 records, 11 classes, named 0, 1, 11, 13, 14, 15, 22, 23, 3, 7, 9 with minimal cardinality 31 and maximal cardinality 177—choice driven by the minimum class cardinaliy set to 30.
- ANO.44—a manually labeled collection of product descriptions (anonymized) from still another chain shop, divided into 44 (anonymized) COICOP classes, 146 records, 4 classes, named 0, 11, 20, 22 with minimal cardinality 33 and maximal cardinality 40—choice driven by the minimum class cardinaliy set to 30.
- ANO.94—a manually labeled collection of product descriptions (anonymized) from still another chain shop, divided into 94 (anonymized) COICOP classes. 1881 records, 3 classes, named 54, 62, 63 with minimal cardinality 537 and maximal cardinality 686—choice driven by the minimum class cardinaliy set to 500. All ANO.* datasets were manually labelled by humans who did not have any external knowledge of product properties and therefore most probably represent endogenous labeling. These are proprietary datasets.
- BLK.4_0.2_0.5—a synthetic set of 2000 “product descriptions” divided into 4 classes; the dataset was generated by a random generator aiming at identification of the underlying mechanisms for success/failure of our method; An overview of the adjacency matrix is visible in Fig 5.. File name contains three parameters of the generation process: groupCount(here 4), overlap (0.2), minprob (0.5). GroupCount tells how many intrinsic clusters are generated. The groups are generated as follows: A “dictionary” is created and each cluster is assigned a separate portion of the dictionary. Overlap means what percentage of cluster dictionaries shall overlap with the other clusters. Minprob is the minimum probability that a word from the dictionary occurs in the “document” belonging to a cluster. Besides the dictionaries also “noise” words are added to each document from any position of the dictionary.

Classes with less than 15 elements were generally removed from each data set, which affected the ANO.* datasets. Besides, other restrictions on cardinality were imposed as mentioned above.

The ANO.* datasets represent endogenously labelled data. The SEN.* datasets represent exogenously labelled data. The BLK dataset is an artificial dataset that may be considered as endogenously labelled. The TWT.* datasets may be considered as a mixture of endogenously and exogenously labelled data.

## 3 Previous work

Classification of text documents is a hot topic in applied Machine Learning, see e.g. [13] for a review of various attempts to this problem. Of particular interest is classification of short documents. It is challenging due to data sparseness when applying classic text representations (such as “bag of words”), and rather small number of words occurring in such documents. To classify short texts a fusion of Machine Learning, Natural Language Processing, and Deep Learning methods is used to help create meaningful and relevant categories from small pieces of text data, see e.g. [14] for details.

### 3.1 Spectral-based approaches

The conventional usage of spectral analysis is to apply it to clustering based on relaxed versions of ratio cut (RCut) and normalized cut (NCut) graph clustering methods. These are realized by applying *k*-means algorithm to the rows of the matrix, the columns of which are eigenvectors associated with the *k* lowest eigenvalues of the corresponding graph Laplacian.

More precisely, let *S* be a similarity matrix between pairs of items (e.g. documents). It induces a graph whose nodes correspond to the items. A(n unnormalised) or combinatorial Laplacian *L* corresponding to this matrix is defined as

$$\begin{array}{c} L=D-S, \end{array}$$ (1)

where *D* is the diagonal matrix with $d_{jj}=\sum_{k=1}^{n}s_{jk}$ for each *j* ∈ [*n*]. A normalized Laplacian L of the graph represented by *S* is defined as

$$\begin{array}{c} L=D^{-1/2}LD^{-1/2}=I-D^{-1/2}SD^{-1/2} \end{array}$$ (2)

The RCut criterion corresponds to finding the partition matrix $P_{RCut}\in R^{n\times k}$ that minimizes the formula trace(*H*^*T*^*LH*) over the set of all partition matrices $H\in R^{n\times k}$. Such formulated problem is NP-hard. That is why we relax it by assuming that *H* is a column orthogonal matrix. In this case the solution is obvious: the columns of *P*_*RCut*_ are eigenvectors of *L* corresponding to *k* smallest eigenvalues of *L*. Similarly, the columns of matrix *P*_*NCut*_, representing NCut criterion, are eigenvectors of L corresponding to *k* smallest eigenvalues of L. For an explanation and further details see e.g. [3] or [15].

The following modifications are applicable: (1) use the top eigenvalue eigenvectors of the matrix *D*^−1/2^*SD*^−1/2^ instead of the lowest ones [11, 16], (2) normalize to unit length the rows of the aforementioned eigenvector sub-matrix prior to *k*-means clustering, (3) use more than *k* eigenvectors to cluster into *k* clusters, [17], (4) instead of clustering, a supervised learning method can be applied, usually on a subset of the rows of the aforementioned sub-matrix and then apply the learned classifier to the remaining rows.

The spectral clustering (unsupervised learning) methods have been accommodated to the task of classification (both supervised and semi-supervised learning) in several ways.

Kamvar, Klein and Manning proposed in [16] a simple but very efficient solution: Given a spectral representation of the data they classify them using any reasonable classifier trained on the labeled points. They state that in the supervised case, their approach achieves high accuracy on the categorization of thousands of documents given only a few dozen labeled training documents for the 20 Newsgroups data set.

Dudek [9] presents a very common idea that spectral clustering can be used as a “classification” method in that “natural clusters” are detected via spectral cluster analysis and then labels may be attached relating the cluster contents to some labelled data set.

Suganthi and Manimekalai [11] propose to adapt conventional spectral cluster analysis procedures to the task of classification by replacing the part of proper clustering (via *k*-means) with a classification method relying on the *k* largest eigenvectors.

Mahmood and Mian [10] suggest to cluster data first and then to apply classification techniques to the clusters.

Fowlkes et al. [18] propose a classification method associated with spectral clustering in that first an approximation of the spectral kernel is computed, generating initial clusters (for a subset of data points), and later on the kernel is expanded to other data points allowing for clustering of a large graph. This method was enhanced to cover even larger graphs by Pourkamali [19].

Karami et al. [20] construct ensembles of clustering and classification algorithms to create new such algorithms better fitting data at hand.

Owhadi et al. [21] target creation of one-class-classifiers exploiting spectral clustering methods due to better class boundary characteristics, and at the same time claim to keep scalability property.

Li and Hao [22] proposed a semi-supervised sentiment classification method. Another use of semi-supervised spectral clustering was proposed by Liu, Shen, and Pan in [23].

### 3.2 Non-spectral-based approaches

Aside from spectral methods there are many works on non-spectral methods with interesting connections between clustering and classification concerning short texts (as in our study: datasets considered in our experiments concern tweets, news headlines, short product descriptions, etc.).

To improve the performance, the solutions partially focus on the problem of efficient text representation in such tasks. Spectral methods could be considered as such, but many other exist. Thus, similarly to spectral-based methods, the non-spectral frameworks for short-text clustering or classification usually consist of 2 modules, where the first one concerns learning the text representation (e.g. word embedding or language model) and the second one is some clustering or classification module built on top of the output of the first one.

One of the most popular strategy is to use neural networks to learn embeddings of the words (sentences, paragraphs, etc.) in low-dimensional spaces e.g. ([24, 25], etc. and all the numerous follow-up works, e.g. [26]). While such methods are powerful, usually the word order is (at least partially) lost what can be alleviated by applying also recurrent neural networks to take the word order into account (e.g. [27]).

Concerning unsupervised techniques, [28] or [29] apply unsupervised auto-encoders to improve text representation. Also the technique of contrastive learning can be used to make the text representation better separated in the representation space (e.g. [30]) The techniques for reducing dimensionality can be used to generate additional labels to be then used by a convolutional network to improve text representation (e.g. [31]).

Recently, a great progress in pre-trained language models (e.g. [32, 33], etc.) makes it possible to achieve much more powerful contextual text representations that results in potential advances in short-text classification or clustering (e.g. [30], etc.).

Another interesting technique concerning intersections of classification and clustering of short texts is presented in [34] where a classifier is trained with cluster labels to improve the previous clustering.

### 3.3 Remarks on kernel clustering

As stressed by [35], both spectral and kernel clustering methods use or can be explained by usage of eigen-decomposition of the similarity matrix and the clustering in the space spanned by appropriately selected eigen vectors. Hence, a unified view of both spectral clustering and kernel methods as a clustering of an embedding instead of (Ratio/N)-cut approximations or feature space mappings was elaborated in the past. While they were considered in principle applicable for clustering, one can consider applying them to classification along the paths taken for spectral clustering. Dhillon et al. [36] elaborated conditions when spectral clustering, kernel clustering and graph cut clustering would converge to the same result. We [37] made the differences between various spectral and kernel clustering methods explicit in that we explored the differences in the way how the graphs are embedded.

Let us recall two of the types of Laplacian Kernels: the Regularized Laplacian Kernel *K*_*RLK*_(*t*) = (*I* + *tL*)^−1^ and the Modified Personalized PageRank Based Kernel *K*_*MPPRK*_(*α*) = (*D* − *αS*)^−1^, 0 < *α* < 1, described by e.g. Avrachenkov [38]. A closer look at the definitions of the two kernels. *K*_*RLK*_(*t*) and *K*_*MPPRK*_(*α*), reveals that both of them can be considered as approximated inverse of *L*. *K*_*MPPRK*_(*α*) does so with *α* → 1, *K*_*RLK*_(*t*) with *t* → ∞. *K*_*RLK*_(*t*) divided by *t* approximates it when *t* → ∞. This means that their eigenvectors and inverted eigenvalues approximate those of *L*. Nonetheless the coordinates in the embeddings are distinct from those of *L* as they are multiplied by inverse square roots of eigenvalues.

## 4 The problem with spectral clustering

In this paper we focus on two fundamental variants of spectral clustering, namely clustering based on combinatorial Laplacian and clustering based on the normalized Laplacian. It is well known that the first variant corresponds to the minimization of the RCut, while the second—to the minimization of the NCut criterion, see e.g. [15] for an explanation.

### 4.1 A comparison of various spectral clustering methods applicable to classification

As mentioned in the introduction, the literature proposes three basic approaches to classification based on GSA, i.e.

- “natural classification”
- “cluster-based classification”
- spectral eigenvector based classification

We have investigated their effectiveness for 10 different versions of GSA:

1. csc.b—Combinatorial spectral clustering
2. csc.ur—Combinatorial spectral clustering with normalizing the data point rows
3. csc.urdp—Combinatorial spectral clustering with normalizing the data point rows and an additional dimension
4. csc.ka—Spectral clustering method proposed by Kamvar et al. [16]
5. csc.kadp—Spectral clustering method proposed by Kamvar et al. [16] with additional dimension
6. nsc.b—Normalized spectral clustering
7. nsc.ur—Normalized spectral clustering with unit length rows
8. nsc.urdp—Normalized spectral clustering with unit length rows and one additional dimension used
9. nsc.ursvd—Normalized spectral clustering operating on data cleaned up via SVD with unit length rows
10. nsc.ursvddp—Normalized spectral clustering operating on data cleaned up via SVD with unit length rows and one additional dimension

Whenever we speak about “additional dimension”, we mean that when using *k*-means clustering within the spectral clustering procedure, we use not *k* but *k* + 1 eigenvectors associated with lowest eigenvalues (in case of Kamvar et al. method—the highest eigenvalues are considered). The idea of using additional dimension was born from our experiments which showed clustering and classification improvements for some data sets (see e.g. nsc.urdp and nsc.ursvddp rows compared to nsc.ur and nsc.ursvd in Tables 1 and 2). However, adding more dimensions introduced noise to the tasks.

**Table 1:** Error percentage for natural classification. Column names: datasets, row names: GSC methods considered.

Meth./set	ANO.8	ANO.26	ANO.44	ANO.94
csc.b	44.51	78.1	21.23	63.37
csc.ur	21.98	64.47	20.55	63.37
csc.urdp	23.08	66.03	1.37	63.37
csc.ka	21.98	64.47	20.55	63.37
csc.kadp	23.08	66.48	1.37	63.37
nsc.b	36.81	36.76	1.37	49.71
nsc.ur	7.14	31.06	0.68	63.32
nsc.urdp	6.87	30.84	0	63.37
nsc.ursvd	3.3	30.84	0.68	35.19
nsc.ursvddp	3.02	26.93	0	5.42

**Table 2:** F1 score for natural classification. Column names: datasets, row names: GSC methods considered.

Meth./set	ANO.8	ANO.26	ANO.44	ANO.94
csc.b	29.57	8.64	70.03	18.14
csc.ur	69.87	22.51	71.52	18.15
csc.urdp	68.55	18.53	98.55	18.15
csc.ka	69.87	22.51	71.52	18.15
csc.kadp	68.55	20.16	98.55	18.15
nsc.b	51.28	46.3	98.61	38.8
nsc.ur	92.37	51.31	99.31	18.27
nsc.urdp	92.64	52.09	100	18.15
nsc.ursvd	96.13	52.01	99.31	55.52
nsc.ursvddp	96.89	65.04	100	94.84

In subsequent subsections we show the error rates obtained for each of these basic classification methods.

### 4.2 Investigation of natural classification

In “natural classification” the clusters resulting from spectral clustering are labeled with majority classes from the labeled data set [9].

The success of the natural classification relies on the capability of creating clusters fitting the prior labelling. Therefore, in Tables 1, 3 and 4 we present our investigation on the agreement of clusters with the prior labeling in terms of error rate and in Tables 2, 5 and 6—F1 measure.

**Table 3:** Error percentage for natural classification. Column names: datasets, row names: GSC methods considered.

Meth./set	SEN.EN.maj	SEN.EN.ent	SEN.PL.maj	SEN.PL.ent
csc.b	31.56	24.32	0	57.31
csc.ur	31.56	24.32	0	57.42
csc.urdp	31.56	24.42	44.44	57.54
csc.ka	31.56	24.32	44.44	57.42
csc.kadp	31.56	24.42	44.44	57.54
nsc.b	31.56	24.42	0	49.83
nsc.ur	31.56	24.42	0	48.33
nsc.urdp	31.56	21.92	34.19	47.18
nsc.ursvd	31.56	24.42	34.19	48.33
nsc.ursvddp	31.56	21.92	33.5	47.18

**Table 4:** Error percentage for natural classification. Column names: datasets, row names: GSC methods considered.

Meth./set	TWT.EN	TWT.PL	BLK.4.0.2.0.5
csc.b	75.16	58.08	34.3
csc.ur	61.5	58.15	24.5
csc.urdp	67.35	58.01	24.5
csc.ka	61.5	58.15	24.5
csc.kadp	67.14	57.88	24.5
nsc.b	51.25	58.28	24.8
nsc.ur	49.78	58.28	24.95
nsc.urdp	50.38	58.28	24.95
nsc.ursvd	49.78	58.28	24.95
nsc.ursvddp	50.38	58.28	24.95

**Table 5:** F1 score for natural classification. Column names: datasets, row names: GSC methods considered.

Meth./set	SEN.EN.maj	SEN.EN.ent	SEN.PL.maj	SEN.PL.ent
csc.b	40.63	29.23	35.72	12.72
csc.ur	40.63	29.23	35.72	12.32
csc.urdp	40.63	29.2	35.72	11.92
csc.ka	40.63	29.23	35.72	12.32
csc.kadp	40.63	29.2	35.72	11.92
nsc.b	40.63	28.7	35.72	23.19
nsc.ur	40.63	28.7	35.72	24.41
nsc.urdp	40.63	48.6	63.23	33.45
nsc.ursvd	40.63	28.7	64.12	24.41
nsc.ursvddp	40.63	48.6	64.37	33.45

**Table 6:** F1 score for natural classification. Column names: datasets, row names: GSC methods considered.

Meth./set	TWT.EN	TWT.PL	BLK.4.0.2.0.5
csc.b	8.37	15.29	57.64
csc.ur	28.02	15.06	74.91
csc.urdp	17.94	15.4	74.91
csc.ka	28.02	15.06	74.91
csc.kadp	18.16	16.84	74.91
nsc.b	44	14.72	75.59
nsc.ur	45.2	14.72	75.44
nsc.urdp	44.77	14.72	75.42
nsc.ursvd	45.2	14.72	75.44
nsc.ursvddp	44.77	14.72	75.42

As one could have expected, the natural clustering does not work in all cases. Good results were obtained for dataset ANO.8, ANO.44, SEN.PL.maj. The nsc.ur GSA clustering method shows the best performance in most cases, and normalized spectral clustering is superior to combinatorial one. However, the other data sets need apparently other approach to classification task. One of the reasons may be that natural clustering works predominantly for endogenous labeling.

### 4.3 Investigation of cluster-based classification

In “cluster-based classification”, a large number of clusters is generated from the spectral analysis and then a classifier is applied to clusters, trained by majority labels of the clusters [10].

The success of cluster-based classification depends on the possibility of creating a large number of clusters that are as pure as possible with respect to the prior clustering.

The tables presenting the results of our experiments can be found Section B in S1 Appendix.

In Tables 20–41 in S1 Appendix we show the errors (impurity) of clusters obtained when the number of original clusters was increased 2, 4 and 8 times.

As visible from Tables 22, 24 and 26 in S1 Appendix, cluster-based classification has a chance to improve classification accuracy significantly. But the increase of the number of clusters may have also disadvantageous effects on classification results, as visible in some cases in Table 32 in S1 Appendix.

So, for the dataset types at hand, the cluster-based classification does not achieve the expected improvements in classification potential.

### 4.4 Investigation of spectral eigenvector based classification

In spectral eigenvector based classification, in the process of spectral clustering the step of clustering by e.g. *k*-means in the space spanned by lowest eigenvalue related eigenvectors is replaced with a classifier trained in that space [11].

In our investigation, shown in Tables 7–12 we used the well-known decision tree algorithm implemented in R in rpart package. The approach was as follows: The data were divided randomly in training part (2/3) and test pat (1/3). The clustering procedure for the training data was modified in that instead of *k*-means application, decision tree algorithm was applied to construct a classifier. The clustering procedure for the test data was modified in that instead of *k*-means application, the previously trained decision tree classifier was applied to the data assigning class labels. We report the error rate of this algorithm.

**Table 7:** Error percentage for eigenvector based classification. Column names: datasets, row names: GSC methods considered.

Meth./set	ANO.8	ANO.26	ANO.44	ANO.94
csc.b	38.46	67.06	23.91	62.24
csc.ur	45.19	59.22	17.39	0
csc.urdp	23.08	67.45	19.57	0
csc.ka	43.27	71.37	0	0
csc.kadp	9.62	69.41	32.61	0
nsc.b	21.15	54.12	17.39	36.55
nsc.ur	43.27	61.96	21.74	0
nsc.urdp	70.19	57.65	26.09	0
nsc.ursvd	8.65	53.33	17.39	20.52
nsc.ursvddp	11.54	61.57	26.09	36.55

**Table 8:** F1 measure for eigenvector based classification. Column names: datasets, row names: GSC methods considered.

Meth./set	ANO.8	ANO.26	ANO.44	ANO.94
csc.b	53.86	13.2	66.03	20.03
csc.ur	47.19	20.27	69.44	17.97
csc.urdp	68.69	15.01	67.61	17.97
csc.ka	55.14	13.98	66.67	17.97
csc.kadp	90.99	16.01	64.97	17.97
nsc.b	71.6	29.58	69.14	51.02
nsc.ur	50.59	18.92	64.82	17.97
nsc.urdp	10.56	21.84	60.83	17.97
nsc.ursvd	NA	NA	NA	NA
nsc.ursvddp	72.56	20.74	60.83	50.5

**Table 9:** Error percentage for eigenvector based classification. Column names: datasets, row names: GSC methods considered.

Meth./set	SEN.EN.maj	SEN.EN.ent	SEN.PL.maj	SEN.PL.ent
csc.b	0	0	41.08	55.4
csc.ur	0	0	0	0
csc.urdp	0	0	0	0
csc.ka	0	0	0	0
csc.kadp	0	0	0	0
nsc.b	32.39	24.82	0	0
nsc.ur	0	24.82	0	0
nsc.urdp	0	24.82	0	0
nsc.ursvd	0	19.34	40.54	55.4
nsc.ursvddp	0	24.82	40.54	50

**Table 10:** F1 measure for eigenvector based classification. Column names: datasets, row names: GSC methods considered.

Meth./set	SEN.EN.maj	SEN.EN.ent	SEN.PL.maj	SEN.PL.ent
csc.b	40.34	28.61	37.08	12.34
csc.ur	40.34	28.61	37.08	12.34
csc.urdp	40.34	28.61	37.08	12.34
csc.ka	40.34	28.61	37.08	12.34
csc.kadp	40.34	28.61	37.08	12.34
nsc.b	40.34	28.61	37.08	12.34
nsc.ur	40.34	28.61	37.08	12.34
nsc.urdp	40.34	28.61	37.08	12.34
nsc.ursvd	NA	NA	NA	NA
nsc.ursvddp	40.34	28.61	47.61	23.86

**Table 11:** Error percentage for eigenvector based classification. Column names: datasets, row names: GSC methods considered.

Meth./set	TWT.EN	TWT.PL	BLK.4.0.2.0.5
csc.b	71.86	54.78	24.25
csc.ur	72.62	54.78	23.37
csc.urdp	72.81	55.24	23.37
csc.ka	72.62	54.78	31.11
csc.kadp	72.81	56.18	31.11
nsc.b	49.24	56.41	36.91
nsc.ur	49.24	55.48	17.93
nsc.urdp	43.73	56.41	12.48
nsc.ursvd	49.24	55.48	23.02
nsc.ursvddp	43.73	56.41	12.48

**Table 12:** F1 measure for eigenvector based classification. Column names: datasets, row names: GSC methods considered.

Meth./set	TWT.EN	TWT.PL	BLK.4.0.2.0.5
csc.b	11.84	19.12	75.63
csc.ur	10.51	19.12	75.28
csc.urdp	10.16	18.36	75.28
csc.ka	10.51	18.2	65.52
csc.kadp	10.16	17.22	65.52
nsc.b	36.08	15.18	53.1
nsc.ur	37	24.53	82.33
nsc.urdp	41.12	15.18	87.62
nsc.ursvd	37	24.53	75.28
nsc.ursvddp	41.12	15.18	87.62

The spectral eigenvector based classification worked well for the majority of datasets, though it performed poorly for ANO.8, ANO.26, TWT.EN and BLK.4_0.2_0.5.

## 5 A probable reason for failures of the investigated spectral clustering methods

The observed problems in the behaviour of various types of spectral clustering methods on various types of datasets, as illustrated in section 4, prompted us for a more thorough investigation of the reasons for these failures. In this section we investigate some general issues, and in section 6 we perform a detailed case-study of one aspect, the noise in the eigenvectors.

We observed the following behaviour: whatever number of classes we considered, the vast majority of clusters produced by the spectral clustering contained only a couple of objects, while the rest was concentrated in one or two large clusters. This effect may be visualized when looking at datapoints drawn in the coordinate system spanned by two eigenvectors related to low eigenvalues, as visible e.g. in. Fig 1 for the TWT.PL dataset. The data is concentrated in one corner, while only a few datapoints reside elsewhere. Same effects can be observed in other datasets.

**Fig 6:**
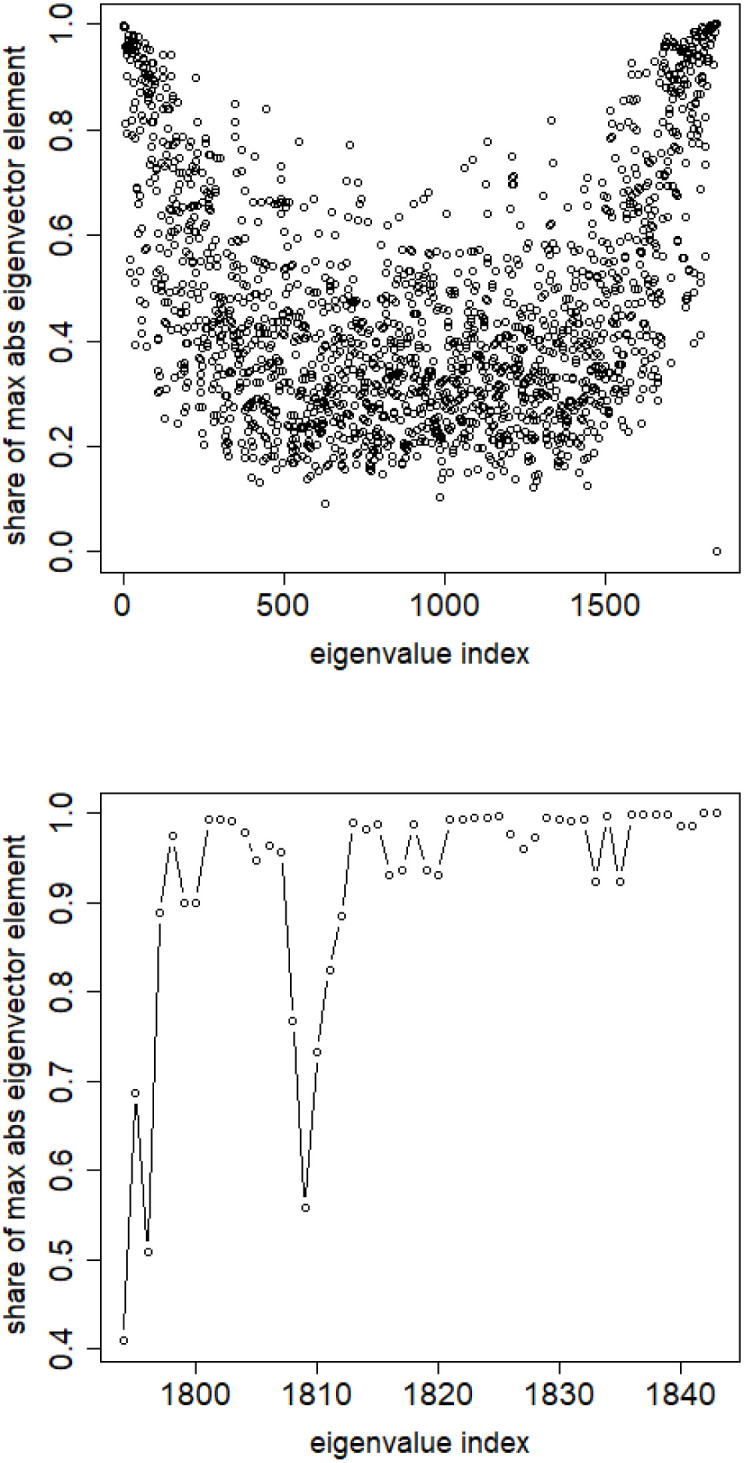
Mass of the element with the largest mass in the eigenvector. Eigenvectors are ordered by decreasing eigenvalue. Top figure: the entire spectrum. Bottom figure: only the 50 eigenvectors corresponding to 50 lowest eigenvalues. English Twitter data TWT.EN.

This may be explained as follows: Let **v** be an eigenvector. The quantity ${∥v∥}^{2}=\sum_{i=1}^{n}v_{i}^{2}$ will be called its mass. Similarly, $v_{i}^{2}$ will be called the mass of its *i*-th element. Obviously as all eigenvectors are normalized, their masses are equal to 1. Fig 6 shows the distribution of the “heaviest” elements, $v_{j}^{*}={\text{max}}_{i}v_{ij}^{2}$, in all eigenvectors **v**_*j*_, *j* ∈ [*n*] of an exemplary Laplacian. The horizontal axis (index) indexes the eigenvectors of a Laplacian ordered according to decreased eigenvalues.

The vertical axis shows the maximal squared element value of the respective eigenvector. From this figure it follows that generally over 20% of the mass of eigenvectors concentrates in their “heaviest” elements (the top picture). It is much worse in the eigenvectors with the lowest 50 eigenvalue (the bottom picture) as there nearly everywhere over 80% of the mass is concentrated in the single largest eigenvector element. Special attention paid to the eigenvectors related top lowest eigenvalues is justified by the fact that the spectral clustering (via e.g. *k*-means) is run in the space spanned by them.

**Fig 7:**
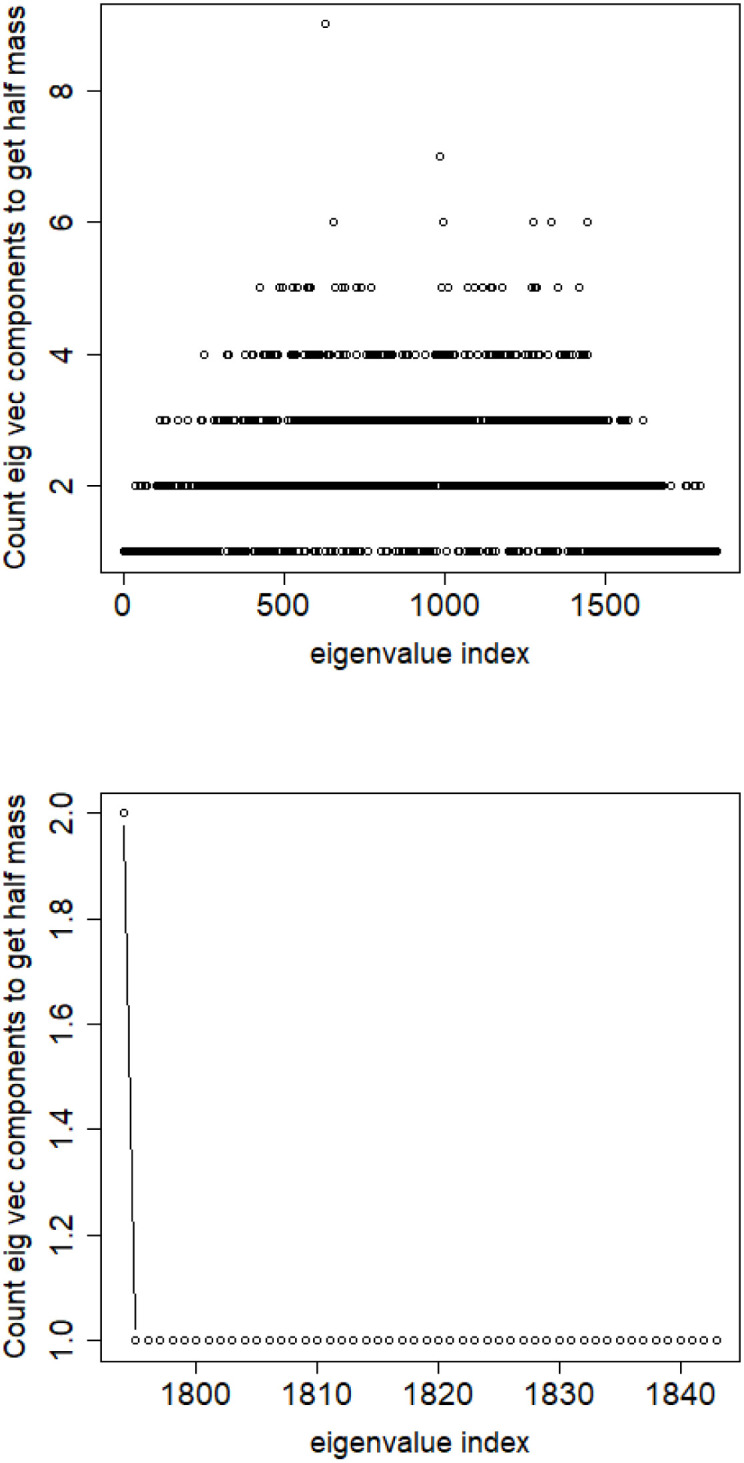
Number of highest mass elements of eigenvectors constituting half mass of the eigenvector. English Twitter data TWT.EN. Top: all eigenvectors. Bottom: 50 eigenvectors with the lowest eigenvalue.

Fig 7 shows the problem from a slightly different perspective. It shows how many largest mass elements of an eigenvector are necessary to account for half of the mass of the eigenvector. Consider an eigenvector **v**. Let *o*(*i*, **v**) be an invertible function assigning each *i* ∈ [1, *n*] the position *j* such that *v*_*o*(*i*, **v**)_ ≥ *v*_*o*(*i* + 1, **v**)_. For each vector **v** we seek $\text{arg}{\text{min}}_{j}\sum_{i=1}^{j}v_{o(i,v)}\geq 0.5$.

In vast majority of the cases only 5 elements are necessary—out of over 3,000. Among the eigenvectors with the lowest 50 eigenvalue only one element is enough.

**Fig 8:**
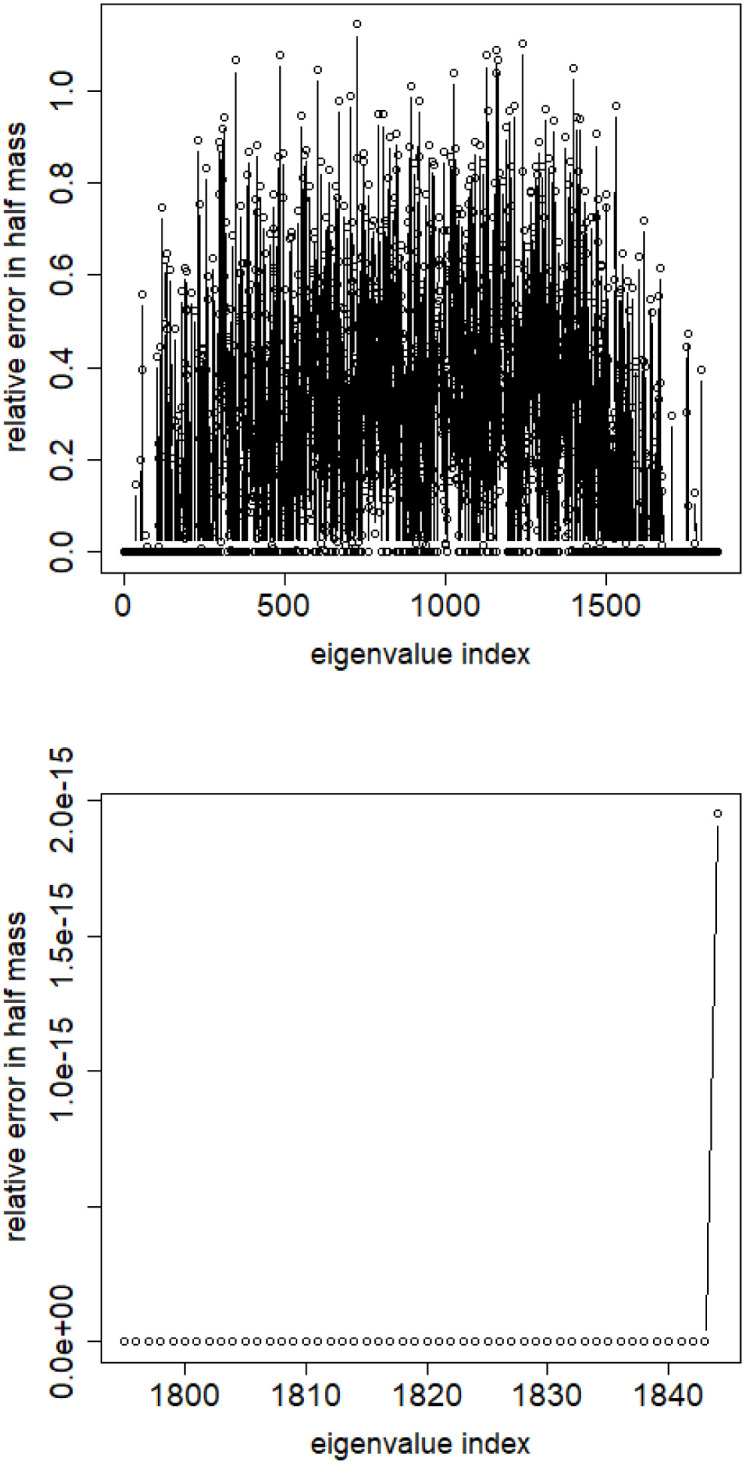
Relative error among the elements of eigenvector constituting its halfmass (standard error divided by the mean). English Twitter data TWT.EN. Top: all eigenvectors. Bottom: 50 eigenvectors with the lowest eigenvalue.

Fig 8 presents the ratio of square rooted variance of masses to the mean mass in the elements belonging to the previously mentioned half-mass, *r*_*h*_, that is if for an eigenvector **v** we have $t=\text{arg}{\text{min}}_{j}\sum_{i=1}^{j}v_{o(i,v)}\geq 0.5$, then $m_{h}=\frac{1}{t}\sum_{i=1}^{t}v_{o(i,v)}$, $v_{h}=\frac{1}{t}\sum_{i=1}^{t}{\left(v_{o(i,v)}-m_{h}\right)}^{2}$, and the $r_{h}=\frac{\sqrt{v_{h}}}{m_{h}}$. The small “relative error” indicates that the elements in the half-mass do not differ very much.

We can conclude from this insight that for the datasets under consideration the spectral clustering based on combinatorial Laplacian is unable to provide with meaningful clusters.

At the initial stage of our investigation, we have worked hard to get around the problem of mass concentration of low eigenvalue eigenvectors by applying diverse similarity measures. We computed cosine similarity for term-frequency-based document vectors, term-frequency-inverse-document-frequency document vectors, we centralized these vectors or not, we used dot products instead of cosine similarities. Nothing helped around the problem, only shifted it and while some improvements were observed in one dataset, a worsening of the problem was visible in another set. Normalized Laplacians were affected negatively by the vector centralizing. So finally we decided to use the plain cosine similarity and sought solutions elsewhere, as reported here. Similar problems were reported, by the way, by [39].

## 6 Another problems with spectral clustering

A number of empirical studies indicate that the normalized graph cut, exploiting eigenvectors of the normalized Laplacian, often leads to better (compared to RCut) clustering results. The same behaviour is observed when analysing datasets described in section 2. Unfortunately, even this variant is not robust against imbalanced datasets. We illustrate this on two examples. Another serious problem is that of unequal distribution of the eigenvalues, see e.g. [40] for a deeper treatment of this problem.

Consider first the dataset ANO.8. It is composed of 9 groups of cardinalities listed in Table 13. Clustering original dataset we obtain extremely poor results. Thus we delete the groups 3, 4, 6, 8, i.e., “small” groups with cardinality not greater than 20. As a result we obtain a subset consisting of 364 items. Further, let us replace original data matrix *X* by the SVD approximation $X\approx U_{r}\Sigma_{r}V_{r}^{T}$ with *r* = 250. By running standard spectral clustering (based on Normalized Laplacian) on such “denoised” data we obtain clustering accuracy = 0.9670. The confusion matrix is shown in Table 14.

**Table 13:** Original cardinalities of groups in the dataset ANO.8.

group:	1	2	3	4	5	6	7	8	9
Card.:	65	93	18	15	31	2	110	1	65

**Table 14:** Cluster membership confusion matrix for ANO.8 after removing small groups. Rows represent TRUTH, columns represent PREDICTION.

	group				
	1	2	5	7	9
1	64	1	0	0	0
2	0	90	1	1	1
5	0	0	30	1	0
7	1	0	1	107	1
9	0	1	2	1	61

The quality of clustering is affected by low values in the degree matrix. In this particular example we encounter for data rows *j* = 299, 363 the *deg*(*j*)≤10^−6^, and $1/\sqrt{deg(299)}=1.7482e+07$, $1/\sqrt{deg(363)}=5.1873e+07$. Setting both values in the degree matrix to zero, the quality of clustering is decreased. However, the removal of respective rows and columns of the similarity matrix improves the quality.

In this example small groups are just acting as disturbing noise. For instance adding 3rd group of cardinality 18 we obtain confusion matrix shown in Table 15. Surprisingly, the last group divides now into subgroup of cardinality 17 and the “core” of cardinality 45.

**Table 15:** Cluster membership confusion matrix for ANO.8 after removing small groups, but retaining the 3rd group. Rows represent TRUTH, columns represent PREDICTION.

	group					
	1	2	**3**	5	7	9
1	64	1	**0**	0	0	0
2	0	90	**0**	0	1	2
**3**	**0**	**16**	**0**	**0**	**0**	**2**
5	0	0	**0**	30	1	0
7	1	0	**2**	0	106	1
9	0	1	**17**	1	1	45

Consider now a larger set ANO.26. Like ANO.8 this set consists of 26 groups of various sizes. Nine groups with the numbers and cardinalities shown in Table 16 were selected for the analysis.

**Table 16:** Original cardinalities of selected groups in the dataset ANO.26.

group:	1	2	4	12	14	15	16	23	24
Card:	65	93	106	110	113	177	44	56	65

Then, after replacing original subset by the SVD approximation with *r* = 450 we obtain a partition with the accuracy 0.7944. The confusion matrix is shown in Table 17.

**Table 17:** Cluster membership confusion matrix for ANO.26 after removing small groups. Rows represent TRUTH, columns represent PREDICTION.

	group								
	1	2	4	12	14	15	16	23	24
1	64	1	0	0	0	0	0	0	0
2	0	90	0	1	0	0	0	0	2
4	0	0	101	0	0	0	1	4	0
12	2	0	0	101	1	3	0	2	1
14	0	0	0	0	107	5	0	0	1
15	27	2	2	49	10	78	1	6	2
16	0	7	0	3	3	1	25	0	5
23	2	0	2	2	1	0	5	42	0
24	0	1	0	0	2	2	11	0	49

This time the largest group #15 consisting of 177 elements splits into two larger subgroups of cardinalities 49 and 784 and into seven other subgroups of cardinalities 27, 2, 2, 10, 1, 6, 2. Interestingly, when replacing original subset with *r* = 450 columns chosen according to the naive procedure described above, we obtain slightly better partition with the accuracy 0.0.79692.

Surprisingly, deleting group #15 only slightly improves accuracy. Its present value is 0.8077 and the confusion matrix is given in Table 18.

**Table 18:** Cluster membership confusion matrix for ANO.26 after removing small groups and the group #15. Rows represent TRUTH, columns represent PREDICTION.

	group							
	1	2	4	12	14	16	23	24
1	14	1	50	0	0	0	0	0
2	0	90	0	1	0	0	0	2
4	13	1	88	0	0	1	3	0
12	0	0	0	103	2	1	2	1
14	0	0	0	1	111	0	0	1
16	0	7	0	2	3	27	0	5
23	0	1	4	1	1	5	43	0
24	0	1	0	2	2	11	0	49

To summarize our findings, we see that there is a problem with relying on eigenvectors when performing the classification with GSA, and therefore we suggest the exploration of a different dimension of GSA, that is the eigenvalues.

## 7 Eigenvalue-based approach to classification

As already described in the Introduction, our algorithm aims to classify portions of documents into predefined classes. Our approach relies on finding a common characterization of samples of documents belonging to the same class. It turned out to be an eigenvalue spectrum with the modifications we explain below. In this Section, we shall show that:

- the eigenvalue spectra of a document class Laplacian and of its subsets are close to one another upon appropriate scaling of eigenvalue index and eigenvalues themselves.
- Small perturbations of similarity matrices cause only small perturbatuions of the entire spectrum, in particular decreasing the similarity decreases the spectrum.
- These properties can be used to create an algorithm classifying homogeneous groups of documents into known classes of documents.

### 7.1 Motivation

**Fig 9:**
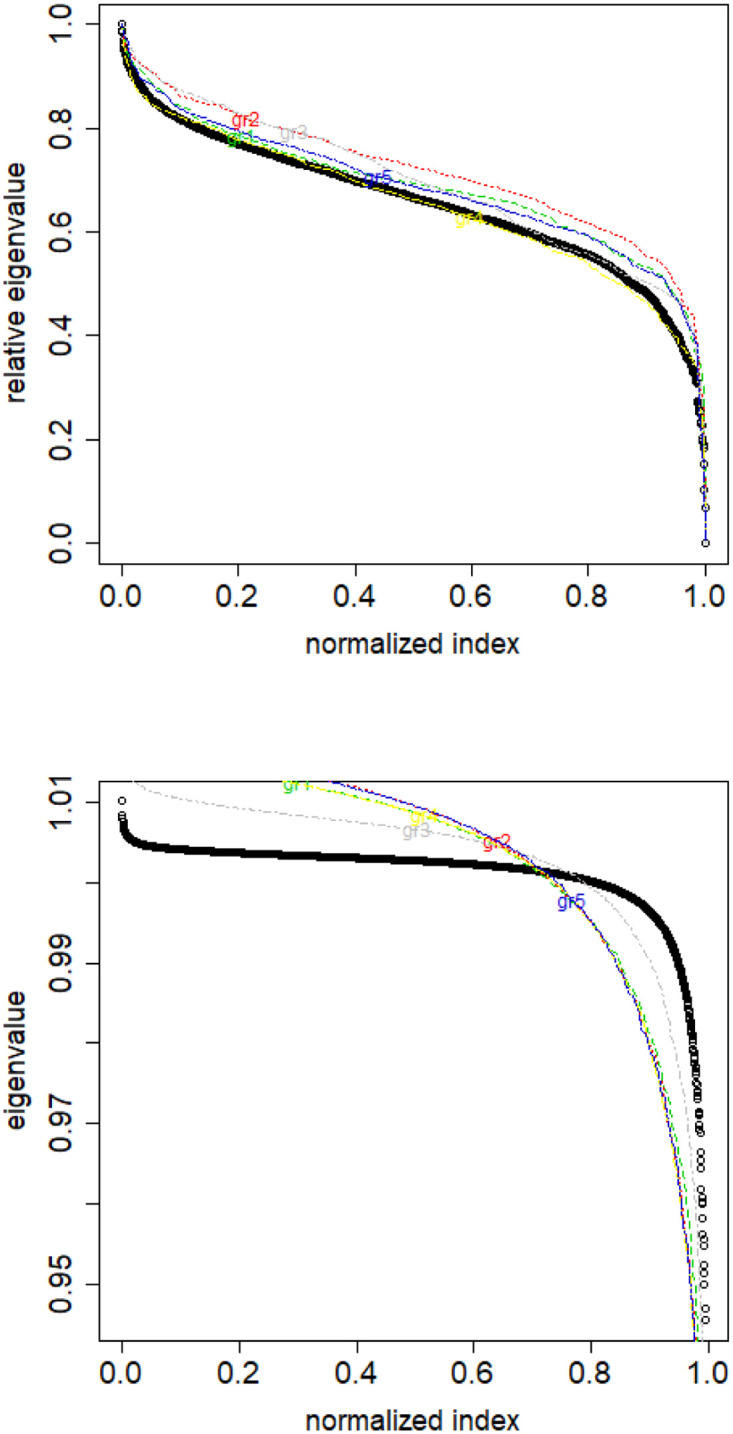
Eigenvalue distributions for the entire dataset (the black dots) and for the classes (lines with different colors) for combinatorial (top) and normalized (bottom) graph Laplacian. English Twitter data TWT.EN. On the bottom, ten lowest eigenvalues were omitted for better readability.

An illustration of the method’s motivation is presented in Fig 9. Appropriate scaling of the eigenvalue indices allows to reveal the differences between the spectra of different classes of documents for both combinatorial and normalized Laplacian.

The starting point for this investigation is that for a class of objects *X* the similarity distribution *S*_*X*_ over *X* × *X* is hypothesized to be independent of sampling. That is, if we draw two equally sized samples $X_{1}^{\prime },X_{2}^{\prime }$ uniformly and randomly from *X*, then the similarity distribution *S*′ over $X_{1}^{\prime }\times X_{2}^{\prime }$ is (in some sense) the same (under appropriate ordering in samples) as *S*′′ over $X_{1}^{\prime \prime }\times X_{2}^{\prime \prime }$ when we draw two equally sized samples $X_{1}^{\prime \prime },X_{2}^{\prime \prime }$ uniformly and randomly from *X*, given that we sort each sample according to some unique criterion. This is easy to imagine if we compute similarities based on words in documents, whereby there exists a word distribution characteristic for documents in the domain *X*. A number of topic detection models, like Probabilistic Latent Semantic Allocation (PLSA) [41] or Latent Dirichlet Allocation (LDA) [42] make the assumption that the vocabulary related to a topic, or discourse domain, is coming from a topic-specific probability distribution. In our case, when dealing with short texts, it is unlikely that more than one topic is present in the same document. Hence documents from the same domain/topic are likely to have the same word distribution.

Subsequently we will demonstrate how this assumption can be transferred into the domain of Laplacian eigenvalue spectra.

**Fig 10:**
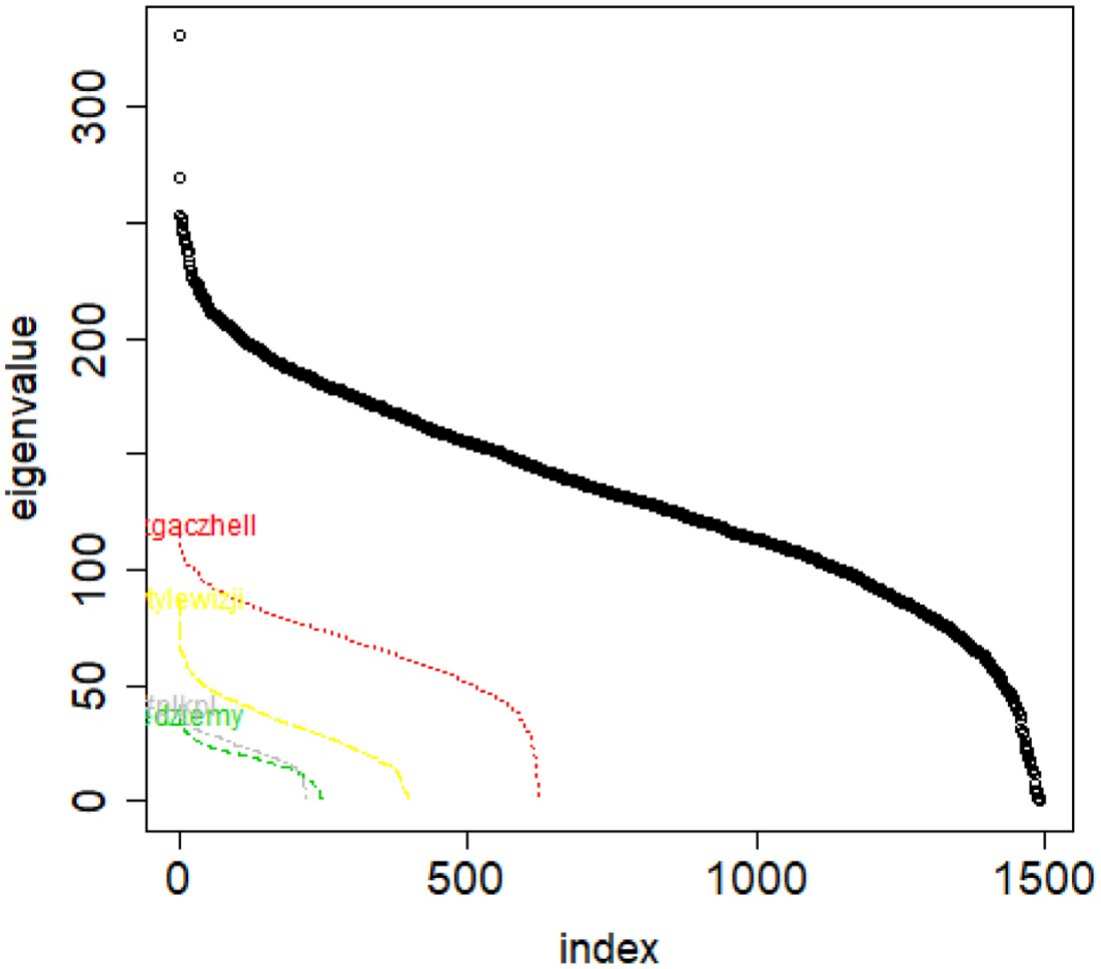
Combinatorial Laplacian of the entire TWT.PL data set (thick line) and of each of the classes.

**Fig 11:**
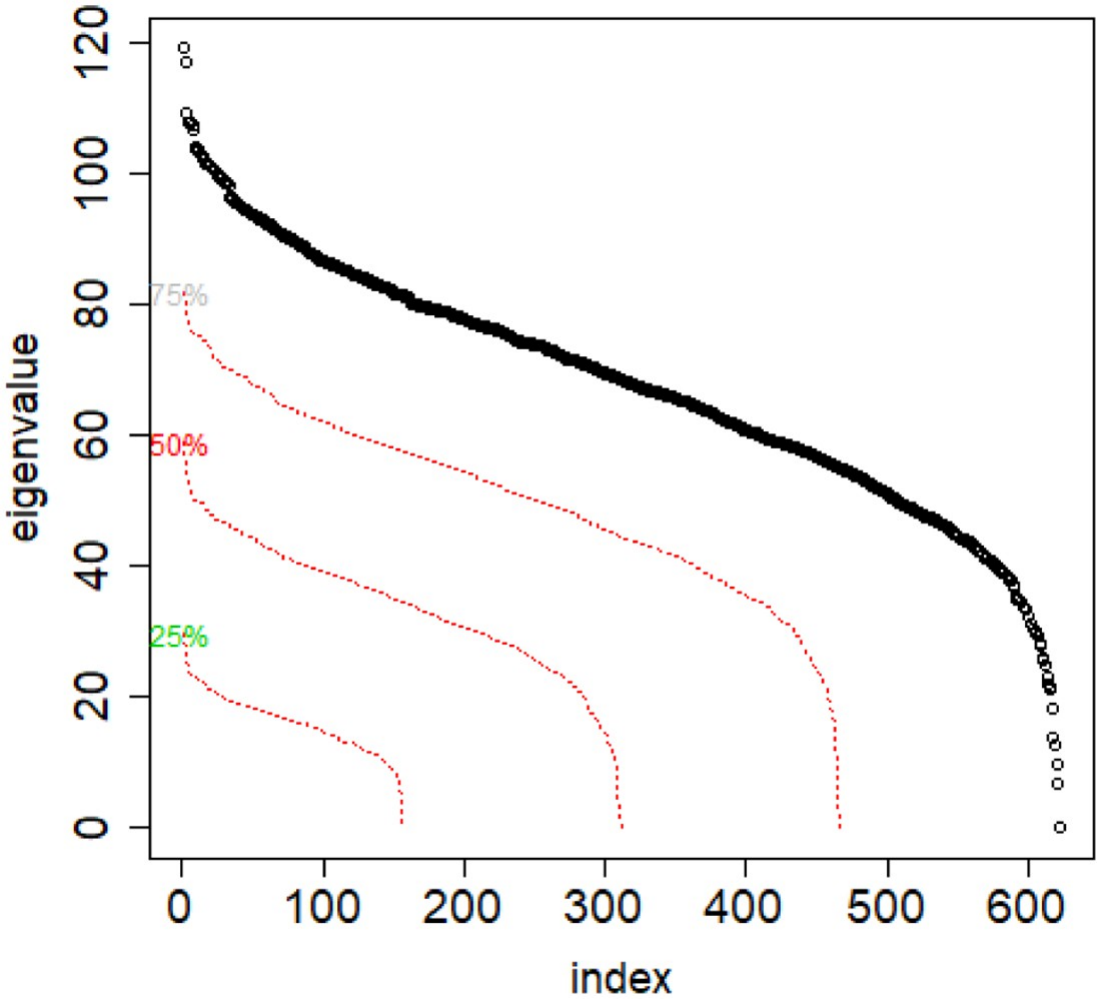
Combinatorial Laplacian spectrum of the class #pizgaczhell of TWT.PL data set and of samples of size 25%, 50% and 75%.

Before doing so, let us describe our findings when experimentally looking at the data we mentioned in Section 2. Various classes of objects belonging to the same dataset have distinct distributions of their eigenvalues of the combinatorial and normalized Laplacians of their similarity matrices. See Fig 10. The first reason why they differ is because the number of elements of the distinct classes differ. Therefore the length of each eigenvalue vector differs. What is more, even if we take sample from a class and compare it to this same class as a whole, then also the spectra are different (though the shapes are now similar). See Fig 11.

**Fig 12:**
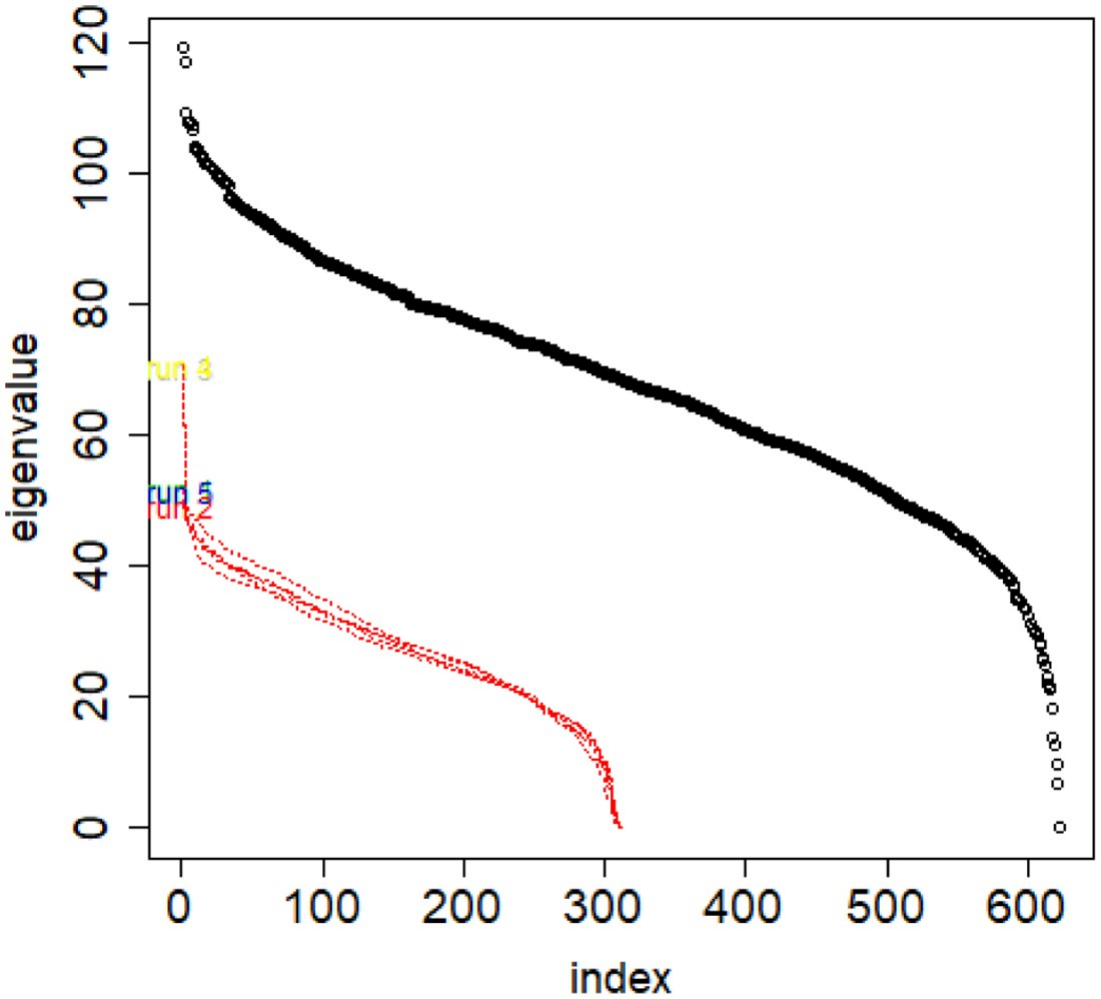
Combinatorial Laplacian spectrum of the class #pizgaczhell of TWT.PL data set and several samples of size 50%.

**Fig 13:**
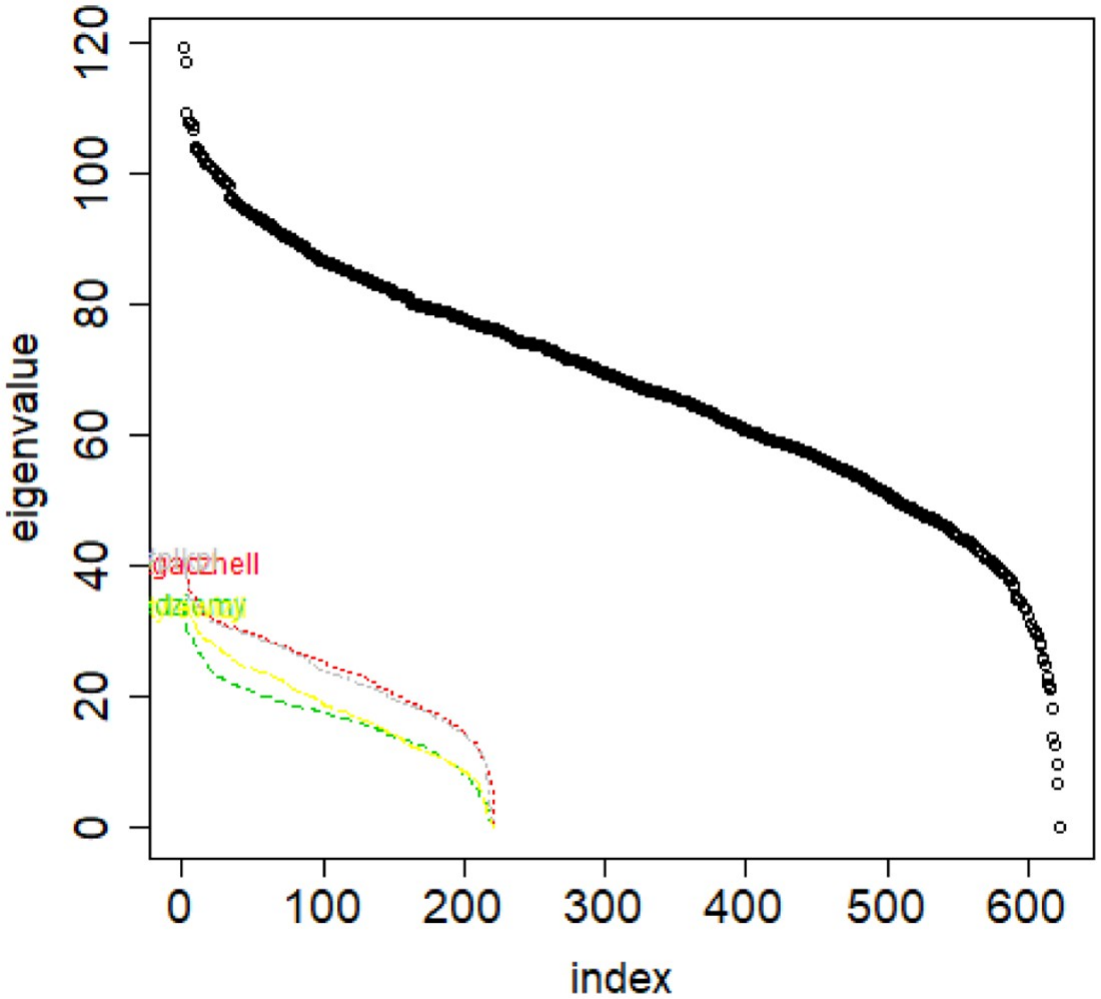
Combinatorial Laplacian spectrum of the class #pizgaczhell of TWT.PL data set and several samples of same size (size of the smallest class) from different classes.

However, when investigating one concrete class, if we annihilate this difference by sampling the same class with identical sample sizes, then their vectors of eigenvalues are of equal length, but also the eigenvalues on same positions in both vectors are close to one another, see Fig 12. But if we take same size samples from different classes, the distributions are different. See Fig 13.

### 7.2 The similarity of eigenvalue distributions of combinatorial Laplacian of a document class and its subsets

Let us explain the reason for this behaviour with our theoretical model of the “stable” distribution of similarities within the same class of documents. Let *X*_1_, *X*_2_ be equally sized, “sufficiently large” samples from *X* without intersection. Let the similarity matrix over *X*_1_ × *X*_2_ be *S*. Note that given a finite set of words, we get a finite set of possible similarity measures, e.g. cosine similarities. The probability distribution of words in the class implies the probability distribution of similarities between documents of the same class. Under “sufficiently” large samples *X*_1_, *X*_2_, the similarity matrix *S* approximates this similarity distribution at least in the expectation. We will omit, however, the expectation symbol in order to avoid a too complex notation.

Due to our assumptions, also *S*_1_ = *S* is the similarity matrix over *X*_1_ × *X*_1_ and and *S*_2_ = *S* over *X*_2_ × *X*_2_*except for the diagonal*, as the self-similarity follows a different pattern, but when we compute combinatorial Laplacian for *S*_1_, *S*_2_, the diagonal elements do not matter, hence the assumption *S*_1_ = *S*_2_ = *S* is justified. Under the “large collection assumption”, the similarity matrix *B* of *X*_1_∪*X*_2_ × *X*_1_∪*X*_2_ would have the form:

$$\begin{array}{c} B=[\begin{array}{cc} S & S\\ S & S \end{array}] \end{array}$$

Let *L*(*S*) be the combinatorial Laplacian of *S*. Let λ be an eigenvalue associated with the eigenvector **v** of *L*(*S*). Let *d*(*S*) be the diagonal of matrix of *S*, and *D*(*S*) be the diagonal matrix where each diagonal element corresponds to column sum of *S*. As can be seen from Section C in S1 Appendix, with this notation, if (λ, **v**) is the eigenpair of the Laplacian *L*(*S*), then we get

$$\begin{array}{c} L\left(B\right)[\begin{array}{c} v\\ v \end{array}]=2\lambda [\begin{array}{c} v\\ v \end{array}] \end{array}$$ (3)

which means that 2λ is the eigenvalue of *L*(*B*) and (**v**^*T*^, **v**^*T*^)^*T*^ is its eigenvector. It turns out that for twice as big “exact” samples from some document set with a well defined “style”, or “theme”, or “topic”, as used in PLSA or LDA document analysis, have twice as big eigenvalues. Same can be repeated for splitting the dataset into more equally sized subsets. This fact justifies the usage of sample size normalization which we apply in our algorithm.

Let us make the remark, that, as shown in the Section D in S1 Appendix, there is no way for expressing the normalized Laplacian L(B-d(B)) in terms of the normalized Laplacian L(S-d(S)) and therefore, the respective classification results will be approximate only. Maybe this insight constitutes a hint that the concept of normalized Laplacian needs to be revisited or at least considered in two versions, as pointed at in the Section D in S1 Appendix.

**Fig 14:**
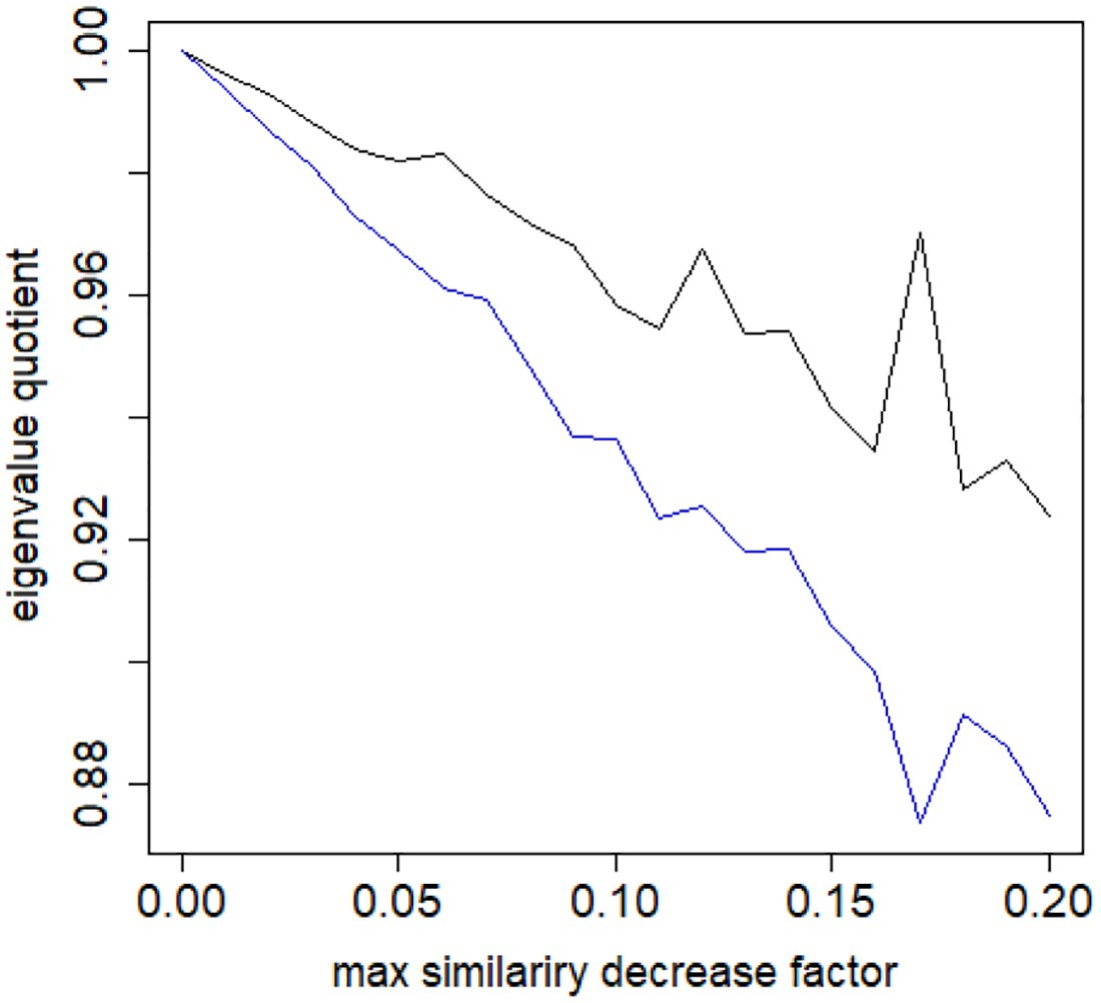
Effects of similarity perturbation within a class of data. Upper line represents the maximal quotient and the lower line represents the minimal quotient of eigenvalues after and before perturbing the similarity matrix, as described in the text, depending on the maximal similarity decrease factor. ANO.8 dataset.

Going back to combinatorial Laplacian, let us now soften the assumption that the subsamples of the dataset have exactly the same distribution of similarities and let us allow for a slight deviation from it. This corresponds also to the reality that the drawn samples will not have exactly the same similarity distribution and the question is then what is the impact of variation of these similarities. We performed the following experiment: We took the ANO.8 set and computed its (cosine) similarity matrix *S*. Next, for values of the parameter *limitation* in the range [0, 0.2] (in steps of 0.01) we produced a perturbation *S*′ of this matrix *S* in that off-diagonal elements of *S* were multiplied by random factors sampled from the range [1 − *limitation*, 1]. The eigenvalues λ′ of *S*′ were computed and then the corresponding eigenvalues of *S*′ and *S* were divided (λ′/λ). The minima and maxima of these quotients were reported in Fig 14. The eigenvalue quotients seem to be delimited by the *limitation* parameter. We can conclude that small perturbations in the similarities of a collection of documents do not change significantly the eigenvalues. This insight is in line with Weyl’s inequality [43].

Summarizing, we hypothesize that (sub)sets of documents following some general characterization of their mutual similarity (common topic or theme) will follow some stable pattern in spite of perturbations.

### 7.3 Similarity of eigenvalue spectra of Laplacians of slightly disturbed similarity matrices

The aforementioned phenomenon can be partially explained by the following reasoning. Consider a combinatorial Laplacian *L* of a graph with *n* nodes. Let its eigenvectors be denoted as **v**_**1**_, **v**_**2**_, …, **v**_**n**_, whereby all eigenvectors are of unit length and their corresponding eigenvalues are in non-decreasing order 0 ≤ λ_1_ ≤ λ_2_ ≤ … ≤ λ_*n*_.

Now consider, without loosing generality, the following Laplacian *L*′ of a graph with the similarity matrix *S* of *n* objects (of dimension *S* *n* × *n*).

$$\begin{array}{c} L^{\prime }=[\begin{array}{ccc} a+b^{\Sigma } & -a & -b^{T}\\ -a & a+c^{\Sigma } & -c^{T}\\ -b & -c & D \end{array}] \end{array}$$

where *a* = *S*_1,2_ = *S*_2,1_, **b** = *S*_3:*n*,1_, **c** = *S*_3:*n*,2_, **b**^*Σ*^ = ∑_*i*_*b*_*i*_, **c**^*Σ*^ = ∑_*i*_*c*_*i*_, *D* = *S*_3:*n*,3: *n*_ Let *L*′′ be a Laplacian of a similar graph with same connections as *L*′ but except that the similarity between objects 1 and 2 is increased by *x* > 0.

$$\begin{array}{c} L^{\prime \prime }=[\begin{array}{ccc} a+x+b^{\Sigma } & -a-x & -b^{T}\\ -a-x & a+x+c^{\Sigma } & -c^{T}\\ -b & -c & D \end{array}] \end{array}$$

Let us denote *X* = *L*′′ − *L*′. Note that the matrix *X* looks essentially like a Laplacian of a graph of *n* nodes where only the first two are connected with edge weight *x*. So all its eigenvalues are non-negative. Let us denote the eigenvalues of *X* with 0 ≤ *ξ*_1_ ≤ … ≤ *ξ*_*n*_ whereby *ξ*_*n*_ = 2*x* and the other ones are zero. According to Weyl’s inequality about perturbation [43, Sec.8.1.2] have that for each *i* $\lambda_{i}^{\prime }+\xi_{1}\leq \lambda_{i}^{\prime \prime }\leq \lambda_{i}^{\prime }+\xi_{n}$. This means in practice (as *ξ*_1_ = 0) that all the eigenvalues of *L*′′ are not smaller than the corresponding eigenvalues of *L*′.

In general, if *L*′′ would be a Laplacian of a graph over the same set of nodes as *L*′ with similarity matrix *S*′′ such that each entry in *S*′′ is greater or equal to the corresponding entry in *S*, then all the eigenvalues of *L*′′ are not smaller than the corresponding eigenvalues of *L*′ (by simple induction).

Consider now a similarity matrix *S*′′′ = *S*/(1 − *τ*) where 0 < *τ* < 1. Its Laplacian *L*′′ has then the property *L*′′′ = *L*′/(1 − *τ*) and therefore its eigenvalues have the property $\lambda_{i}^{\prime \prime \prime }=\lambda_{i}^{\prime }/\left(1-\tau \right)$ hence for any *S*′′ such that for each *i*, *j*: $S_{i,j}\leq S_{i,j}^{\prime \prime }\leq S_{i,j}^{\prime \prime \prime }/\left(1-\tau \right)$ we have $\lambda_{i}^{\prime }\leq \lambda_{i}^{\prime \prime }\leq \lambda_{i}^{\prime \prime \prime }=\lambda_{i}^{\prime }/\left(1-\tau \right)$, as exemplified by the mentioned Fig 14. A bit different but similar insight (for a more general form of the difference matrix) was stated in [43], that is, if *L* and *L* + *X* are symmetric matrices *n* × *n* then for each *j* we have |λ_*j*_(*L*) − λ_*j*_(*L*_*X*_)| ≤ ‖*X*‖_2_ [43] (as reported as Theorem 8.1.8 therein).

The above observation made us consider the set of eigenvalues not as a vector, but rather as a function λ:[0,1]→R of a “normalized index”, that is for each eigenvalue λ_*i*_ on the position *i* in the vector of eigenvalues of length *l* we have λ(*i*/(*l* − 1)) = λ_*i*_, and for otherwise λ(*x*) is a linear interpolation of the in-between-points. λ_*i*_ are deemed to be sorted decreasingly, with the index *i* of the first position being equal to 0.

Based on the above assumption, we can compute a “distance” between a given new sample and the elements of a class for the normalized Laplacians. This “distance” is the area between the λ curves of the sample and the class (Normalized Laplacian Method). See Fig 4.

However, this is insufficient when dealing with combinatorial Laplacian, because subsamples of different sizes from the same class have similar shapes but they are stretched differently along the *Y* axis. Therefore, a further normalization is needed. One approach is to define a function λ_*CLRL*_: [0, 1] → [0, 1] in such a way that $\lambda_{CLRL}\left(i/\left(l-1\right)\right)=\frac{\lambda_{i}}{\lambda_{0}}$. The linear interpolation is applied as previously. This approach is used in the Combinatorial Laplacian Relative Lambda Method. See Fig 2.

Based on the above assumption, we can compute a “distance” between a given new sample and the elements of a class as the area between the λ_*CLRL*_ curves of the sample and the class.

But we can also notice that the stretching along the *Y* axis is proportional to the sample size. So we proposed a function $\lambda_{CLRL}:\left[0,1\right]\to R$ in such a way that $\lambda_{CLSSAL}\left(i/\left(l-1\right)\right)=\frac{\lambda_{i}}{l}$. The linear interpolation is applied as previously. This approach is used in the Combinatorial Laplacian Sample Size Adjusted Lambda Method. See Fig 3.

Based on the above assumption, we can compute a “distance” between a given new sample and the elements of a class as the area between the λ_*CLSSAL*_ curves of the sample and the class.

Last but not least we saw in the data that usually the classes differed by their λ_*CLSSAL*_(0) values. So in the CLMXL, we use the absolute difference between λ_*CLSSAL*_(0) for the sample and the class as the measure of distance. See Fig 3.

The class assigned to the sample is the class closest to the sample.

### 7.4 The algorithm

The Algorithm 1 presents in a compact way the described method bundle. The functions called there, that is *L*(), *spectrum*(), *specfun*(), *spectdist*() are described below.

**Algorithm 1:** The eigenvalue based classification algorithm

**Data:** *S*—similarity matrix of the new set of documents

S —set of similarity matrices of the classes of documents to which to classify into

**Result:** *k*—the assigned class of documents

*L* ≔ *L*(*S*)—Compute Laplacian;

L:=L(S) —Compute Laplacians;

*E* ≔ *spectrum*(*L*)—Compute Laplacian eigenvalues;

E:=spectrum(L) —Compute Laplacian eigenvalue for each Laplacian from L;

*F* ≔ *specfun*(*E*)—transform a spectrum into a function;

F≔specfun(E)—transform spectra into functions;

*K* ← number of classes in S;

*k* ← −1;

*mndist* ← ∞;

**for** *j* ← 1 **to** *K* **do**

 $distance\leftarrow spectdist(F,F_{j})$,

 **if** *distance* < *mndist* **then**

  *k* ← *j*;

  *mndist* ← *distance*;

 **else**

  do nothing;

 **end**

**end**

A drawback of this approach is that *S* must be a homogeneous group, but there exist practical applications where this is the case.

Note that this approach to distance computation between spectra bears some resemblance to Dynamic Time Warping (DTW) distance, but the difference is that we apply a linear transformation to the index axis of the spectrogram, while DTW encourages non-linear transformations.

In the Algorithm 1, the

- *spectdist*(*F*_1_, *F*_2_) function is the area between the two functions *F*_1_, *F*_2_ being its arguments for the function domains [0, 1], $\int_{0}^{1}\left|F_{1}\left(x\right)-F_{2}\left(x\right)\right|dx$, except for CLMXL, where |*F*_1_(0) − *F*_2_(0)| is returned.
- The function *L*(*S*) applied to the similarity matrix *S* is computed as *D*(*S*) − *S* except for Normalized Laplacian Method(NLL), where *D*(*S*)^−1/2^*Z*(*D*(*S*) − *S*)*D*(*S*)^−1/2^*Z* is the result.
- The function *spectrum*(*L*) applied to Laplacian *L* returns a vector of eigenvalues of *L* in non-decreasing order.
- The function specfun(*E*) applied to the spectrum *E* of a Laplacian returns a function *F*(*x*) defined in the domain *x* ∈ [0, 1] with properties depending on the type of classification method. Let *E* = [λ_1_, …, λ_*n*_], whereby 0 = λ_1_ ≤ … ≤ λ_*n*_
  - for CLRL:
    $$F(\frac{n-i}{n-1})=\frac{\lambda_{i}}{\lambda_{n}}$$
  - for CLSSAL and CLMXL:
    $$F(\frac{n-i}{n-1})=\frac{\lambda_{i}}{n}$$
  - for NLL:
    $$F(\frac{n-i}{n-1})=\lambda_{i}$$
  and otherwise for any $x\in [\frac{n-(i+1)}{n-1},\frac{n-i}{n-1}]$
  $$F\left(x\right)=F(\frac{n-(i+1)}{n-1})\cdot (x-\frac{n-(i+1)}{n-1})+F(\frac{n-i}{n-1})\cdot (\frac{n-i}{n-1}-x)$$

## 8 Classification experiments

### 8.1 Experimental setup

To validate the proposed approach, we created eigenvalue spectrum models for each class of each data set listed in the data section 2, for the proposed classification algorithm with each variant, that is CLRL, CLSSAL, CLMXL and NLL.

Then we sampled 100 times each class of each dataset and classified it in the context of that dataset using each of the classification methods CLRL, CLSSAL, CLMXL and NLL. The results are summarized in Table 19.

**Table 19:** Error percentage and F1 for eigenvalue based classification. Column names: datasets, row names: GSC methods considered.

set/measure	CLRL	CLRL	CLSSAL	CLSSAL	CLMXL	CLMXL	NLL	NLL
	error	F1	error	F1	error	F1	error	F1
ANO.8	24	75.81	25.2	74.91	33.8	65.93	33.8	57.93
ANO.26	30.55	67.85	29	69.85	52	46.59	68.09	24.94
ANO.44	35	65.11	14.75	85.34	16.5	83.94	60	30.11
ANO.94	0	100	0	100	2	98	59.33	29.66
SEN.EN.maj	28	69.8	0.5	99.5	21.5	77.65	44.5	44.51
SEN.EN.ent	2.67	97.34	6.67	93.28	19.33	80.57	16.67	83.2
SEN.PL.maj	11.5	88.44	9.5	90.5	26	73.96	50	33.34
SEN.PL.ent	43.4	54.62	31.8	62.85	56.4	43.78	71	20.4
TWT.EN	22.4	77.2	22.8	76.84	33.8	63.99	80	6.67
TWT.PL	29	70.8	12.25	87.67	32.25	67.18	72.25	17.45
BLK.4.0.2.0.5	61.5	29.33	0	100	11.25	88.53	75	10

### 8.2 Results

One sees that, except for the dataset SEN.EN.ent and TWT.EN, the CLSSAL performed best, while the NLL was the worst except for TWT.PL.

Generally, in the presented classification experiments, Combinatorial Laplacian Sample Size Adjusted Lambda Method and Combinatorial Laplacian Relative Lambda Method competed yielding best results among all four methods. Normalized Laplacian Method performed poorly so that it is in no way recommended for classification purposes as defined in this paper.

## 9 Discussion

Algorithms based on spectral properties of matrices describing problem under consideration are used in various branches of science. Anomaly detection methods are used for example in radiology [44]. Various classification methods (like SVM) can be reformulated in terms seeking eigenvalues, see e.g. [45, 46]. However, the vast majority of spectral analysis in AI, Graph Spectral Analysis (GSA) is devoted to graph spectral clustering methods which assume that the domain is formulated in terms of a graph and the clustering problem to solve is understood as optimal graph cutting. Only a very narrow stream of research within GSA deals with harnessing these methods to the task of classification, like [9–11, 16, 18, 19, 22] and other. There exists also the research on harnessing classification to enable clustering of larger graphs or graphs that are extended, for example [5–8].

Our research differs from these efforts in the following way:

- Within GSA, both clustering and classification is focused on exploitation of eigenvectors, while the eigenvalues serve the selection of eigenvectors. In our paper, we do not use eigenvectors, but rather the whole spectrum of eigenvalues.
- Classification methods from outside of GSA reformulate classification problems in terms of finding eigenvalues, or try to find some signals in the spectrum, while we use the spectra of groups of objects to decide if they belong to the same class or not.
- Usually, classification methods are applied to single objects. Our method classifies a group of objects into a pre-existing class.
- We have demonstrated that objects (documents) coming from the same document generator have approximately a similar eigenvalue spectrum, subject to scaling based on group size.
- Classical approaches to classification within GSA were shown in the paper to fail for several real-world datasets. Our method worked for them much better.
- These failures may be at least partially attributed to mass concentration as well as noise in eigenvectors associated with low eigenvalues. Kernel methods, mentioned in section 3.3, as based also on those eigenvectors, would suffer from same shortcomings. Multiplication with inverse square roots of these eigenvalues may make the problems even more severe.
- The proposed method can be used for elaboration of new large dataset clustering methods, in that chunks of data are clustered via GSA, and then our method is applied to classify new data chunks to categories of previous ones.

Our methodology has a number of advantages over the existing approaches to spectral classification. Though “natural classification” with nsc.ursvddp is a clear winner over our best approach CLSSAL in case of ANO.* sets, none of them can compete in case of SEN.*, TWT.* and BLK.* datasets. Situation is similar in case of “cluster-based classification”.

The spectral eigenvector based classification seems to perform very well in case of SEN.* datasets and at some ANO.* datasets, clearly beating CLSSAL. But it does not perform well in other cases.

A limitation of our methods is the inability to classify a single new document, only (sufficiently large) groups of documents can be assigned to pre-existing classes. The spectral eigenvector based classification methods do not have this disadvantage. However, it is impossible just to take a new document and apply some distance measure, because in these methods a new document is not placed in the same vector space as the trained model. In fact, in order to classify a new document, you need to perform spectral decomposition of the model data plus the new document and then train the model again, and only afterwards you can classify the new document. This may be time consuming. In case of CLSSAL one has to perform spectral decomposition of the package of new documents only prior to classification. In case of “natural classification” and “cluster-based classification”. The same cumbersome spectral decomposition is needed as in case of spectral eigenvector based classification methods.

So one has to state that CLSSAL is in general more realistic than the competition.

In brief, we proposed a completely new way of looking at eigenvalue spectrum within GSA. Traditional algorithms separate groups of objects based on the eigenvector elements, their threshold. Our method characterizes a class of objects via eigenvalue spectrum. We found no comparable approach in the literature.

## 10 Conclusions

In this paper, we have presented a new classification method based on spectral clustering. The method exploits the eigenvalue spectrum, a feature that has been neglected so far in the scientific investigations. The method is suited for “bulk” decision making that is if there are groups of objects to be assigned to a class as a whole, as we sometimes encounter when classifying products in large scale supermarket chains, where the number of products amounts to hundreds of thousands and where the products constitute clearly defined low level bundles that need however to be assigned to higher level classes, e.g. for high level decision making or for outside reporting.

We have demonstrated that there exist some problems when applying classical spectral cluster analysis to real-world datasets for a broad range of applications, including product descriptions, news headlines, tweets and other relatively short messages. We have pointed to the following problems that give rise to inadequate “natural classification”: concentration of mass in the eigenvectors associated with low eigenvalues which in turn may be caused by noise in the range of low eigenvalues. This noise becomes a problem because the classical approaches to spectral analysis (both clustering and classification) rely on those eigenvectors associated with low eigenvalues. We have shown a pathway to escape the problem partially by performing SVD analysis and setting low eigenvalues to zero. This needs however a further investigation because the choice of the portion of eigenvalues for this operation is not clear for now.

We have demonstrated that the two new classification methods, based on classical combinatorial eigenvalue spectra (and relative eigenvalue normalization or population size normalization) exhibits a reasonable performance given sufficiently large data portions to classify and sufficient differences between the classes of objects (documents) under investigation.

This research may shed some light on the efforts to broaden spectral analysis to large scale datasets, e.g. a strategy may be proposed to cluster smaller data sets and then to merge the chunks via classification methods proposed here.

Future research will also explore the relation between SVD results and the classifier performance.

## Supporting information

S1 File File contains the zipped XLSX file BLK.4_0.2_0.5clusters. The file contains dataset BLK.4_0.2_0.5 referred to in Section 2.
(ZIP)

S2 File File contains the zipped XLSX file ANO.94clusters. The file contains dataset ANO.94clusters referred to in Section 2.
(ZIP)

S3 File File contains the other zipped XLSX files of datasets. The file contains datasets TWT.EN, TWT.PL, SEN.EN.ent, SEN.EN.maj, SEN.PL.ent, SEN.PL.maj, ANO.8, ANO.26 and ANO.44 in XLSX files named following the same convention as above. The datasets are referred to in Section 2.
(ZIP)

S4 File File contains the zipped set of figures for this paper in PNG format. S4_1_Fig.png: Distribution of objects in the space spanned by the eigenvectors of combinatorial Laplacian corresponding to some of the lowest eigenvalues (no. 1490 and 1488)—TWT.PL dataset: in two corners there are two objects, while the rest is located in the third corner (mass concentration). The positions of datapoints are slightly blurred so that the mass concentration is visible. S4_2_Fig.png: The artificial data set BLK.4_0.2_0.5—adjacency matrix for documents S4_3_Fig.png: S4_3_FigButtom.png: Mass of the element with the largest mass in the eigenvector. Eigenvectors are ordered by decreasing eigenvalue. Top figure: the entire spectrum. Bottom figure: only the 50 eigenvectors corresponding to 50 lowest eigenvalues. English Twitter data TWT.EN. S4_4_Fig.png: S4_4_FigButtom.png: Number of highest mass elements of eigenvectors constituting half mass of the eigenvector. English Twitter data TWT.EN. Top: all eigenvectors. Bottom: 50 eigenvectors with the lowest eigenvalue. S4_5_Fig.png: S4_5_FigButtom.png: Relative error among the elements of eigenvector constituting its halfmass (standard error divided by the mean). English Twitter data TWT.EN. Top: all eigenvectors. Bottom: 50 eigenvectors with the lowest eigenvalue. S4_6_Fig.png: S4_6_FigButtom.png: Eigenvalue distributions for the entire dataset (the black dots) and for the classes (lines with different colors) for combinatorial (top) and normalized (bottom) graph Laplacian. English Twitter data TWT.EN. On the bottom, ten lowest eigenvalues were omitted for better readability. S4_7_Fig.png: Combinatorial Laplacian of the entire TWT.PL data set (thick line) and of each of the classes. S4_8_Fig.png: Combinatorial Laplacian spectrum of the class #pizgaczhell of TWT.PL data set and of samples of size 25%, 50% and 75%. S4_9_Fig.png: Combinatorial Laplacian spectrum of the class #pizgaczhell of TWT.PL data set and several samples of size 50%. S4_10_Fig.png: Combinatorial Laplacian spectrum of the class #pizgaczhell of TWT.PL data set and several samples of same size (size of the smallest class) from different classes. S4_11_Fig.png: Spectral normalization in the Combinatorial Laplacian Relative Lambda Method method. The TWT.PL dataset S4_12_Fig.png: Spectral normalization in the Combinatorial Laplacian Sample Size Adjusted Lambda Method method and Combinatorial Laplacian Sample Size Adjusted Maximum Lambda Method method. The TWT.PL dataset. S4_13_Fig.png: Spectral normalization in the Normalized Laplacian Method method The TWT.PL dataset. S4_14_Fig.png: Effects of similarity perturbation within a class of data. Upper line represents the maximal quotient and the lower line represents the minimal quotient of eigenvalues after and before perturbing the similarity matrix, as described in the text, depending on the maximal similarity decrease factor. ANO.8 dataset.
(ZIP)

S5 File File contains the zipped set of figures for this paper in TIFF format. Names and captions are exactly the same as in S4 File, except that the extension is now TIFF.
(ZIP)

S1 Appendix (PDF)
