# Supplementary figures and images for: Eigenvalue based spectral classification
Source: PLoS One. 2023 Apr 6;18(4):e0283413. doi: 10.1371/journal.pone.0283413 (PMC10079090; doi:10.1371/journal.pone.0283413)

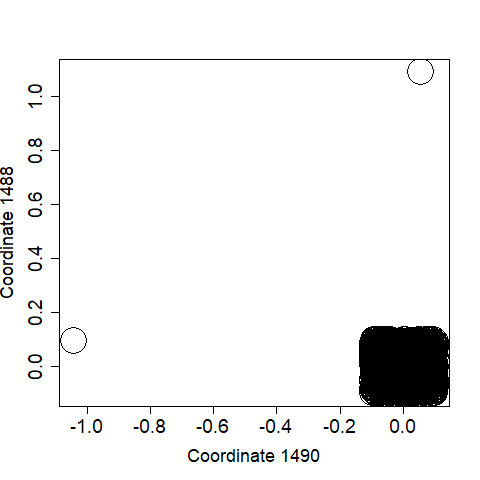

Supplement: S4 File — S4_1_Fig.png: Distribution of objects in the space spanned by the eigenvectors of combinatorial Laplacian corresponding to some of the lowest eigenvalues (no. 1490 and 1488)—TWT.PL dataset: in two corners there are two objects, while the rest is located in the third corner (mass concentration). The positions of datapoints are slightly blurred so that the mass concentration is visible. S4_2_Fig.png: The artificial data set BLK.4_0.2_0.5—adjacency matrix for documents S4_3_Fig.png: S4_3_FigButtom.png: Mass of the element with the largest mass in the eigenvector. Eigenvectors are ordered by decreasing eigenvalue. Top figure: the entire spectrum. Bottom figure: only the 50 eigenvectors corresponding to 50 lowest eigenvalues. English Twitter data TWT.EN. S4_4_Fig.png: S4_4_FigButtom.png: Number of highest mass elements of eigenvectors constituting half mass of the eigenvector. English Twitter data TWT.EN. Top: all eigenvectors. Bottom: 50 eigenvectors with the lowest eigenvalue. S4_5_Fig.png: S4_5_FigButtom.png: Relative error among the elements of eigenvector constituting its halfmass (standard error divided by the mean). English Twitter data TWT.EN. Top: all eigenvectors. Bottom: 50 eigenvectors with the lowest eigenvalue. S4_6_Fig.png: S4_6_FigButtom.png: Eigenvalue distributions for the entire dataset (the black dots) and for the classes (lines with different colors) for combinatorial (top) and normalized (bottom) graph Laplacian. English Twitter data TWT.EN. On the bottom, ten lowest eigenvalues were omitted for better readability. S4_7_Fig.png: Combinatorial Laplacian of the entire TWT.PL data set (thick line) and of each of the classes. S4_8_Fig.png: Combinatorial Laplacian spectrum of the class #pizgaczhell of TWT.PL data set and of samples of size 25%, 50% and 75%. S4_9_Fig.png: Combinatorial Laplacian spectrum of the class #pizgaczhell of TWT.PL data set and several samples of size 50%. S4_10_Fig.png: Combinatorial Laplacian spectrum of the class #pizgaczhell o [file pone.0283413.s004.zip › S4_1_Fig.png]

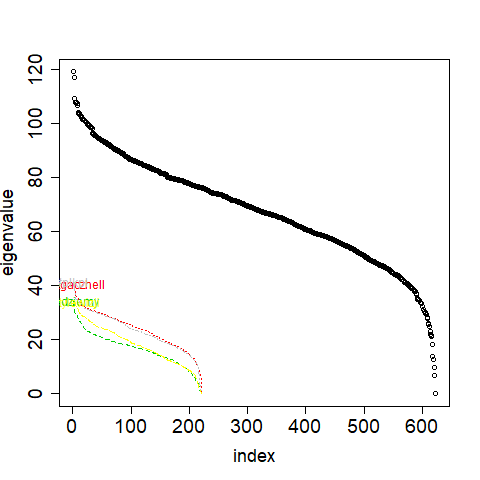

Supplement: S4 File — S4_1_Fig.png: Distribution of objects in the space spanned by the eigenvectors of combinatorial Laplacian corresponding to some of the lowest eigenvalues (no. 1490 and 1488)—TWT.PL dataset: in two corners there are two objects, while the rest is located in the third corner (mass concentration). The positions of datapoints are slightly blurred so that the mass concentration is visible. S4_2_Fig.png: The artificial data set BLK.4_0.2_0.5—adjacency matrix for documents S4_3_Fig.png: S4_3_FigButtom.png: Mass of the element with the largest mass in the eigenvector. Eigenvectors are ordered by decreasing eigenvalue. Top figure: the entire spectrum. Bottom figure: only the 50 eigenvectors corresponding to 50 lowest eigenvalues. English Twitter data TWT.EN. S4_4_Fig.png: S4_4_FigButtom.png: Number of highest mass elements of eigenvectors constituting half mass of the eigenvector. English Twitter data TWT.EN. Top: all eigenvectors. Bottom: 50 eigenvectors with the lowest eigenvalue. S4_5_Fig.png: S4_5_FigButtom.png: Relative error among the elements of eigenvector constituting its halfmass (standard error divided by the mean). English Twitter data TWT.EN. Top: all eigenvectors. Bottom: 50 eigenvectors with the lowest eigenvalue. S4_6_Fig.png: S4_6_FigButtom.png: Eigenvalue distributions for the entire dataset (the black dots) and for the classes (lines with different colors) for combinatorial (top) and normalized (bottom) graph Laplacian. English Twitter data TWT.EN. On the bottom, ten lowest eigenvalues were omitted for better readability. S4_7_Fig.png: Combinatorial Laplacian of the entire TWT.PL data set (thick line) and of each of the classes. S4_8_Fig.png: Combinatorial Laplacian spectrum of the class #pizgaczhell of TWT.PL data set and of samples of size 25%, 50% and 75%. S4_9_Fig.png: Combinatorial Laplacian spectrum of the class #pizgaczhell of TWT.PL data set and several samples of size 50%. S4_10_Fig.png: Combinatorial Laplacian spectrum of the class #pizgaczhell o [file pone.0283413.s004.zip › S4_10_Fig.png]

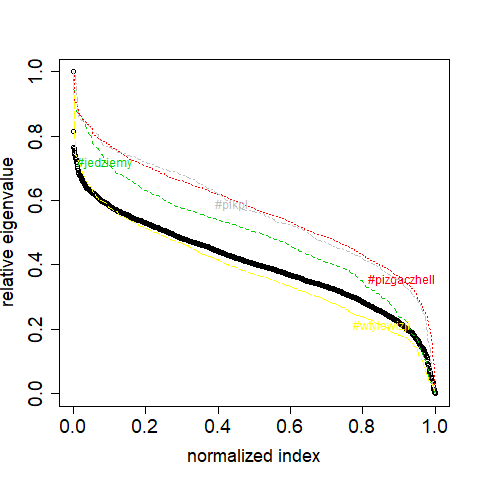

Supplement: S4 File — S4_1_Fig.png: Distribution of objects in the space spanned by the eigenvectors of combinatorial Laplacian corresponding to some of the lowest eigenvalues (no. 1490 and 1488)—TWT.PL dataset: in two corners there are two objects, while the rest is located in the third corner (mass concentration). The positions of datapoints are slightly blurred so that the mass concentration is visible. S4_2_Fig.png: The artificial data set BLK.4_0.2_0.5—adjacency matrix for documents S4_3_Fig.png: S4_3_FigButtom.png: Mass of the element with the largest mass in the eigenvector. Eigenvectors are ordered by decreasing eigenvalue. Top figure: the entire spectrum. Bottom figure: only the 50 eigenvectors corresponding to 50 lowest eigenvalues. English Twitter data TWT.EN. S4_4_Fig.png: S4_4_FigButtom.png: Number of highest mass elements of eigenvectors constituting half mass of the eigenvector. English Twitter data TWT.EN. Top: all eigenvectors. Bottom: 50 eigenvectors with the lowest eigenvalue. S4_5_Fig.png: S4_5_FigButtom.png: Relative error among the elements of eigenvector constituting its halfmass (standard error divided by the mean). English Twitter data TWT.EN. Top: all eigenvectors. Bottom: 50 eigenvectors with the lowest eigenvalue. S4_6_Fig.png: S4_6_FigButtom.png: Eigenvalue distributions for the entire dataset (the black dots) and for the classes (lines with different colors) for combinatorial (top) and normalized (bottom) graph Laplacian. English Twitter data TWT.EN. On the bottom, ten lowest eigenvalues were omitted for better readability. S4_7_Fig.png: Combinatorial Laplacian of the entire TWT.PL data set (thick line) and of each of the classes. S4_8_Fig.png: Combinatorial Laplacian spectrum of the class #pizgaczhell of TWT.PL data set and of samples of size 25%, 50% and 75%. S4_9_Fig.png: Combinatorial Laplacian spectrum of the class #pizgaczhell of TWT.PL data set and several samples of size 50%. S4_10_Fig.png: Combinatorial Laplacian spectrum of the class #pizgaczhell o [file pone.0283413.s004.zip › S4_11_Fig.png]

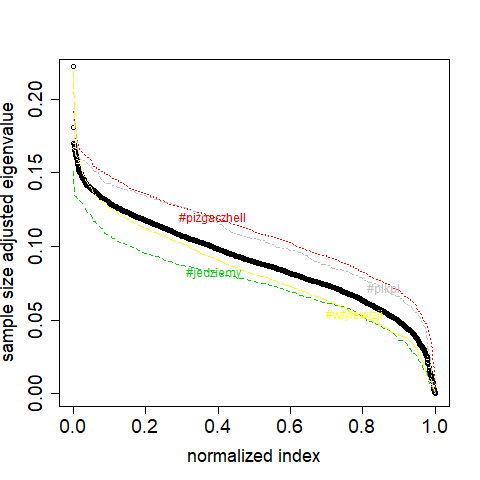

Supplement: S4 File — S4_1_Fig.png: Distribution of objects in the space spanned by the eigenvectors of combinatorial Laplacian corresponding to some of the lowest eigenvalues (no. 1490 and 1488)—TWT.PL dataset: in two corners there are two objects, while the rest is located in the third corner (mass concentration). The positions of datapoints are slightly blurred so that the mass concentration is visible. S4_2_Fig.png: The artificial data set BLK.4_0.2_0.5—adjacency matrix for documents S4_3_Fig.png: S4_3_FigButtom.png: Mass of the element with the largest mass in the eigenvector. Eigenvectors are ordered by decreasing eigenvalue. Top figure: the entire spectrum. Bottom figure: only the 50 eigenvectors corresponding to 50 lowest eigenvalues. English Twitter data TWT.EN. S4_4_Fig.png: S4_4_FigButtom.png: Number of highest mass elements of eigenvectors constituting half mass of the eigenvector. English Twitter data TWT.EN. Top: all eigenvectors. Bottom: 50 eigenvectors with the lowest eigenvalue. S4_5_Fig.png: S4_5_FigButtom.png: Relative error among the elements of eigenvector constituting its halfmass (standard error divided by the mean). English Twitter data TWT.EN. Top: all eigenvectors. Bottom: 50 eigenvectors with the lowest eigenvalue. S4_6_Fig.png: S4_6_FigButtom.png: Eigenvalue distributions for the entire dataset (the black dots) and for the classes (lines with different colors) for combinatorial (top) and normalized (bottom) graph Laplacian. English Twitter data TWT.EN. On the bottom, ten lowest eigenvalues were omitted for better readability. S4_7_Fig.png: Combinatorial Laplacian of the entire TWT.PL data set (thick line) and of each of the classes. S4_8_Fig.png: Combinatorial Laplacian spectrum of the class #pizgaczhell of TWT.PL data set and of samples of size 25%, 50% and 75%. S4_9_Fig.png: Combinatorial Laplacian spectrum of the class #pizgaczhell of TWT.PL data set and several samples of size 50%. S4_10_Fig.png: Combinatorial Laplacian spectrum of the class #pizgaczhell o [file pone.0283413.s004.zip › S4_12_Fig.png]

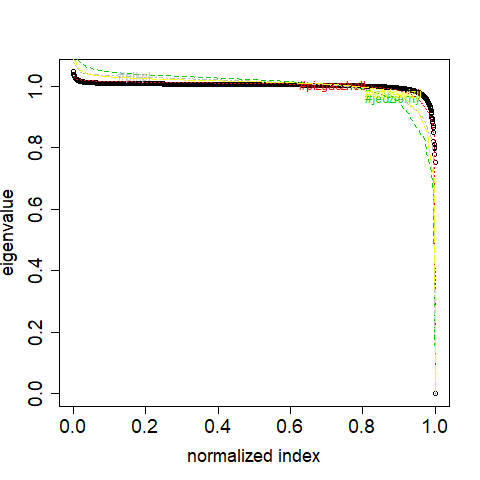

Supplement: S4 File — S4_1_Fig.png: Distribution of objects in the space spanned by the eigenvectors of combinatorial Laplacian corresponding to some of the lowest eigenvalues (no. 1490 and 1488)—TWT.PL dataset: in two corners there are two objects, while the rest is located in the third corner (mass concentration). The positions of datapoints are slightly blurred so that the mass concentration is visible. S4_2_Fig.png: The artificial data set BLK.4_0.2_0.5—adjacency matrix for documents S4_3_Fig.png: S4_3_FigButtom.png: Mass of the element with the largest mass in the eigenvector. Eigenvectors are ordered by decreasing eigenvalue. Top figure: the entire spectrum. Bottom figure: only the 50 eigenvectors corresponding to 50 lowest eigenvalues. English Twitter data TWT.EN. S4_4_Fig.png: S4_4_FigButtom.png: Number of highest mass elements of eigenvectors constituting half mass of the eigenvector. English Twitter data TWT.EN. Top: all eigenvectors. Bottom: 50 eigenvectors with the lowest eigenvalue. S4_5_Fig.png: S4_5_FigButtom.png: Relative error among the elements of eigenvector constituting its halfmass (standard error divided by the mean). English Twitter data TWT.EN. Top: all eigenvectors. Bottom: 50 eigenvectors with the lowest eigenvalue. S4_6_Fig.png: S4_6_FigButtom.png: Eigenvalue distributions for the entire dataset (the black dots) and for the classes (lines with different colors) for combinatorial (top) and normalized (bottom) graph Laplacian. English Twitter data TWT.EN. On the bottom, ten lowest eigenvalues were omitted for better readability. S4_7_Fig.png: Combinatorial Laplacian of the entire TWT.PL data set (thick line) and of each of the classes. S4_8_Fig.png: Combinatorial Laplacian spectrum of the class #pizgaczhell of TWT.PL data set and of samples of size 25%, 50% and 75%. S4_9_Fig.png: Combinatorial Laplacian spectrum of the class #pizgaczhell of TWT.PL data set and several samples of size 50%. S4_10_Fig.png: Combinatorial Laplacian spectrum of the class #pizgaczhell o [file pone.0283413.s004.zip › S4_13_Fig.png]

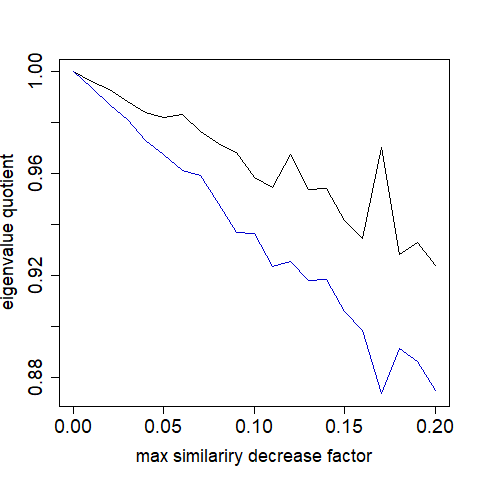

Supplement: S4 File — S4_1_Fig.png: Distribution of objects in the space spanned by the eigenvectors of combinatorial Laplacian corresponding to some of the lowest eigenvalues (no. 1490 and 1488)—TWT.PL dataset: in two corners there are two objects, while the rest is located in the third corner (mass concentration). The positions of datapoints are slightly blurred so that the mass concentration is visible. S4_2_Fig.png: The artificial data set BLK.4_0.2_0.5—adjacency matrix for documents S4_3_Fig.png: S4_3_FigButtom.png: Mass of the element with the largest mass in the eigenvector. Eigenvectors are ordered by decreasing eigenvalue. Top figure: the entire spectrum. Bottom figure: only the 50 eigenvectors corresponding to 50 lowest eigenvalues. English Twitter data TWT.EN. S4_4_Fig.png: S4_4_FigButtom.png: Number of highest mass elements of eigenvectors constituting half mass of the eigenvector. English Twitter data TWT.EN. Top: all eigenvectors. Bottom: 50 eigenvectors with the lowest eigenvalue. S4_5_Fig.png: S4_5_FigButtom.png: Relative error among the elements of eigenvector constituting its halfmass (standard error divided by the mean). English Twitter data TWT.EN. Top: all eigenvectors. Bottom: 50 eigenvectors with the lowest eigenvalue. S4_6_Fig.png: S4_6_FigButtom.png: Eigenvalue distributions for the entire dataset (the black dots) and for the classes (lines with different colors) for combinatorial (top) and normalized (bottom) graph Laplacian. English Twitter data TWT.EN. On the bottom, ten lowest eigenvalues were omitted for better readability. S4_7_Fig.png: Combinatorial Laplacian of the entire TWT.PL data set (thick line) and of each of the classes. S4_8_Fig.png: Combinatorial Laplacian spectrum of the class #pizgaczhell of TWT.PL data set and of samples of size 25%, 50% and 75%. S4_9_Fig.png: Combinatorial Laplacian spectrum of the class #pizgaczhell of TWT.PL data set and several samples of size 50%. S4_10_Fig.png: Combinatorial Laplacian spectrum of the class #pizgaczhell o [file pone.0283413.s004.zip › S4_14_Fig.png]

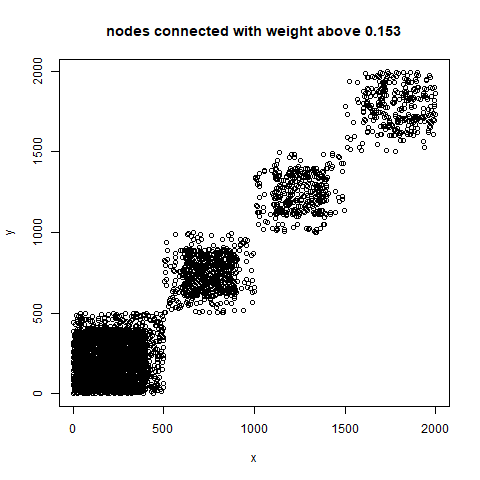

Supplement: S4 File — S4_1_Fig.png: Distribution of objects in the space spanned by the eigenvectors of combinatorial Laplacian corresponding to some of the lowest eigenvalues (no. 1490 and 1488)—TWT.PL dataset: in two corners there are two objects, while the rest is located in the third corner (mass concentration). The positions of datapoints are slightly blurred so that the mass concentration is visible. S4_2_Fig.png: The artificial data set BLK.4_0.2_0.5—adjacency matrix for documents S4_3_Fig.png: S4_3_FigButtom.png: Mass of the element with the largest mass in the eigenvector. Eigenvectors are ordered by decreasing eigenvalue. Top figure: the entire spectrum. Bottom figure: only the 50 eigenvectors corresponding to 50 lowest eigenvalues. English Twitter data TWT.EN. S4_4_Fig.png: S4_4_FigButtom.png: Number of highest mass elements of eigenvectors constituting half mass of the eigenvector. English Twitter data TWT.EN. Top: all eigenvectors. Bottom: 50 eigenvectors with the lowest eigenvalue. S4_5_Fig.png: S4_5_FigButtom.png: Relative error among the elements of eigenvector constituting its halfmass (standard error divided by the mean). English Twitter data TWT.EN. Top: all eigenvectors. Bottom: 50 eigenvectors with the lowest eigenvalue. S4_6_Fig.png: S4_6_FigButtom.png: Eigenvalue distributions for the entire dataset (the black dots) and for the classes (lines with different colors) for combinatorial (top) and normalized (bottom) graph Laplacian. English Twitter data TWT.EN. On the bottom, ten lowest eigenvalues were omitted for better readability. S4_7_Fig.png: Combinatorial Laplacian of the entire TWT.PL data set (thick line) and of each of the classes. S4_8_Fig.png: Combinatorial Laplacian spectrum of the class #pizgaczhell of TWT.PL data set and of samples of size 25%, 50% and 75%. S4_9_Fig.png: Combinatorial Laplacian spectrum of the class #pizgaczhell of TWT.PL data set and several samples of size 50%. S4_10_Fig.png: Combinatorial Laplacian spectrum of the class #pizgaczhell o [file pone.0283413.s004.zip › S4_2_Fig.png.png]

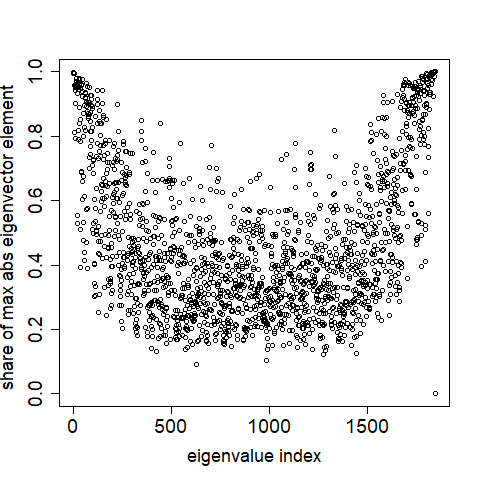

Supplement: S4 File — S4_1_Fig.png: Distribution of objects in the space spanned by the eigenvectors of combinatorial Laplacian corresponding to some of the lowest eigenvalues (no. 1490 and 1488)—TWT.PL dataset: in two corners there are two objects, while the rest is located in the third corner (mass concentration). The positions of datapoints are slightly blurred so that the mass concentration is visible. S4_2_Fig.png: The artificial data set BLK.4_0.2_0.5—adjacency matrix for documents S4_3_Fig.png: S4_3_FigButtom.png: Mass of the element with the largest mass in the eigenvector. Eigenvectors are ordered by decreasing eigenvalue. Top figure: the entire spectrum. Bottom figure: only the 50 eigenvectors corresponding to 50 lowest eigenvalues. English Twitter data TWT.EN. S4_4_Fig.png: S4_4_FigButtom.png: Number of highest mass elements of eigenvectors constituting half mass of the eigenvector. English Twitter data TWT.EN. Top: all eigenvectors. Bottom: 50 eigenvectors with the lowest eigenvalue. S4_5_Fig.png: S4_5_FigButtom.png: Relative error among the elements of eigenvector constituting its halfmass (standard error divided by the mean). English Twitter data TWT.EN. Top: all eigenvectors. Bottom: 50 eigenvectors with the lowest eigenvalue. S4_6_Fig.png: S4_6_FigButtom.png: Eigenvalue distributions for the entire dataset (the black dots) and for the classes (lines with different colors) for combinatorial (top) and normalized (bottom) graph Laplacian. English Twitter data TWT.EN. On the bottom, ten lowest eigenvalues were omitted for better readability. S4_7_Fig.png: Combinatorial Laplacian of the entire TWT.PL data set (thick line) and of each of the classes. S4_8_Fig.png: Combinatorial Laplacian spectrum of the class #pizgaczhell of TWT.PL data set and of samples of size 25%, 50% and 75%. S4_9_Fig.png: Combinatorial Laplacian spectrum of the class #pizgaczhell of TWT.PL data set and several samples of size 50%. S4_10_Fig.png: Combinatorial Laplacian spectrum of the class #pizgaczhell o [file pone.0283413.s004.zip › S4_3_Fig.png]

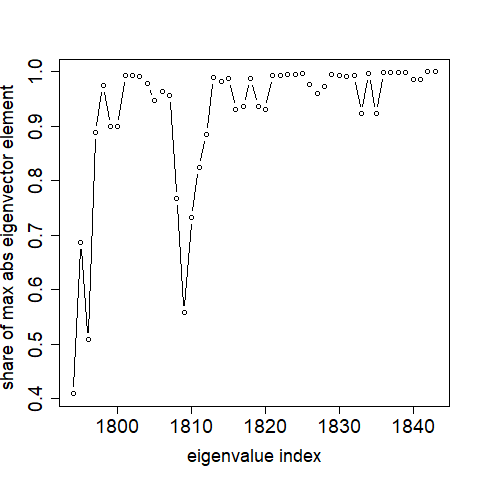

Supplement: S4 File — S4_1_Fig.png: Distribution of objects in the space spanned by the eigenvectors of combinatorial Laplacian corresponding to some of the lowest eigenvalues (no. 1490 and 1488)—TWT.PL dataset: in two corners there are two objects, while the rest is located in the third corner (mass concentration). The positions of datapoints are slightly blurred so that the mass concentration is visible. S4_2_Fig.png: The artificial data set BLK.4_0.2_0.5—adjacency matrix for documents S4_3_Fig.png: S4_3_FigButtom.png: Mass of the element with the largest mass in the eigenvector. Eigenvectors are ordered by decreasing eigenvalue. Top figure: the entire spectrum. Bottom figure: only the 50 eigenvectors corresponding to 50 lowest eigenvalues. English Twitter data TWT.EN. S4_4_Fig.png: S4_4_FigButtom.png: Number of highest mass elements of eigenvectors constituting half mass of the eigenvector. English Twitter data TWT.EN. Top: all eigenvectors. Bottom: 50 eigenvectors with the lowest eigenvalue. S4_5_Fig.png: S4_5_FigButtom.png: Relative error among the elements of eigenvector constituting its halfmass (standard error divided by the mean). English Twitter data TWT.EN. Top: all eigenvectors. Bottom: 50 eigenvectors with the lowest eigenvalue. S4_6_Fig.png: S4_6_FigButtom.png: Eigenvalue distributions for the entire dataset (the black dots) and for the classes (lines with different colors) for combinatorial (top) and normalized (bottom) graph Laplacian. English Twitter data TWT.EN. On the bottom, ten lowest eigenvalues were omitted for better readability. S4_7_Fig.png: Combinatorial Laplacian of the entire TWT.PL data set (thick line) and of each of the classes. S4_8_Fig.png: Combinatorial Laplacian spectrum of the class #pizgaczhell of TWT.PL data set and of samples of size 25%, 50% and 75%. S4_9_Fig.png: Combinatorial Laplacian spectrum of the class #pizgaczhell of TWT.PL data set and several samples of size 50%. S4_10_Fig.png: Combinatorial Laplacian spectrum of the class #pizgaczhell o [file pone.0283413.s004.zip › S4_3_FigBottom.png]

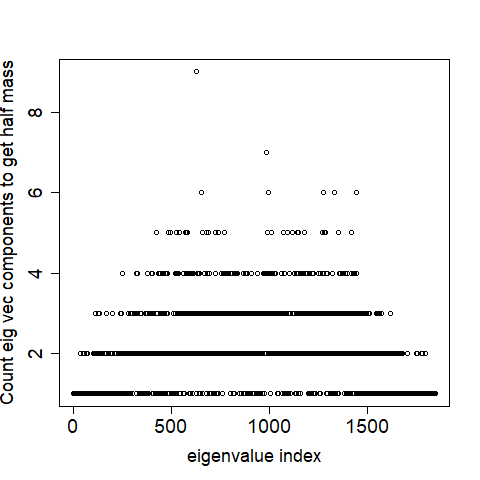

Supplement: S4 File — S4_1_Fig.png: Distribution of objects in the space spanned by the eigenvectors of combinatorial Laplacian corresponding to some of the lowest eigenvalues (no. 1490 and 1488)—TWT.PL dataset: in two corners there are two objects, while the rest is located in the third corner (mass concentration). The positions of datapoints are slightly blurred so that the mass concentration is visible. S4_2_Fig.png: The artificial data set BLK.4_0.2_0.5—adjacency matrix for documents S4_3_Fig.png: S4_3_FigButtom.png: Mass of the element with the largest mass in the eigenvector. Eigenvectors are ordered by decreasing eigenvalue. Top figure: the entire spectrum. Bottom figure: only the 50 eigenvectors corresponding to 50 lowest eigenvalues. English Twitter data TWT.EN. S4_4_Fig.png: S4_4_FigButtom.png: Number of highest mass elements of eigenvectors constituting half mass of the eigenvector. English Twitter data TWT.EN. Top: all eigenvectors. Bottom: 50 eigenvectors with the lowest eigenvalue. S4_5_Fig.png: S4_5_FigButtom.png: Relative error among the elements of eigenvector constituting its halfmass (standard error divided by the mean). English Twitter data TWT.EN. Top: all eigenvectors. Bottom: 50 eigenvectors with the lowest eigenvalue. S4_6_Fig.png: S4_6_FigButtom.png: Eigenvalue distributions for the entire dataset (the black dots) and for the classes (lines with different colors) for combinatorial (top) and normalized (bottom) graph Laplacian. English Twitter data TWT.EN. On the bottom, ten lowest eigenvalues were omitted for better readability. S4_7_Fig.png: Combinatorial Laplacian of the entire TWT.PL data set (thick line) and of each of the classes. S4_8_Fig.png: Combinatorial Laplacian spectrum of the class #pizgaczhell of TWT.PL data set and of samples of size 25%, 50% and 75%. S4_9_Fig.png: Combinatorial Laplacian spectrum of the class #pizgaczhell of TWT.PL data set and several samples of size 50%. S4_10_Fig.png: Combinatorial Laplacian spectrum of the class #pizgaczhell o [file pone.0283413.s004.zip › S4_4_Fig.png]

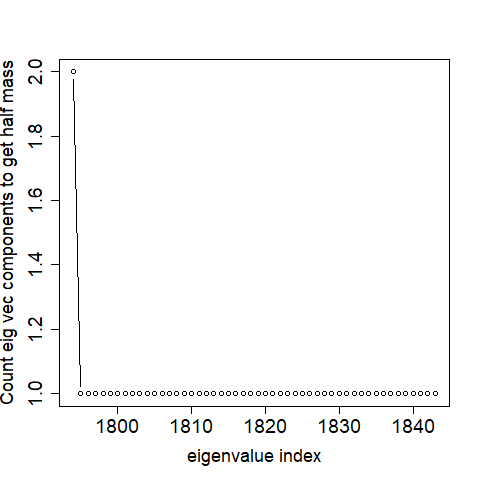

Supplement: S4 File — S4_1_Fig.png: Distribution of objects in the space spanned by the eigenvectors of combinatorial Laplacian corresponding to some of the lowest eigenvalues (no. 1490 and 1488)—TWT.PL dataset: in two corners there are two objects, while the rest is located in the third corner (mass concentration). The positions of datapoints are slightly blurred so that the mass concentration is visible. S4_2_Fig.png: The artificial data set BLK.4_0.2_0.5—adjacency matrix for documents S4_3_Fig.png: S4_3_FigButtom.png: Mass of the element with the largest mass in the eigenvector. Eigenvectors are ordered by decreasing eigenvalue. Top figure: the entire spectrum. Bottom figure: only the 50 eigenvectors corresponding to 50 lowest eigenvalues. English Twitter data TWT.EN. S4_4_Fig.png: S4_4_FigButtom.png: Number of highest mass elements of eigenvectors constituting half mass of the eigenvector. English Twitter data TWT.EN. Top: all eigenvectors. Bottom: 50 eigenvectors with the lowest eigenvalue. S4_5_Fig.png: S4_5_FigButtom.png: Relative error among the elements of eigenvector constituting its halfmass (standard error divided by the mean). English Twitter data TWT.EN. Top: all eigenvectors. Bottom: 50 eigenvectors with the lowest eigenvalue. S4_6_Fig.png: S4_6_FigButtom.png: Eigenvalue distributions for the entire dataset (the black dots) and for the classes (lines with different colors) for combinatorial (top) and normalized (bottom) graph Laplacian. English Twitter data TWT.EN. On the bottom, ten lowest eigenvalues were omitted for better readability. S4_7_Fig.png: Combinatorial Laplacian of the entire TWT.PL data set (thick line) and of each of the classes. S4_8_Fig.png: Combinatorial Laplacian spectrum of the class #pizgaczhell of TWT.PL data set and of samples of size 25%, 50% and 75%. S4_9_Fig.png: Combinatorial Laplacian spectrum of the class #pizgaczhell of TWT.PL data set and several samples of size 50%. S4_10_Fig.png: Combinatorial Laplacian spectrum of the class #pizgaczhell o [file pone.0283413.s004.zip › S4_4_FigBottom.png]

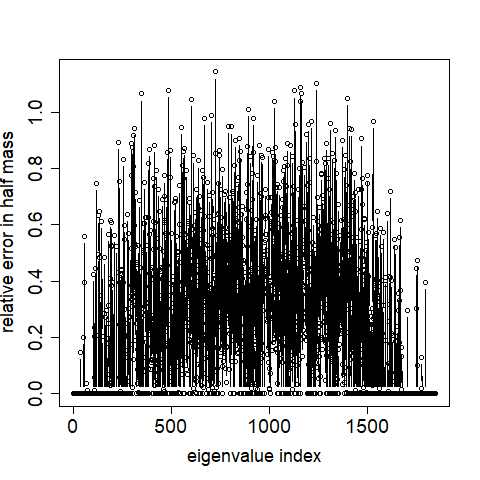

Supplement: S4 File — S4_1_Fig.png: Distribution of objects in the space spanned by the eigenvectors of combinatorial Laplacian corresponding to some of the lowest eigenvalues (no. 1490 and 1488)—TWT.PL dataset: in two corners there are two objects, while the rest is located in the third corner (mass concentration). The positions of datapoints are slightly blurred so that the mass concentration is visible. S4_2_Fig.png: The artificial data set BLK.4_0.2_0.5—adjacency matrix for documents S4_3_Fig.png: S4_3_FigButtom.png: Mass of the element with the largest mass in the eigenvector. Eigenvectors are ordered by decreasing eigenvalue. Top figure: the entire spectrum. Bottom figure: only the 50 eigenvectors corresponding to 50 lowest eigenvalues. English Twitter data TWT.EN. S4_4_Fig.png: S4_4_FigButtom.png: Number of highest mass elements of eigenvectors constituting half mass of the eigenvector. English Twitter data TWT.EN. Top: all eigenvectors. Bottom: 50 eigenvectors with the lowest eigenvalue. S4_5_Fig.png: S4_5_FigButtom.png: Relative error among the elements of eigenvector constituting its halfmass (standard error divided by the mean). English Twitter data TWT.EN. Top: all eigenvectors. Bottom: 50 eigenvectors with the lowest eigenvalue. S4_6_Fig.png: S4_6_FigButtom.png: Eigenvalue distributions for the entire dataset (the black dots) and for the classes (lines with different colors) for combinatorial (top) and normalized (bottom) graph Laplacian. English Twitter data TWT.EN. On the bottom, ten lowest eigenvalues were omitted for better readability. S4_7_Fig.png: Combinatorial Laplacian of the entire TWT.PL data set (thick line) and of each of the classes. S4_8_Fig.png: Combinatorial Laplacian spectrum of the class #pizgaczhell of TWT.PL data set and of samples of size 25%, 50% and 75%. S4_9_Fig.png: Combinatorial Laplacian spectrum of the class #pizgaczhell of TWT.PL data set and several samples of size 50%. S4_10_Fig.png: Combinatorial Laplacian spectrum of the class #pizgaczhell o [file pone.0283413.s004.zip › S4_5_Fig.png]

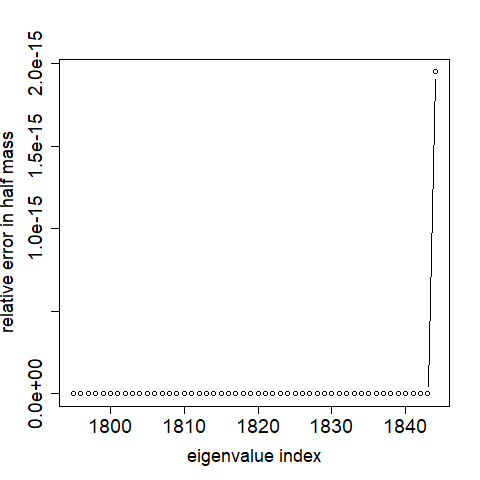

Supplement: S4 File — S4_1_Fig.png: Distribution of objects in the space spanned by the eigenvectors of combinatorial Laplacian corresponding to some of the lowest eigenvalues (no. 1490 and 1488)—TWT.PL dataset: in two corners there are two objects, while the rest is located in the third corner (mass concentration). The positions of datapoints are slightly blurred so that the mass concentration is visible. S4_2_Fig.png: The artificial data set BLK.4_0.2_0.5—adjacency matrix for documents S4_3_Fig.png: S4_3_FigButtom.png: Mass of the element with the largest mass in the eigenvector. Eigenvectors are ordered by decreasing eigenvalue. Top figure: the entire spectrum. Bottom figure: only the 50 eigenvectors corresponding to 50 lowest eigenvalues. English Twitter data TWT.EN. S4_4_Fig.png: S4_4_FigButtom.png: Number of highest mass elements of eigenvectors constituting half mass of the eigenvector. English Twitter data TWT.EN. Top: all eigenvectors. Bottom: 50 eigenvectors with the lowest eigenvalue. S4_5_Fig.png: S4_5_FigButtom.png: Relative error among the elements of eigenvector constituting its halfmass (standard error divided by the mean). English Twitter data TWT.EN. Top: all eigenvectors. Bottom: 50 eigenvectors with the lowest eigenvalue. S4_6_Fig.png: S4_6_FigButtom.png: Eigenvalue distributions for the entire dataset (the black dots) and for the classes (lines with different colors) for combinatorial (top) and normalized (bottom) graph Laplacian. English Twitter data TWT.EN. On the bottom, ten lowest eigenvalues were omitted for better readability. S4_7_Fig.png: Combinatorial Laplacian of the entire TWT.PL data set (thick line) and of each of the classes. S4_8_Fig.png: Combinatorial Laplacian spectrum of the class #pizgaczhell of TWT.PL data set and of samples of size 25%, 50% and 75%. S4_9_Fig.png: Combinatorial Laplacian spectrum of the class #pizgaczhell of TWT.PL data set and several samples of size 50%. S4_10_Fig.png: Combinatorial Laplacian spectrum of the class #pizgaczhell o [file pone.0283413.s004.zip › S4_5_FigBottom.png]

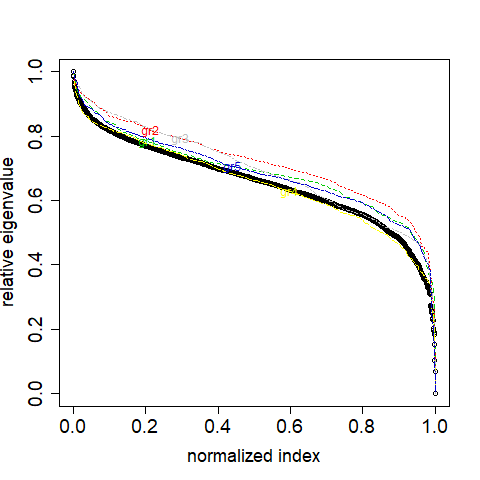

Supplement: S4 File — S4_1_Fig.png: Distribution of objects in the space spanned by the eigenvectors of combinatorial Laplacian corresponding to some of the lowest eigenvalues (no. 1490 and 1488)—TWT.PL dataset: in two corners there are two objects, while the rest is located in the third corner (mass concentration). The positions of datapoints are slightly blurred so that the mass concentration is visible. S4_2_Fig.png: The artificial data set BLK.4_0.2_0.5—adjacency matrix for documents S4_3_Fig.png: S4_3_FigButtom.png: Mass of the element with the largest mass in the eigenvector. Eigenvectors are ordered by decreasing eigenvalue. Top figure: the entire spectrum. Bottom figure: only the 50 eigenvectors corresponding to 50 lowest eigenvalues. English Twitter data TWT.EN. S4_4_Fig.png: S4_4_FigButtom.png: Number of highest mass elements of eigenvectors constituting half mass of the eigenvector. English Twitter data TWT.EN. Top: all eigenvectors. Bottom: 50 eigenvectors with the lowest eigenvalue. S4_5_Fig.png: S4_5_FigButtom.png: Relative error among the elements of eigenvector constituting its halfmass (standard error divided by the mean). English Twitter data TWT.EN. Top: all eigenvectors. Bottom: 50 eigenvectors with the lowest eigenvalue. S4_6_Fig.png: S4_6_FigButtom.png: Eigenvalue distributions for the entire dataset (the black dots) and for the classes (lines with different colors) for combinatorial (top) and normalized (bottom) graph Laplacian. English Twitter data TWT.EN. On the bottom, ten lowest eigenvalues were omitted for better readability. S4_7_Fig.png: Combinatorial Laplacian of the entire TWT.PL data set (thick line) and of each of the classes. S4_8_Fig.png: Combinatorial Laplacian spectrum of the class #pizgaczhell of TWT.PL data set and of samples of size 25%, 50% and 75%. S4_9_Fig.png: Combinatorial Laplacian spectrum of the class #pizgaczhell of TWT.PL data set and several samples of size 50%. S4_10_Fig.png: Combinatorial Laplacian spectrum of the class #pizgaczhell o [file pone.0283413.s004.zip › S4_6_Fig.png]

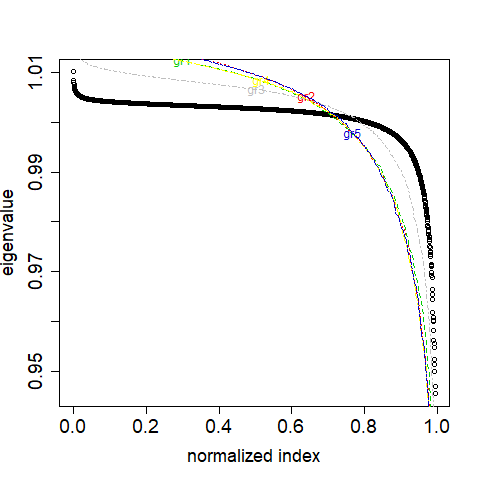

Supplement: S4 File — S4_1_Fig.png: Distribution of objects in the space spanned by the eigenvectors of combinatorial Laplacian corresponding to some of the lowest eigenvalues (no. 1490 and 1488)—TWT.PL dataset: in two corners there are two objects, while the rest is located in the third corner (mass concentration). The positions of datapoints are slightly blurred so that the mass concentration is visible. S4_2_Fig.png: The artificial data set BLK.4_0.2_0.5—adjacency matrix for documents S4_3_Fig.png: S4_3_FigButtom.png: Mass of the element with the largest mass in the eigenvector. Eigenvectors are ordered by decreasing eigenvalue. Top figure: the entire spectrum. Bottom figure: only the 50 eigenvectors corresponding to 50 lowest eigenvalues. English Twitter data TWT.EN. S4_4_Fig.png: S4_4_FigButtom.png: Number of highest mass elements of eigenvectors constituting half mass of the eigenvector. English Twitter data TWT.EN. Top: all eigenvectors. Bottom: 50 eigenvectors with the lowest eigenvalue. S4_5_Fig.png: S4_5_FigButtom.png: Relative error among the elements of eigenvector constituting its halfmass (standard error divided by the mean). English Twitter data TWT.EN. Top: all eigenvectors. Bottom: 50 eigenvectors with the lowest eigenvalue. S4_6_Fig.png: S4_6_FigButtom.png: Eigenvalue distributions for the entire dataset (the black dots) and for the classes (lines with different colors) for combinatorial (top) and normalized (bottom) graph Laplacian. English Twitter data TWT.EN. On the bottom, ten lowest eigenvalues were omitted for better readability. S4_7_Fig.png: Combinatorial Laplacian of the entire TWT.PL data set (thick line) and of each of the classes. S4_8_Fig.png: Combinatorial Laplacian spectrum of the class #pizgaczhell of TWT.PL data set and of samples of size 25%, 50% and 75%. S4_9_Fig.png: Combinatorial Laplacian spectrum of the class #pizgaczhell of TWT.PL data set and several samples of size 50%. S4_10_Fig.png: Combinatorial Laplacian spectrum of the class #pizgaczhell o [file pone.0283413.s004.zip › S4_6_FigBottom.png]

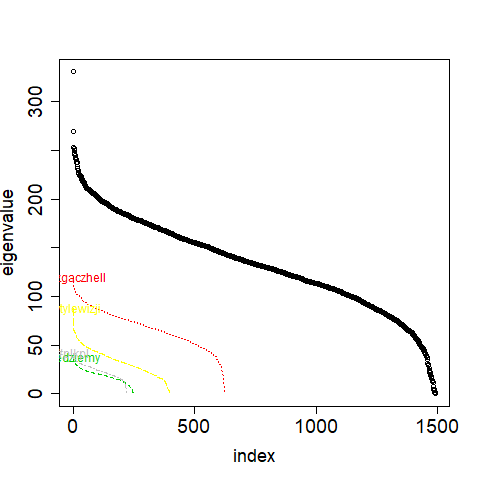

Supplement: S4 File — S4_1_Fig.png: Distribution of objects in the space spanned by the eigenvectors of combinatorial Laplacian corresponding to some of the lowest eigenvalues (no. 1490 and 1488)—TWT.PL dataset: in two corners there are two objects, while the rest is located in the third corner (mass concentration). The positions of datapoints are slightly blurred so that the mass concentration is visible. S4_2_Fig.png: The artificial data set BLK.4_0.2_0.5—adjacency matrix for documents S4_3_Fig.png: S4_3_FigButtom.png: Mass of the element with the largest mass in the eigenvector. Eigenvectors are ordered by decreasing eigenvalue. Top figure: the entire spectrum. Bottom figure: only the 50 eigenvectors corresponding to 50 lowest eigenvalues. English Twitter data TWT.EN. S4_4_Fig.png: S4_4_FigButtom.png: Number of highest mass elements of eigenvectors constituting half mass of the eigenvector. English Twitter data TWT.EN. Top: all eigenvectors. Bottom: 50 eigenvectors with the lowest eigenvalue. S4_5_Fig.png: S4_5_FigButtom.png: Relative error among the elements of eigenvector constituting its halfmass (standard error divided by the mean). English Twitter data TWT.EN. Top: all eigenvectors. Bottom: 50 eigenvectors with the lowest eigenvalue. S4_6_Fig.png: S4_6_FigButtom.png: Eigenvalue distributions for the entire dataset (the black dots) and for the classes (lines with different colors) for combinatorial (top) and normalized (bottom) graph Laplacian. English Twitter data TWT.EN. On the bottom, ten lowest eigenvalues were omitted for better readability. S4_7_Fig.png: Combinatorial Laplacian of the entire TWT.PL data set (thick line) and of each of the classes. S4_8_Fig.png: Combinatorial Laplacian spectrum of the class #pizgaczhell of TWT.PL data set and of samples of size 25%, 50% and 75%. S4_9_Fig.png: Combinatorial Laplacian spectrum of the class #pizgaczhell of TWT.PL data set and several samples of size 50%. S4_10_Fig.png: Combinatorial Laplacian spectrum of the class #pizgaczhell o [file pone.0283413.s004.zip › S4_7_Fig.png]

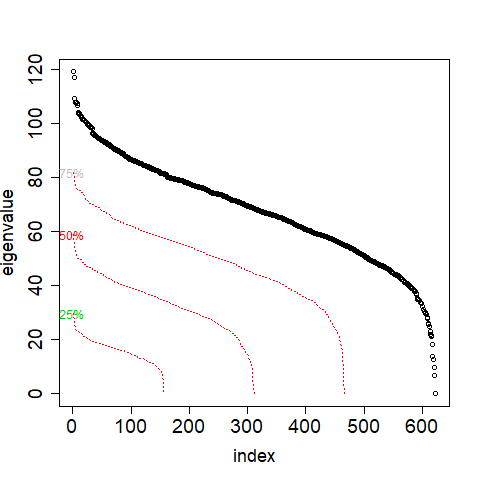

Supplement: S4 File — S4_1_Fig.png: Distribution of objects in the space spanned by the eigenvectors of combinatorial Laplacian corresponding to some of the lowest eigenvalues (no. 1490 and 1488)—TWT.PL dataset: in two corners there are two objects, while the rest is located in the third corner (mass concentration). The positions of datapoints are slightly blurred so that the mass concentration is visible. S4_2_Fig.png: The artificial data set BLK.4_0.2_0.5—adjacency matrix for documents S4_3_Fig.png: S4_3_FigButtom.png: Mass of the element with the largest mass in the eigenvector. Eigenvectors are ordered by decreasing eigenvalue. Top figure: the entire spectrum. Bottom figure: only the 50 eigenvectors corresponding to 50 lowest eigenvalues. English Twitter data TWT.EN. S4_4_Fig.png: S4_4_FigButtom.png: Number of highest mass elements of eigenvectors constituting half mass of the eigenvector. English Twitter data TWT.EN. Top: all eigenvectors. Bottom: 50 eigenvectors with the lowest eigenvalue. S4_5_Fig.png: S4_5_FigButtom.png: Relative error among the elements of eigenvector constituting its halfmass (standard error divided by the mean). English Twitter data TWT.EN. Top: all eigenvectors. Bottom: 50 eigenvectors with the lowest eigenvalue. S4_6_Fig.png: S4_6_FigButtom.png: Eigenvalue distributions for the entire dataset (the black dots) and for the classes (lines with different colors) for combinatorial (top) and normalized (bottom) graph Laplacian. English Twitter data TWT.EN. On the bottom, ten lowest eigenvalues were omitted for better readability. S4_7_Fig.png: Combinatorial Laplacian of the entire TWT.PL data set (thick line) and of each of the classes. S4_8_Fig.png: Combinatorial Laplacian spectrum of the class #pizgaczhell of TWT.PL data set and of samples of size 25%, 50% and 75%. S4_9_Fig.png: Combinatorial Laplacian spectrum of the class #pizgaczhell of TWT.PL data set and several samples of size 50%. S4_10_Fig.png: Combinatorial Laplacian spectrum of the class #pizgaczhell o [file pone.0283413.s004.zip › S4_8_Fig.png]

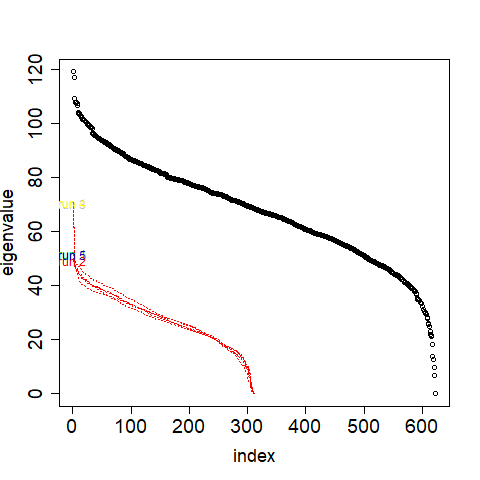

Supplement: S4 File — S4_1_Fig.png: Distribution of objects in the space spanned by the eigenvectors of combinatorial Laplacian corresponding to some of the lowest eigenvalues (no. 1490 and 1488)—TWT.PL dataset: in two corners there are two objects, while the rest is located in the third corner (mass concentration). The positions of datapoints are slightly blurred so that the mass concentration is visible. S4_2_Fig.png: The artificial data set BLK.4_0.2_0.5—adjacency matrix for documents S4_3_Fig.png: S4_3_FigButtom.png: Mass of the element with the largest mass in the eigenvector. Eigenvectors are ordered by decreasing eigenvalue. Top figure: the entire spectrum. Bottom figure: only the 50 eigenvectors corresponding to 50 lowest eigenvalues. English Twitter data TWT.EN. S4_4_Fig.png: S4_4_FigButtom.png: Number of highest mass elements of eigenvectors constituting half mass of the eigenvector. English Twitter data TWT.EN. Top: all eigenvectors. Bottom: 50 eigenvectors with the lowest eigenvalue. S4_5_Fig.png: S4_5_FigButtom.png: Relative error among the elements of eigenvector constituting its halfmass (standard error divided by the mean). English Twitter data TWT.EN. Top: all eigenvectors. Bottom: 50 eigenvectors with the lowest eigenvalue. S4_6_Fig.png: S4_6_FigButtom.png: Eigenvalue distributions for the entire dataset (the black dots) and for the classes (lines with different colors) for combinatorial (top) and normalized (bottom) graph Laplacian. English Twitter data TWT.EN. On the bottom, ten lowest eigenvalues were omitted for better readability. S4_7_Fig.png: Combinatorial Laplacian of the entire TWT.PL data set (thick line) and of each of the classes. S4_8_Fig.png: Combinatorial Laplacian spectrum of the class #pizgaczhell of TWT.PL data set and of samples of size 25%, 50% and 75%. S4_9_Fig.png: Combinatorial Laplacian spectrum of the class #pizgaczhell of TWT.PL data set and several samples of size 50%. S4_10_Fig.png: Combinatorial Laplacian spectrum of the class #pizgaczhell o [file pone.0283413.s004.zip › S4_9_Fig.png]

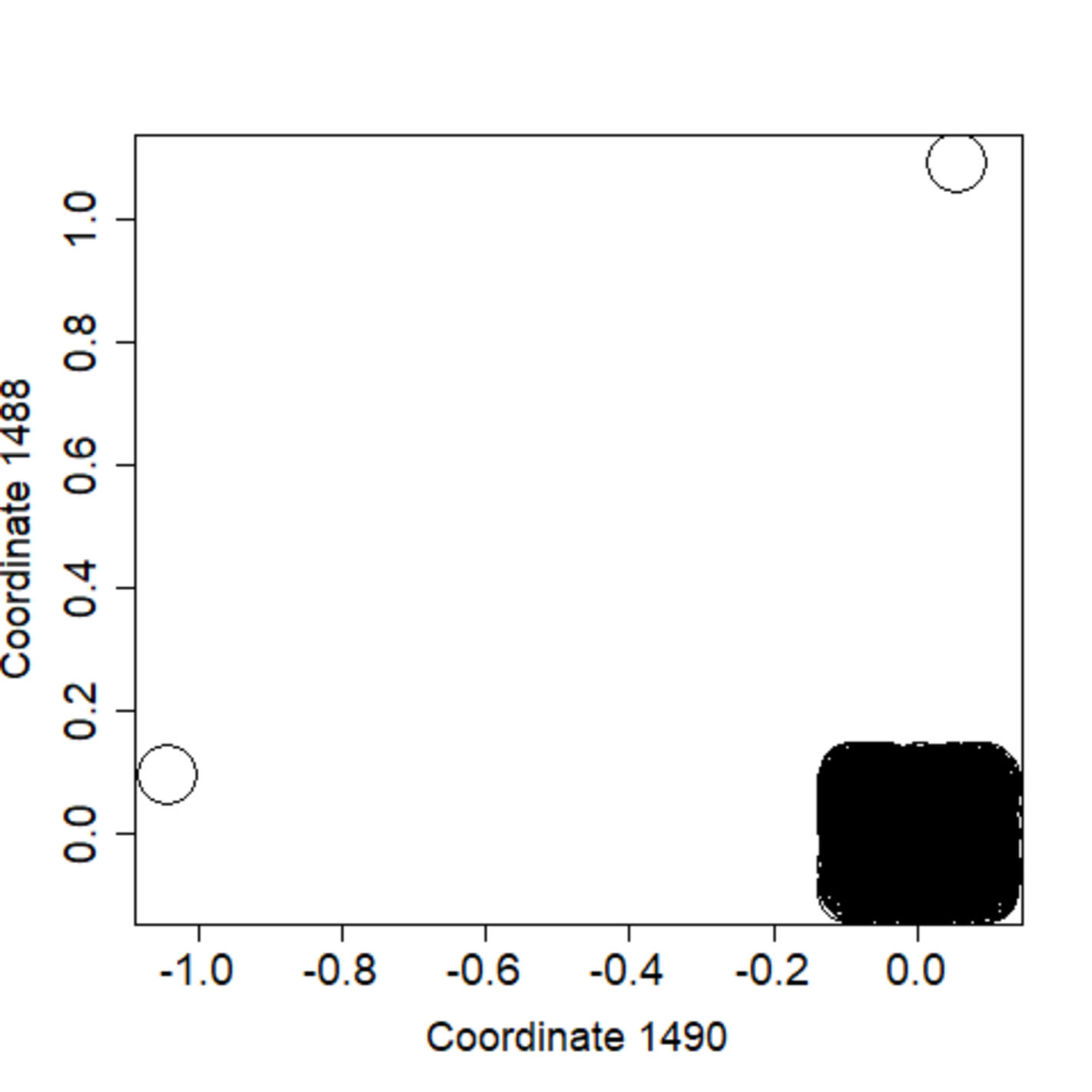

Supplement: S5 File — Names and captions are exactly the same as in S4 File, except that the extension is now TIFF. (ZIP) [file pone.0283413.s005.zip › S4_1_Fig.tiff]

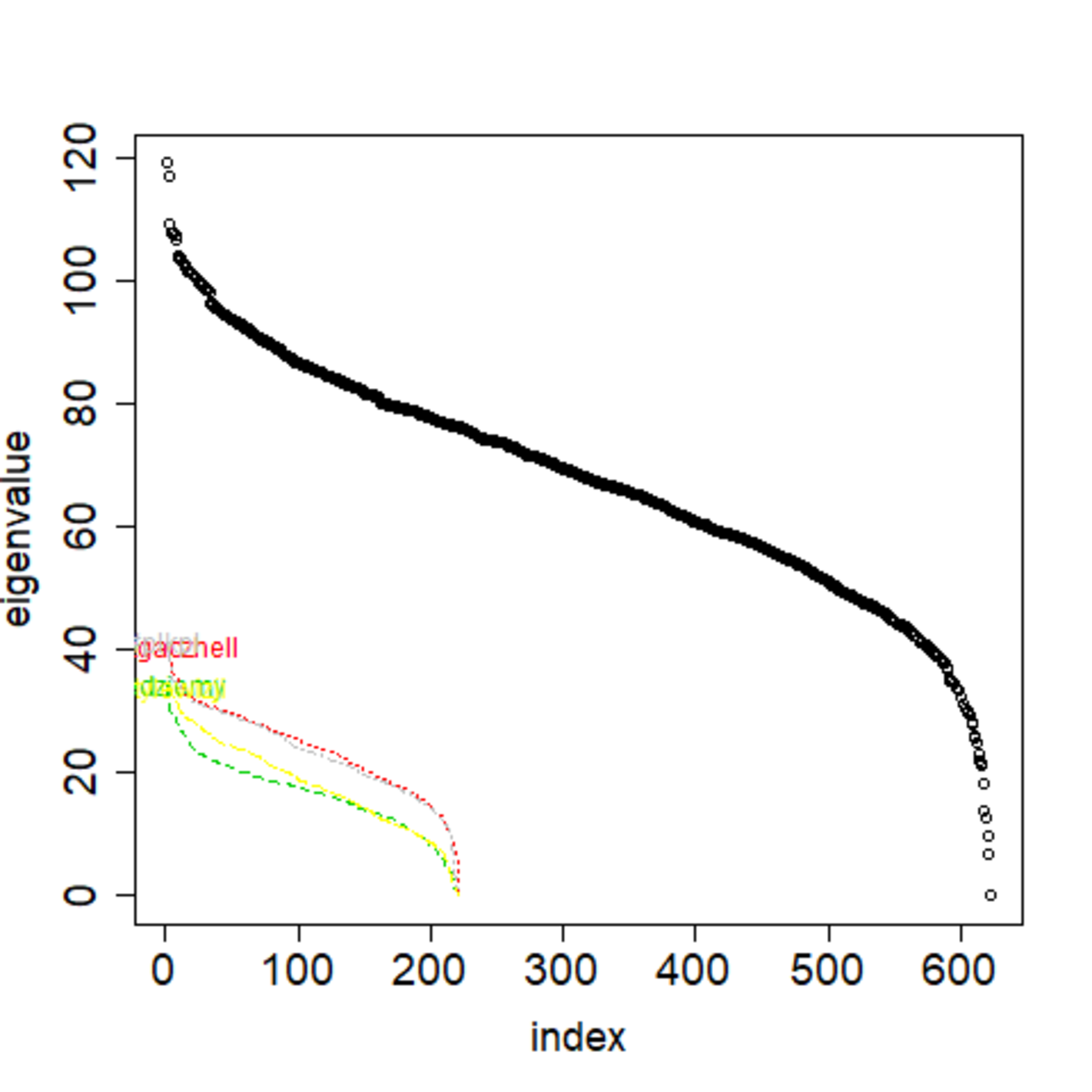

Supplement: S5 File — Names and captions are exactly the same as in S4 File, except that the extension is now TIFF. (ZIP) [file pone.0283413.s005.zip › S4_10_Fig.tiff]

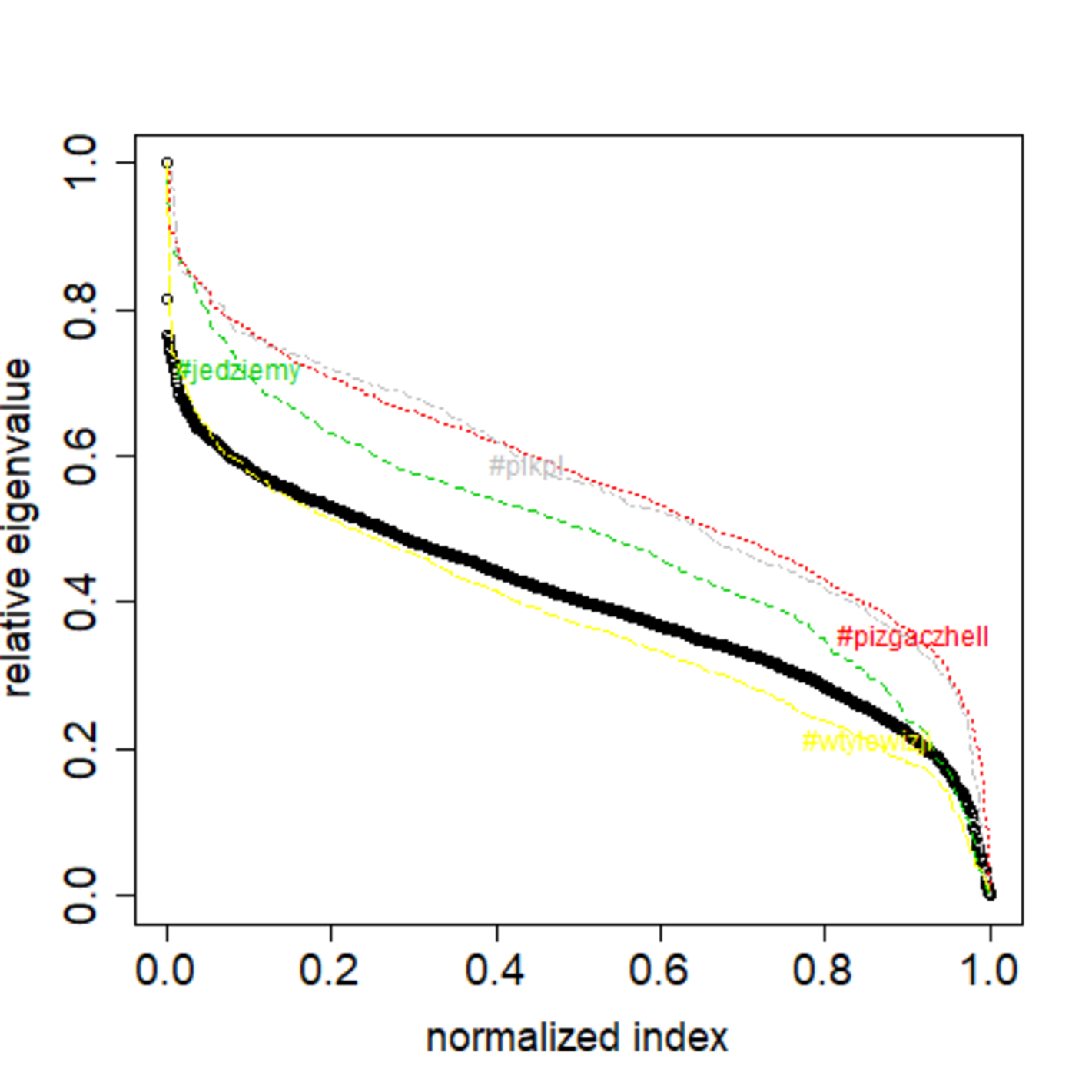

Supplement: S5 File — Names and captions are exactly the same as in S4 File, except that the extension is now TIFF. (ZIP) [file pone.0283413.s005.zip › S4_11_Fig.tiff]

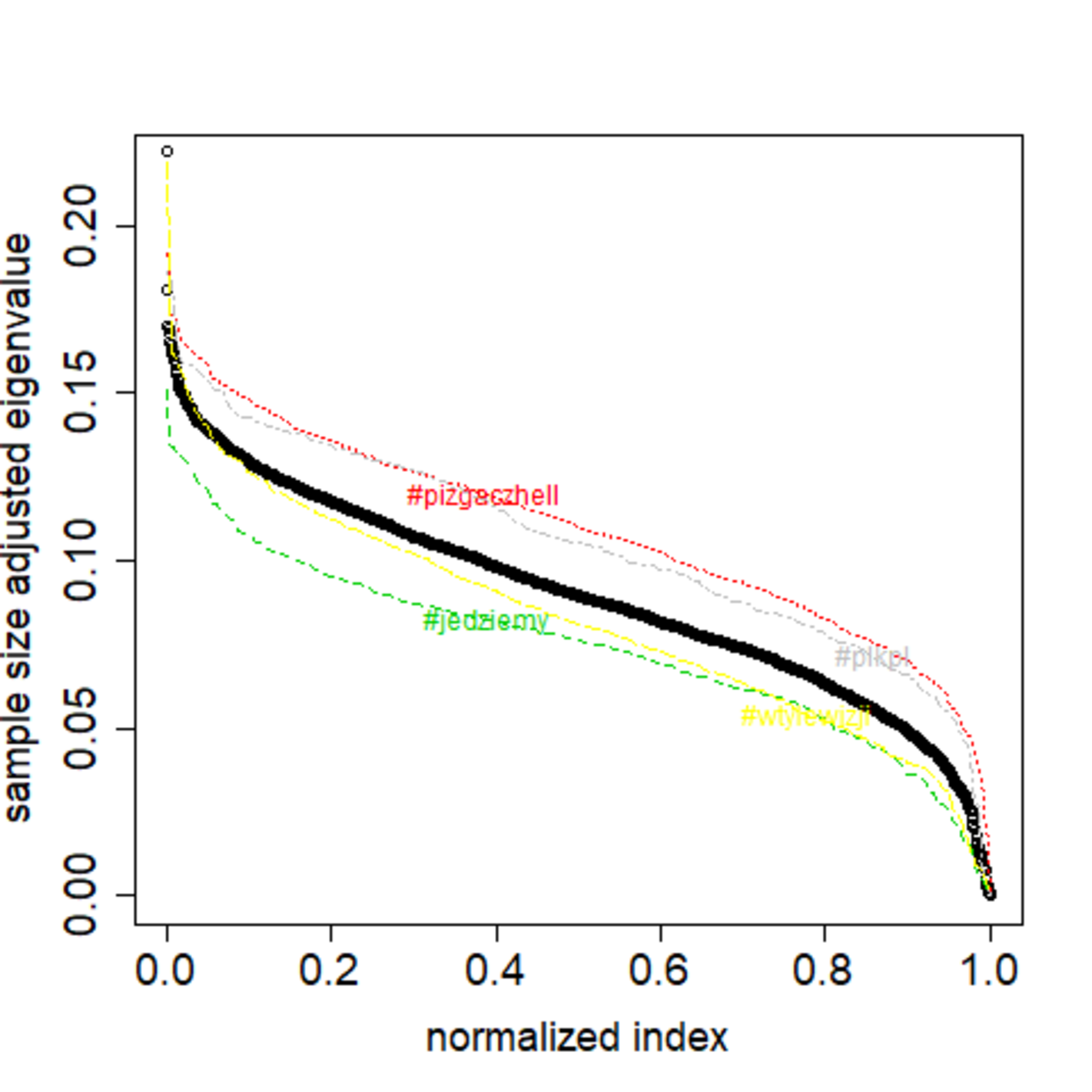

Supplement: S5 File — Names and captions are exactly the same as in S4 File, except that the extension is now TIFF. (ZIP) [file pone.0283413.s005.zip › S4_12_Fig.tiff]

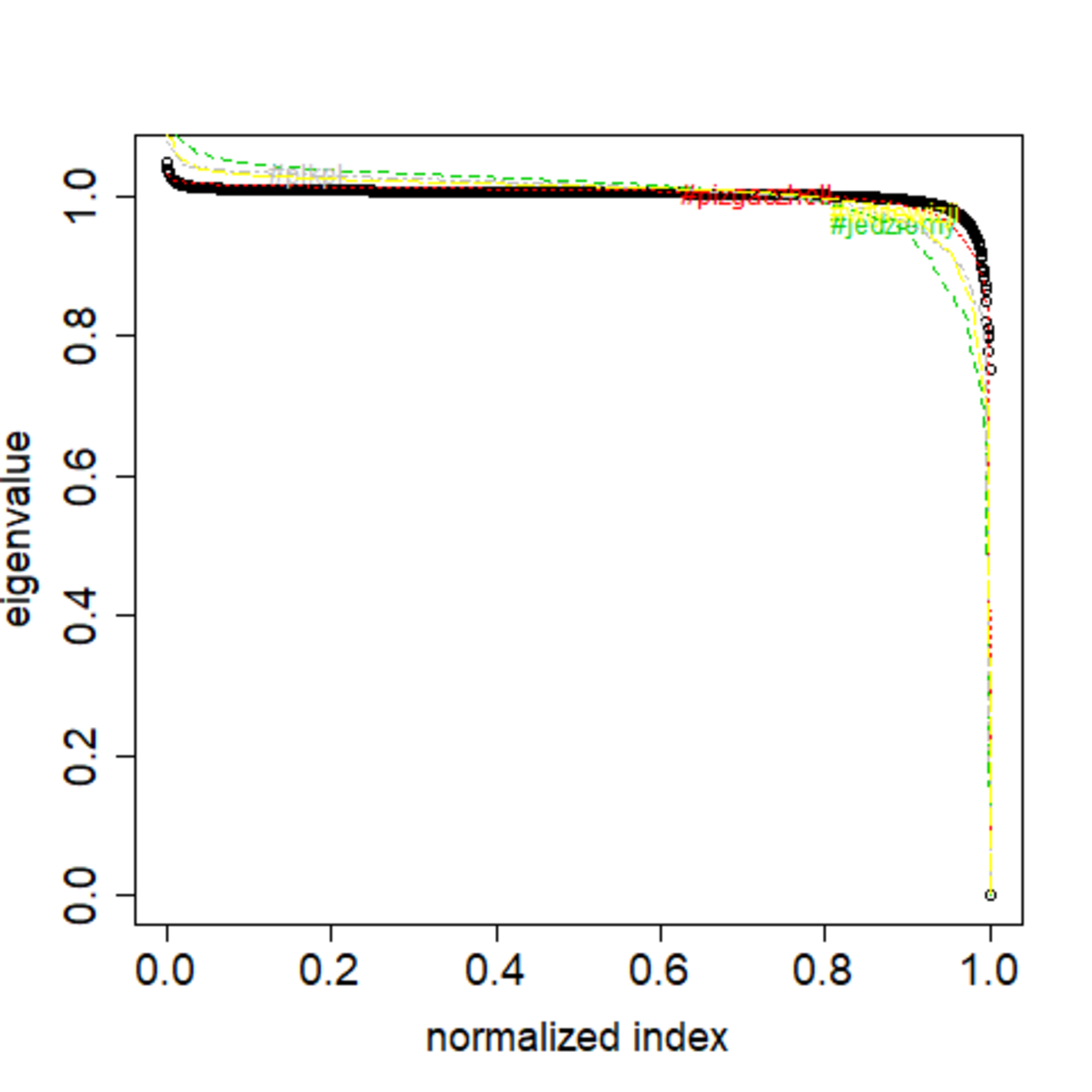

Supplement: S5 File — Names and captions are exactly the same as in S4 File, except that the extension is now TIFF. (ZIP) [file pone.0283413.s005.zip › S4_13_Fig.tiff]

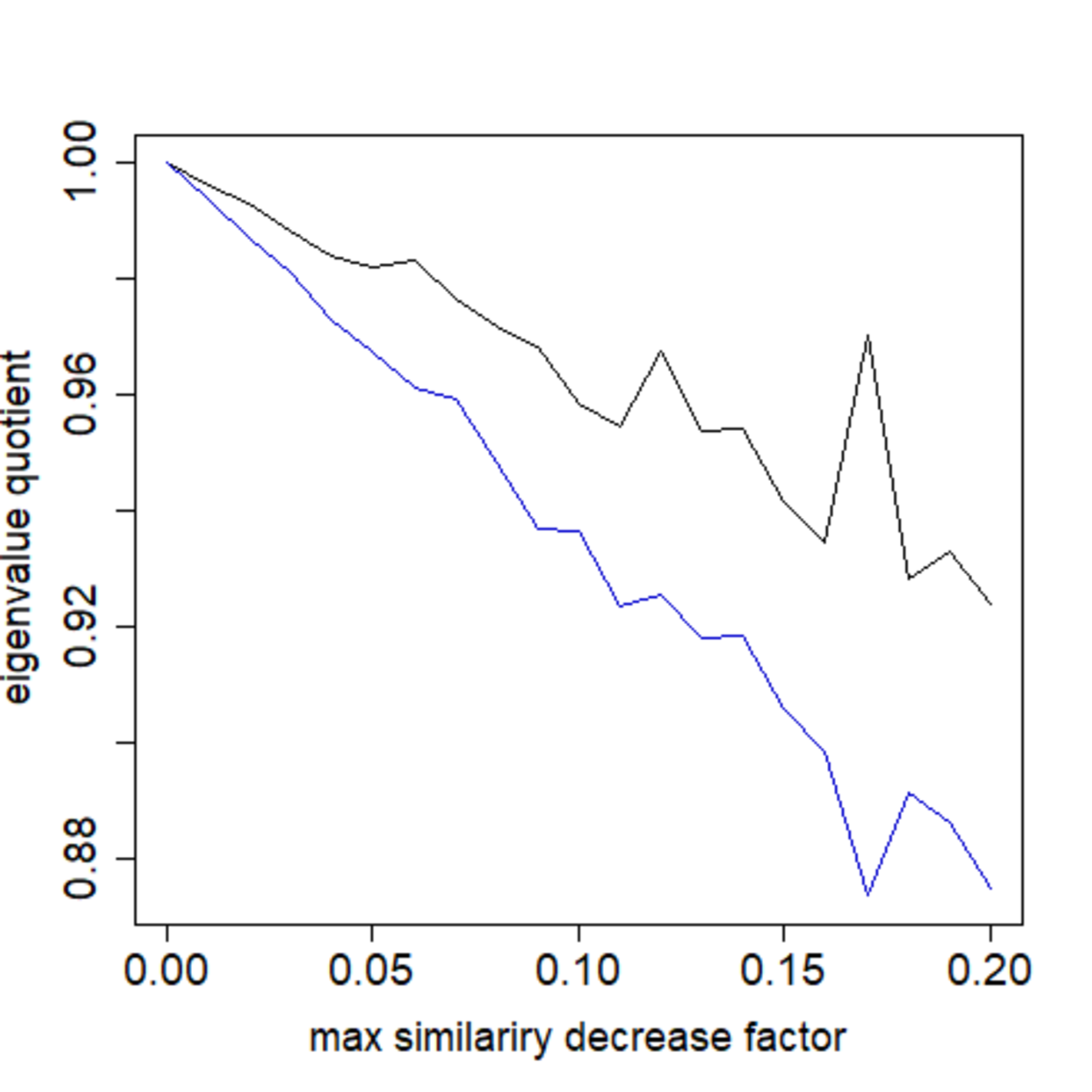

Supplement: S5 File — Names and captions are exactly the same as in S4 File, except that the extension is now TIFF. (ZIP) [file pone.0283413.s005.zip › S4_14_Fig.tiff]

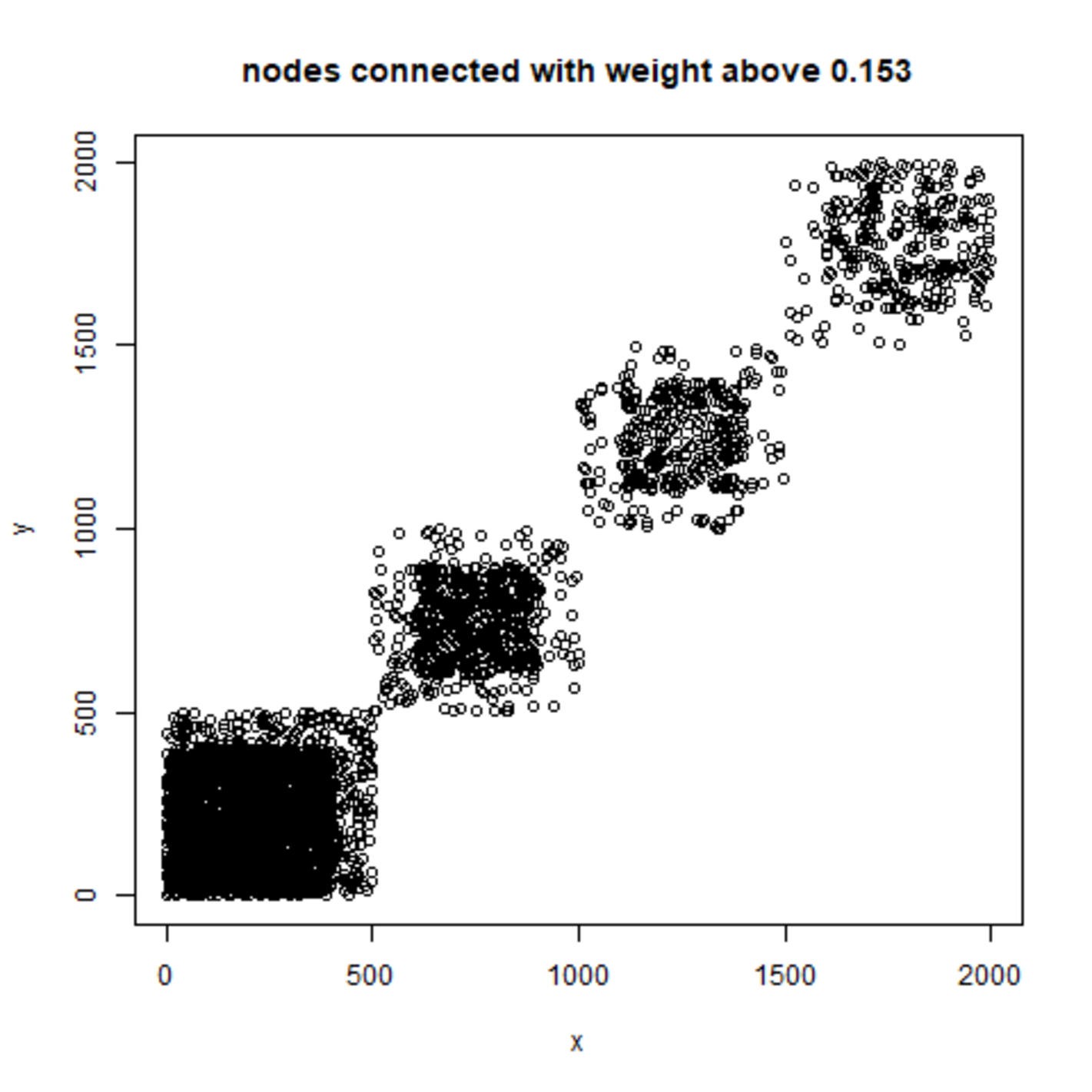

Supplement: S5 File — Names and captions are exactly the same as in S4 File, except that the extension is now TIFF. (ZIP) [file pone.0283413.s005.zip › S4_2_Fig.tiff]

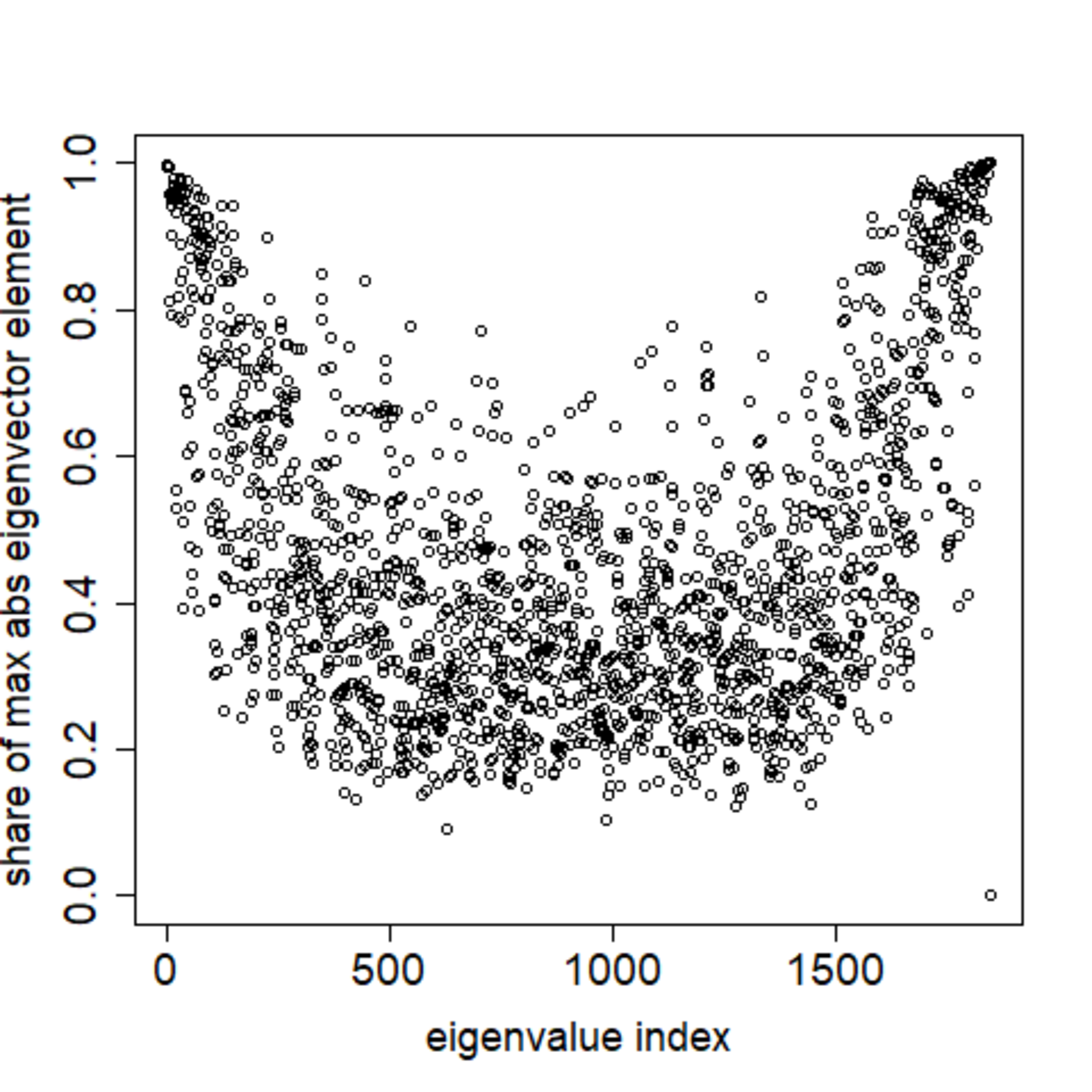

Supplement: S5 File — Names and captions are exactly the same as in S4 File, except that the extension is now TIFF. (ZIP) [file pone.0283413.s005.zip › S4_3_Fig.tiff]

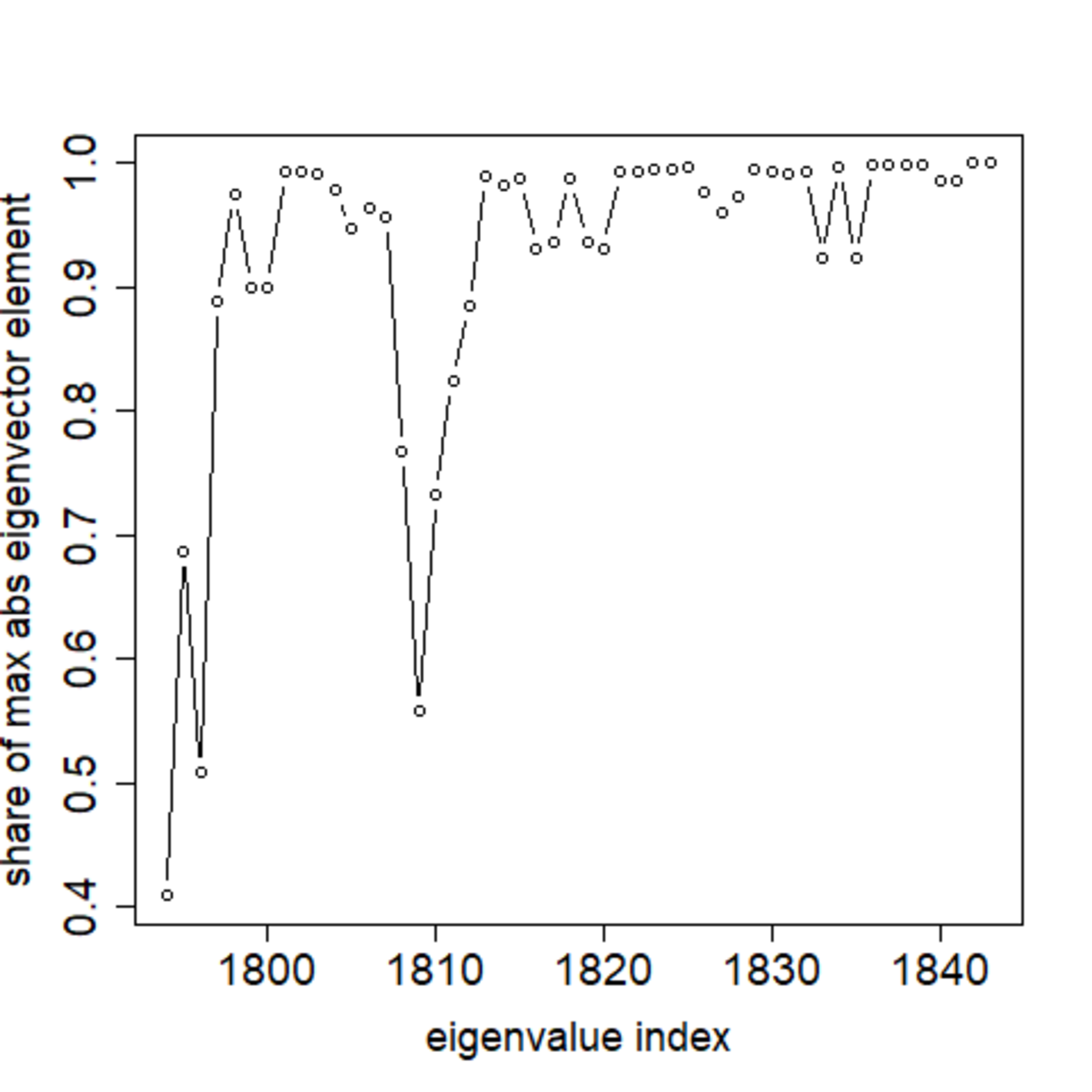

Supplement: S5 File — Names and captions are exactly the same as in S4 File, except that the extension is now TIFF. (ZIP) [file pone.0283413.s005.zip › S4_3_FigBottom.tiff]

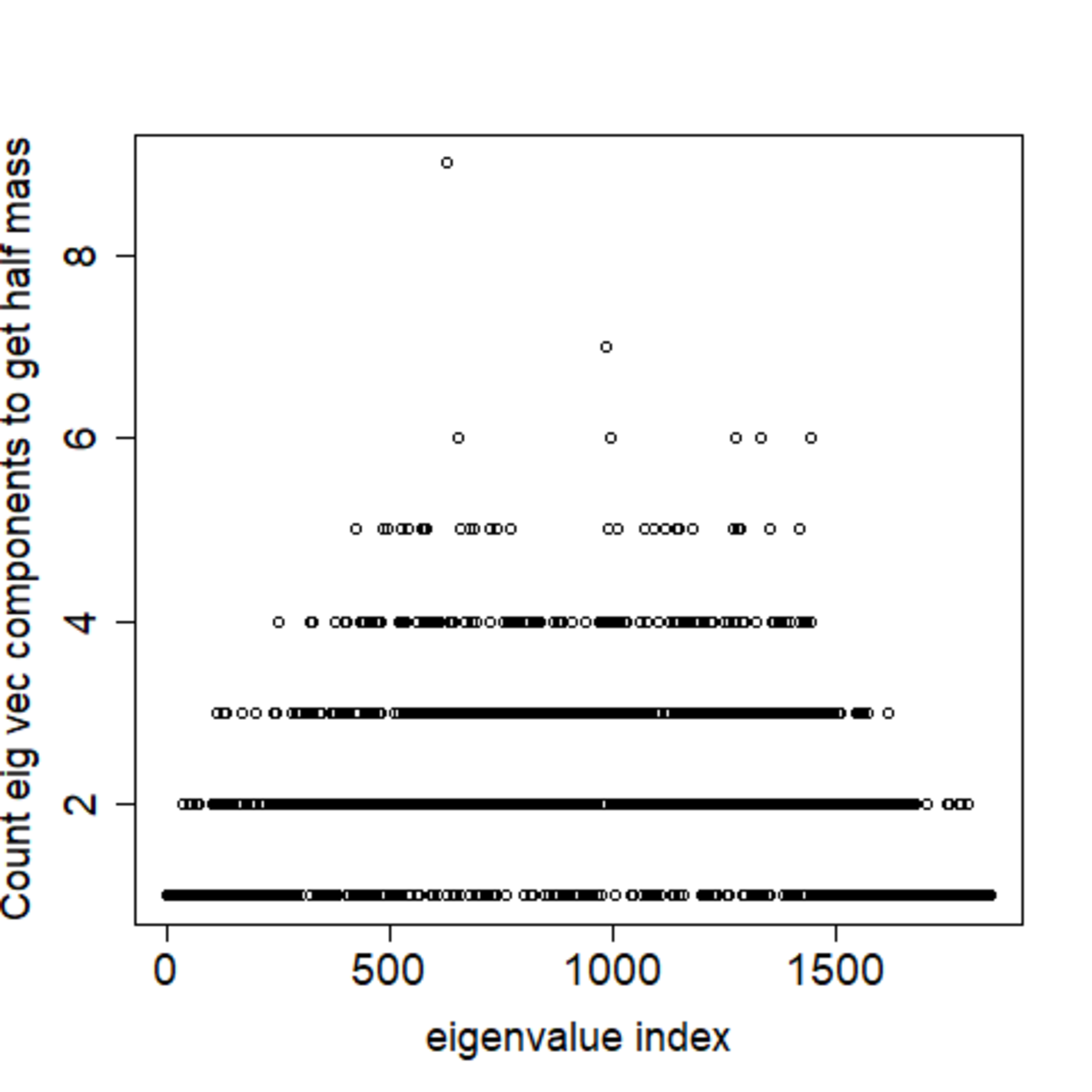

Supplement: S5 File — Names and captions are exactly the same as in S4 File, except that the extension is now TIFF. (ZIP) [file pone.0283413.s005.zip › S4_4_Fig.tiff]

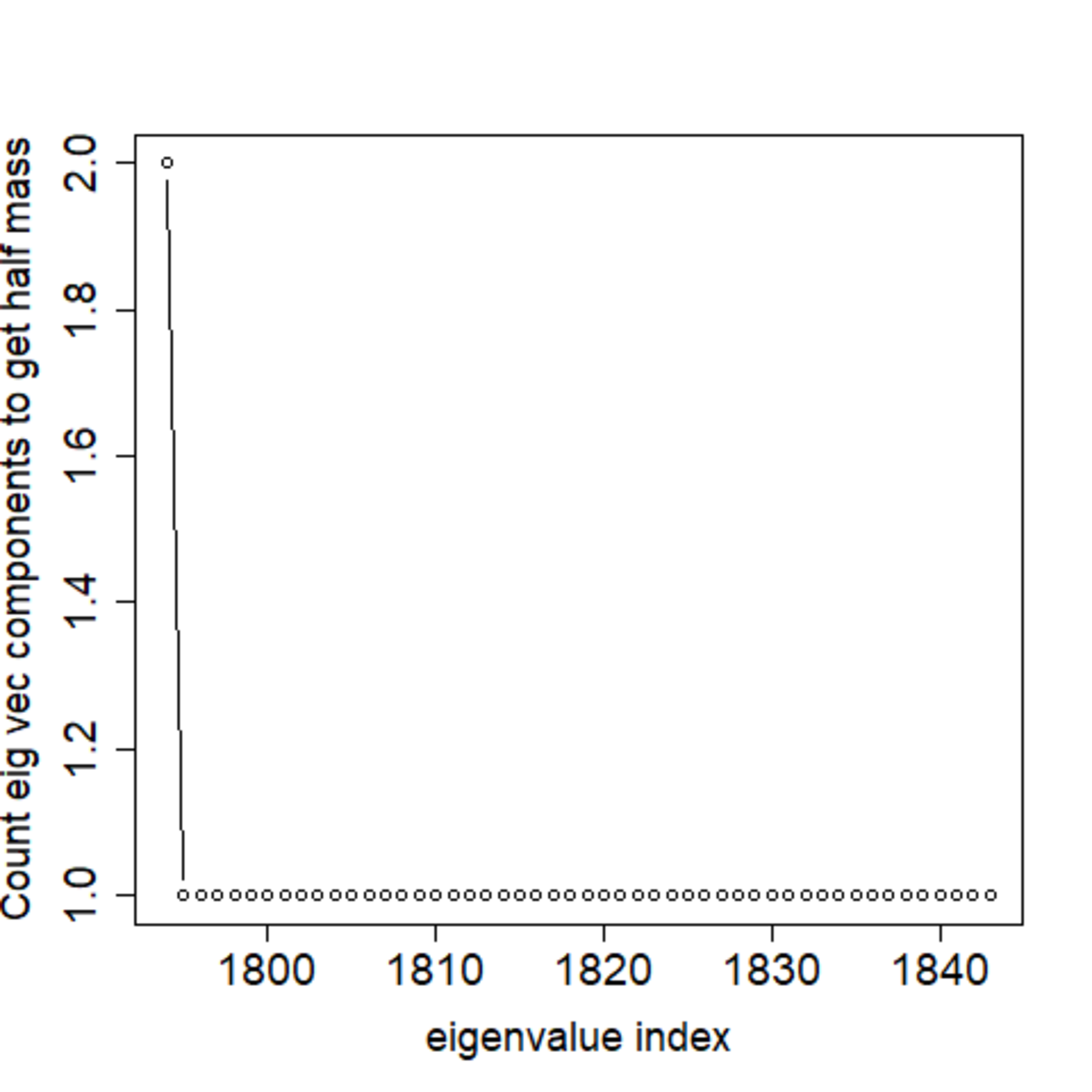

Supplement: S5 File — Names and captions are exactly the same as in S4 File, except that the extension is now TIFF. (ZIP) [file pone.0283413.s005.zip › S4_4_FigBottom.tiff]

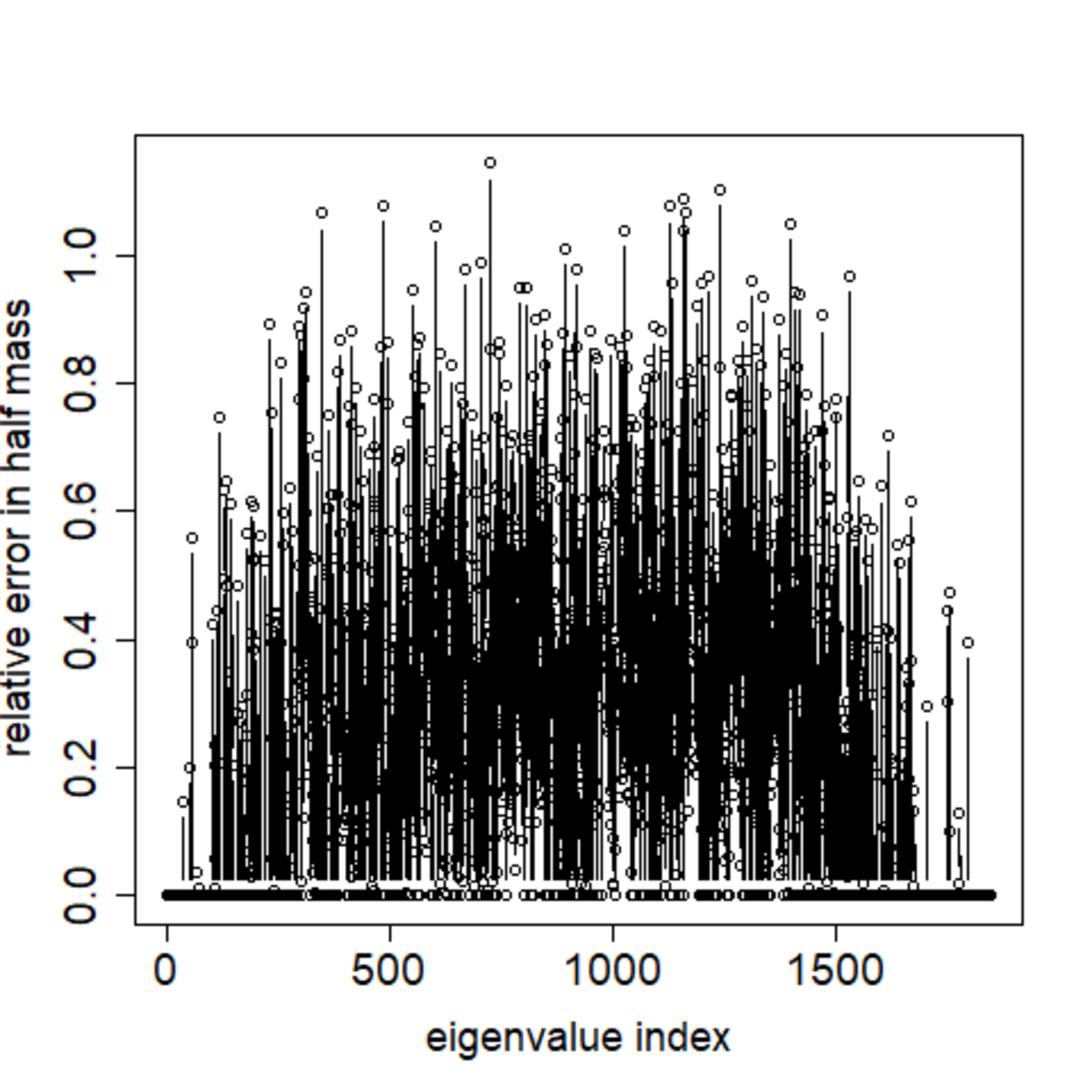

Supplement: S5 File — Names and captions are exactly the same as in S4 File, except that the extension is now TIFF. (ZIP) [file pone.0283413.s005.zip › S4_5_Fig.tiff]

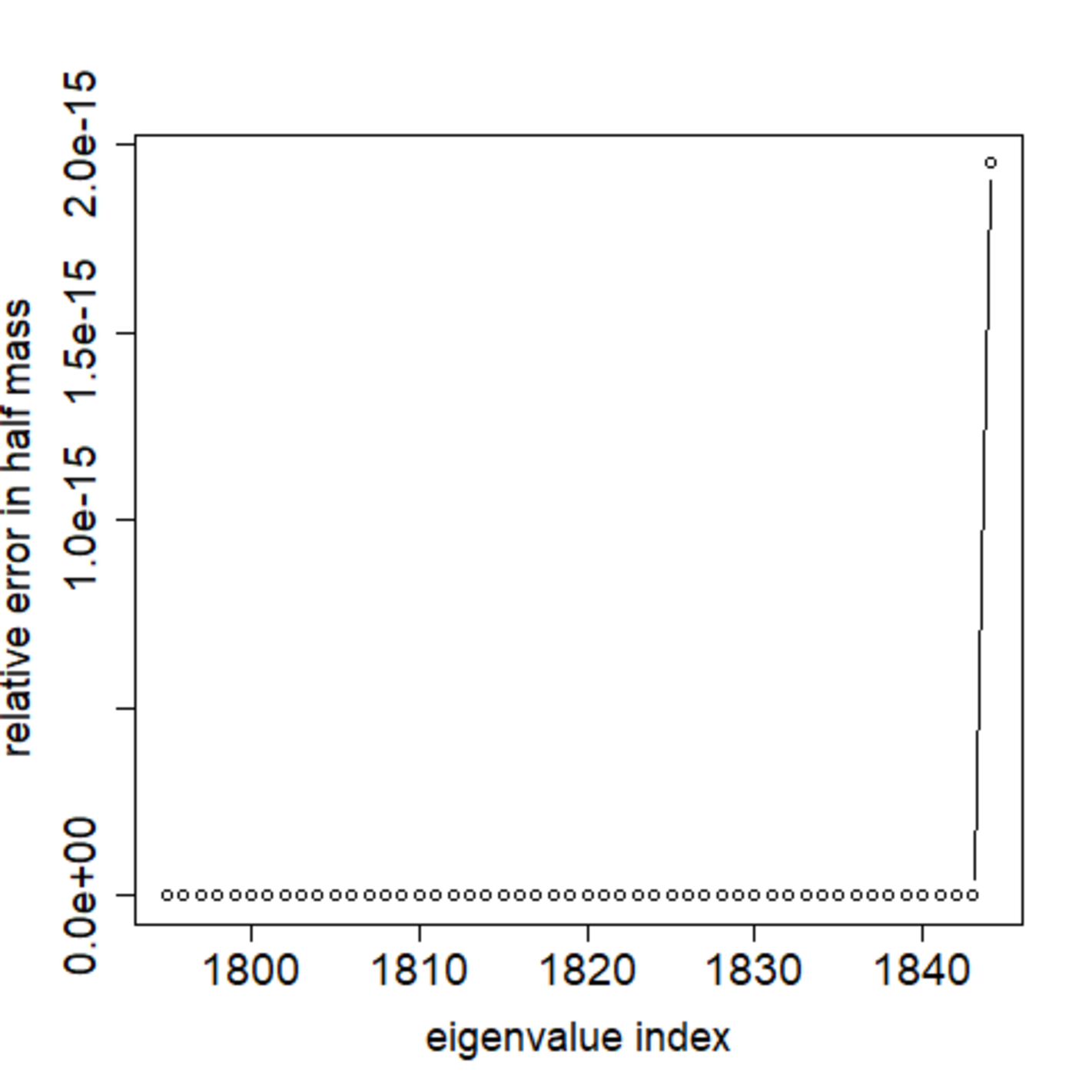

Supplement: S5 File — Names and captions are exactly the same as in S4 File, except that the extension is now TIFF. (ZIP) [file pone.0283413.s005.zip › S4_5_FigBottom.tiff]

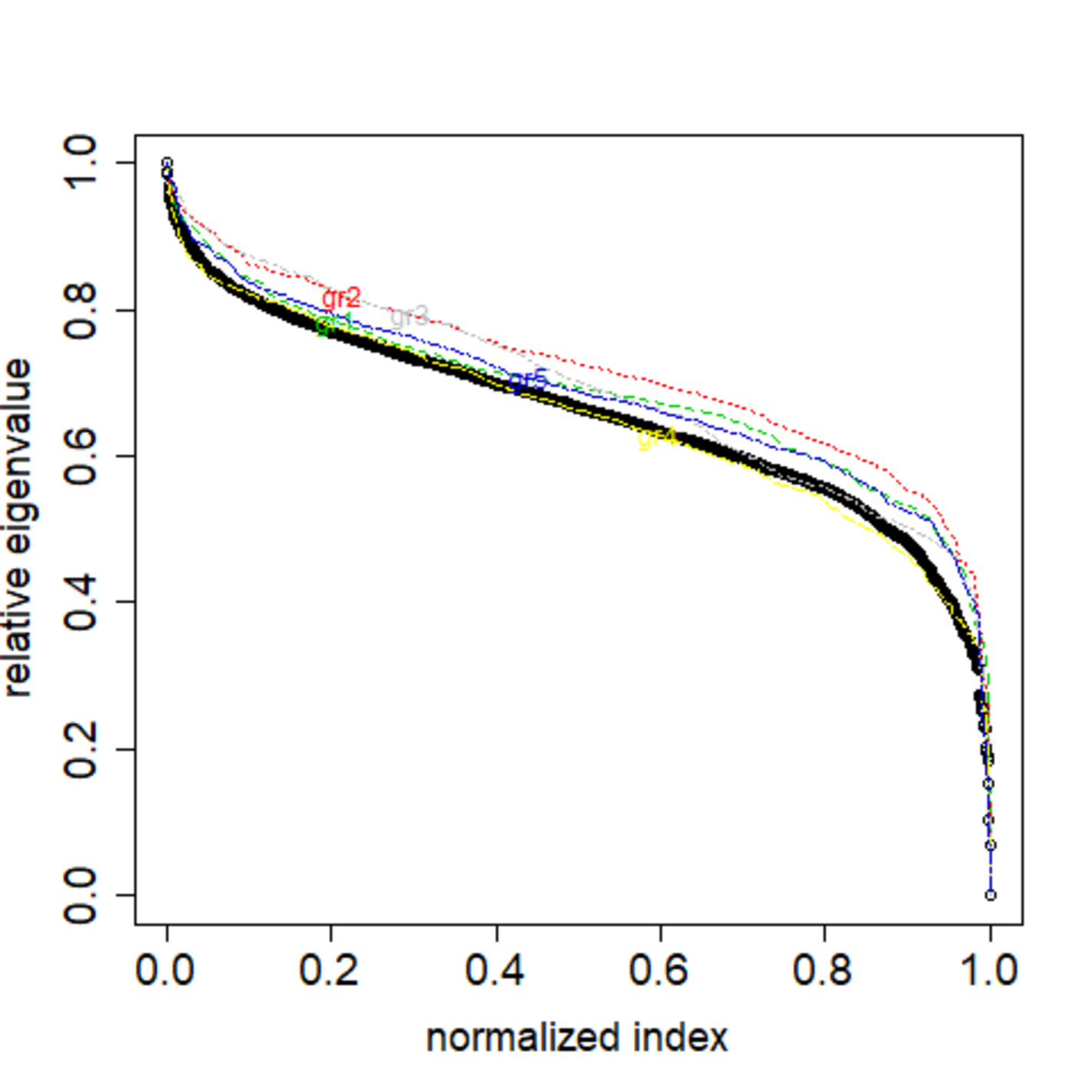

Supplement: S5 File — Names and captions are exactly the same as in S4 File, except that the extension is now TIFF. (ZIP) [file pone.0283413.s005.zip › S4_6_Fig.tiff]

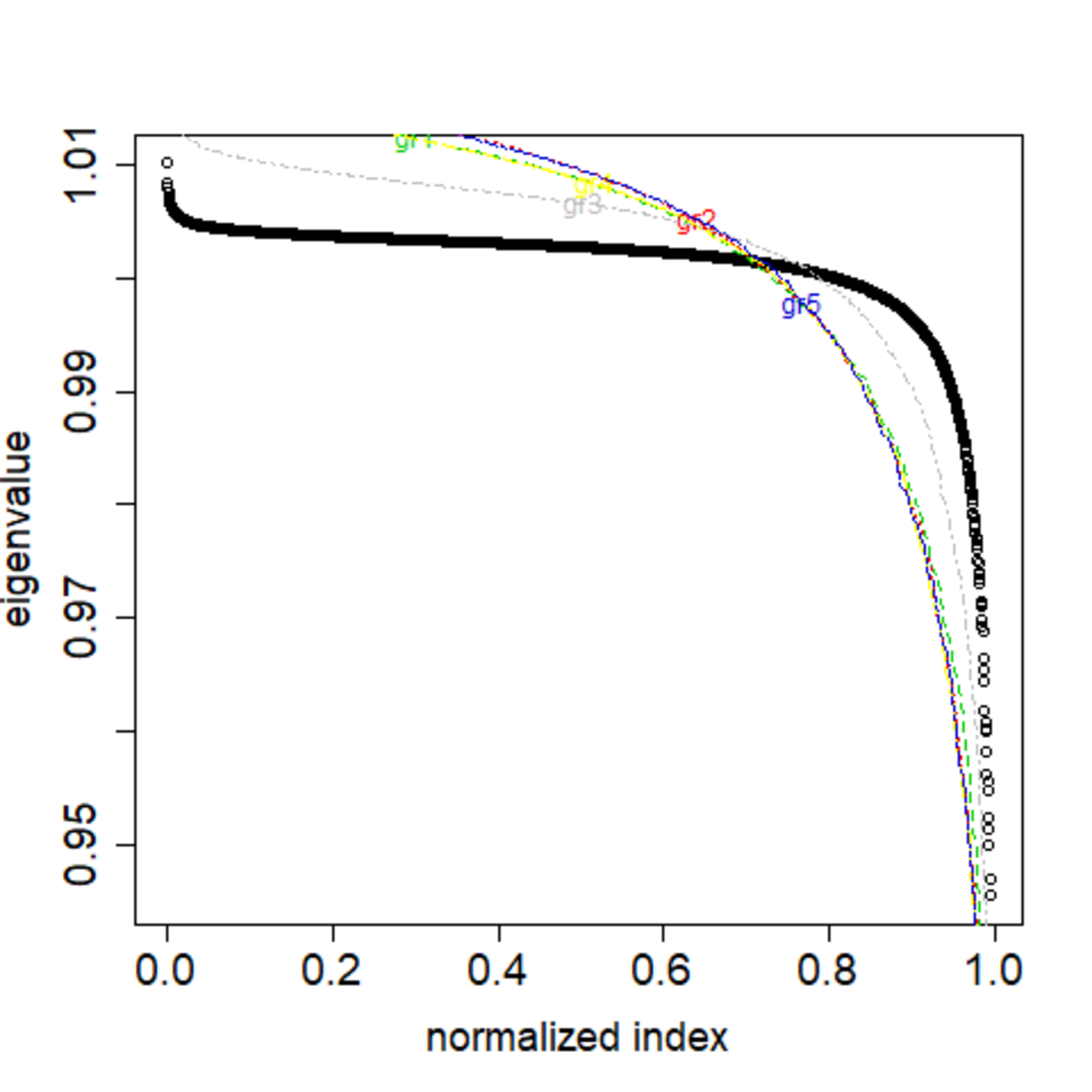

Supplement: S5 File — Names and captions are exactly the same as in S4 File, except that the extension is now TIFF. (ZIP) [file pone.0283413.s005.zip › S4_6_FigBottom.tiff]

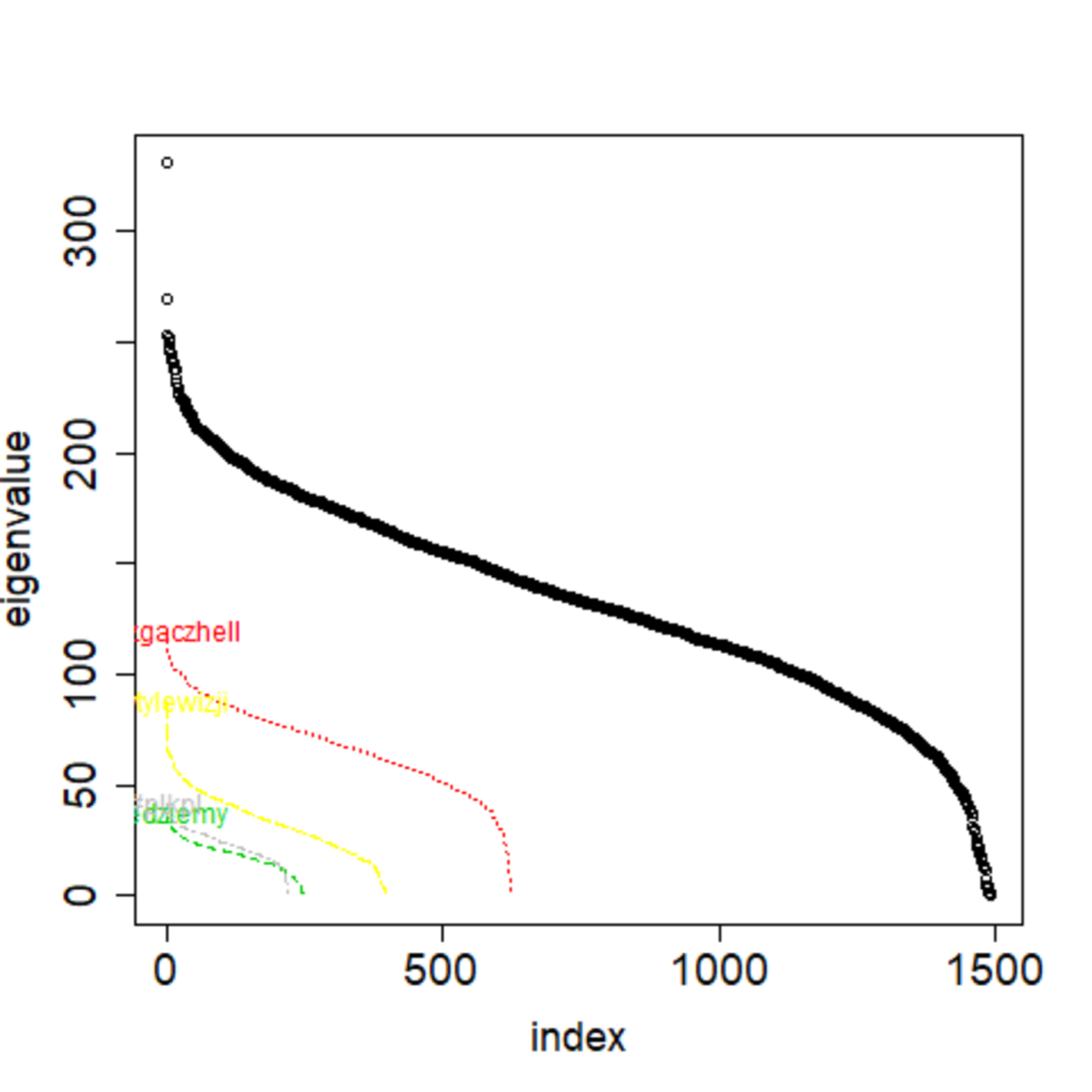

Supplement: S5 File — Names and captions are exactly the same as in S4 File, except that the extension is now TIFF. (ZIP) [file pone.0283413.s005.zip › S4_7_Fig.tiff]

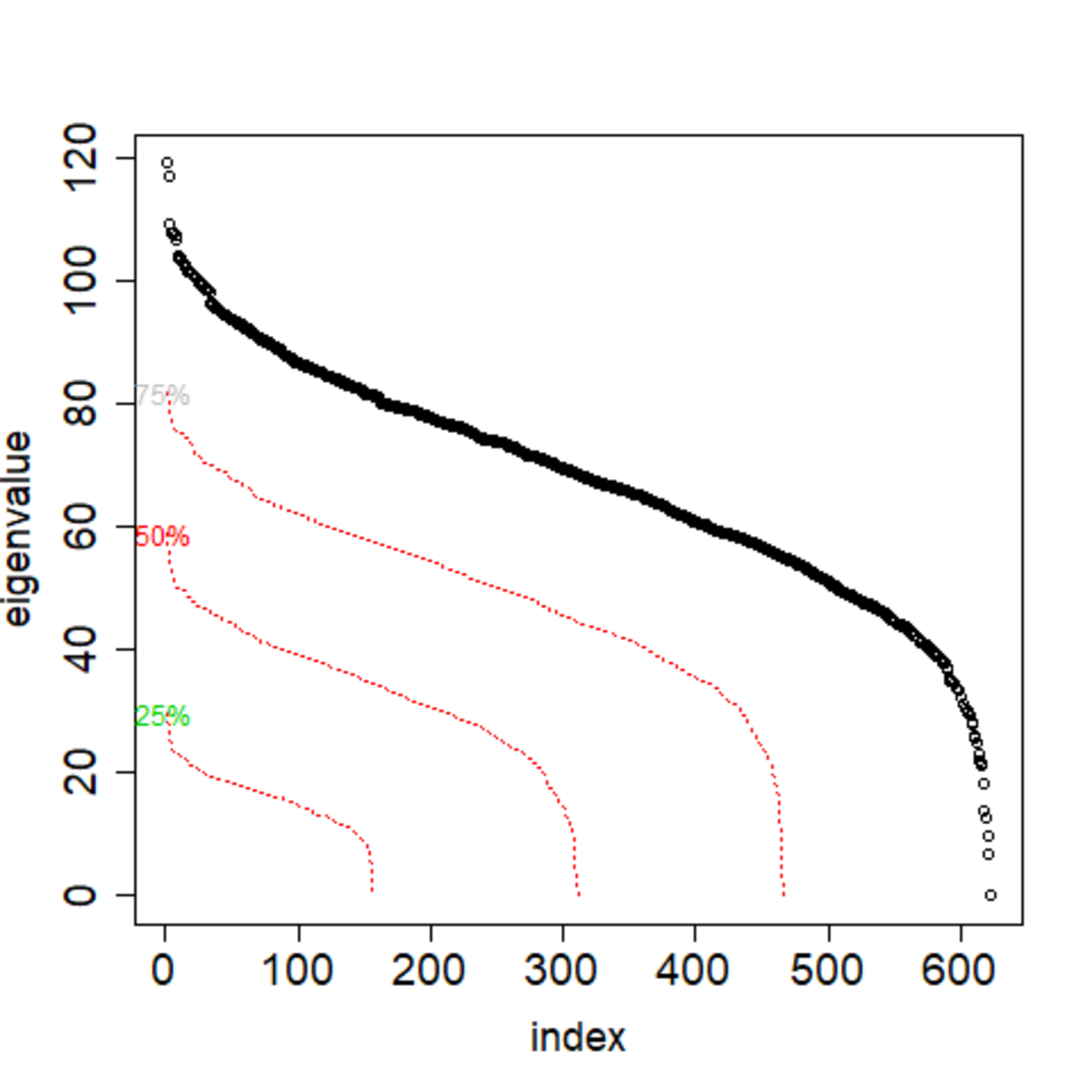

Supplement: S5 File — Names and captions are exactly the same as in S4 File, except that the extension is now TIFF. (ZIP) [file pone.0283413.s005.zip › S4_8_Fig.tiff]

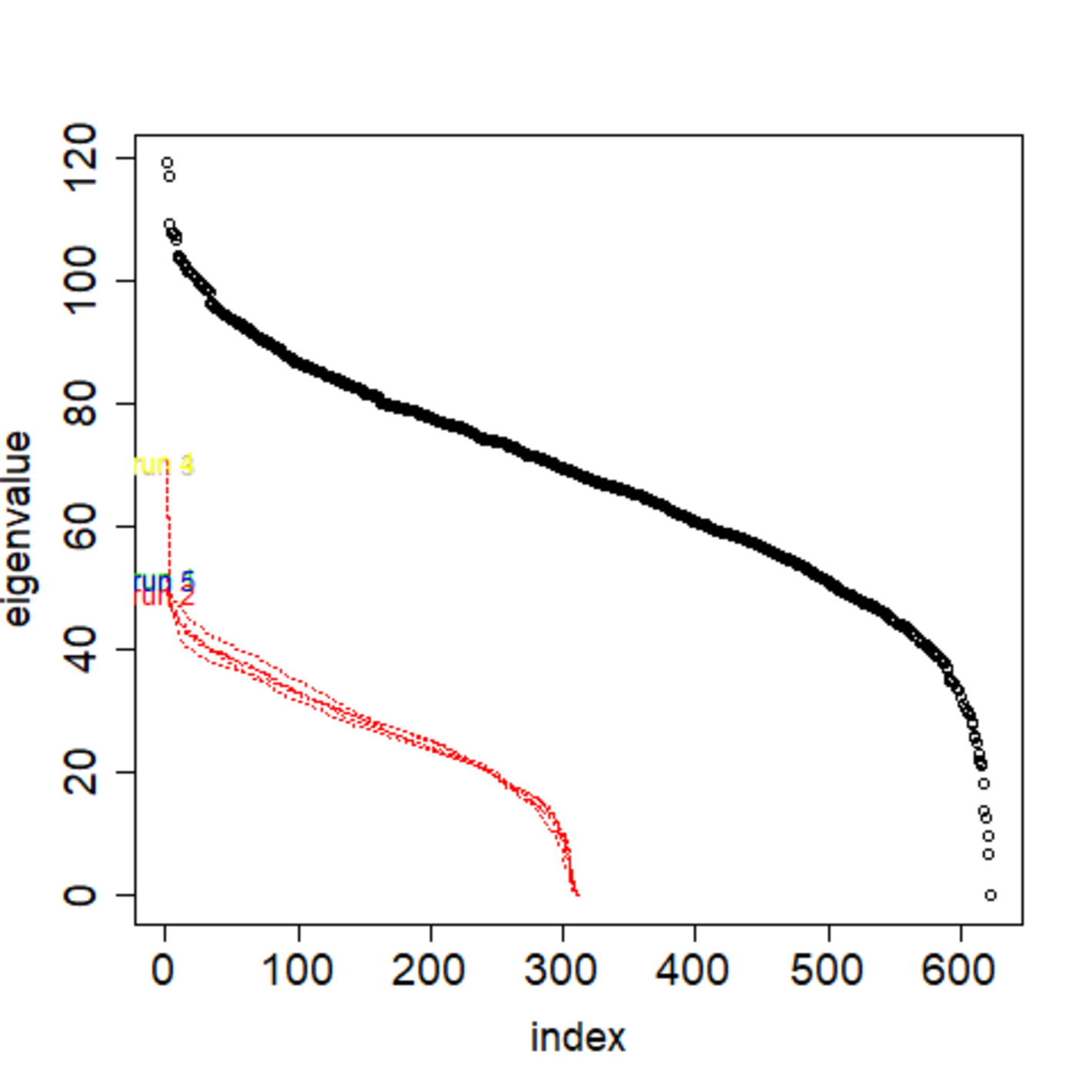

Supplement: S5 File — Names and captions are exactly the same as in S4 File, except that the extension is now TIFF. (ZIP) [file pone.0283413.s005.zip › S4_9_Fig.tiff]

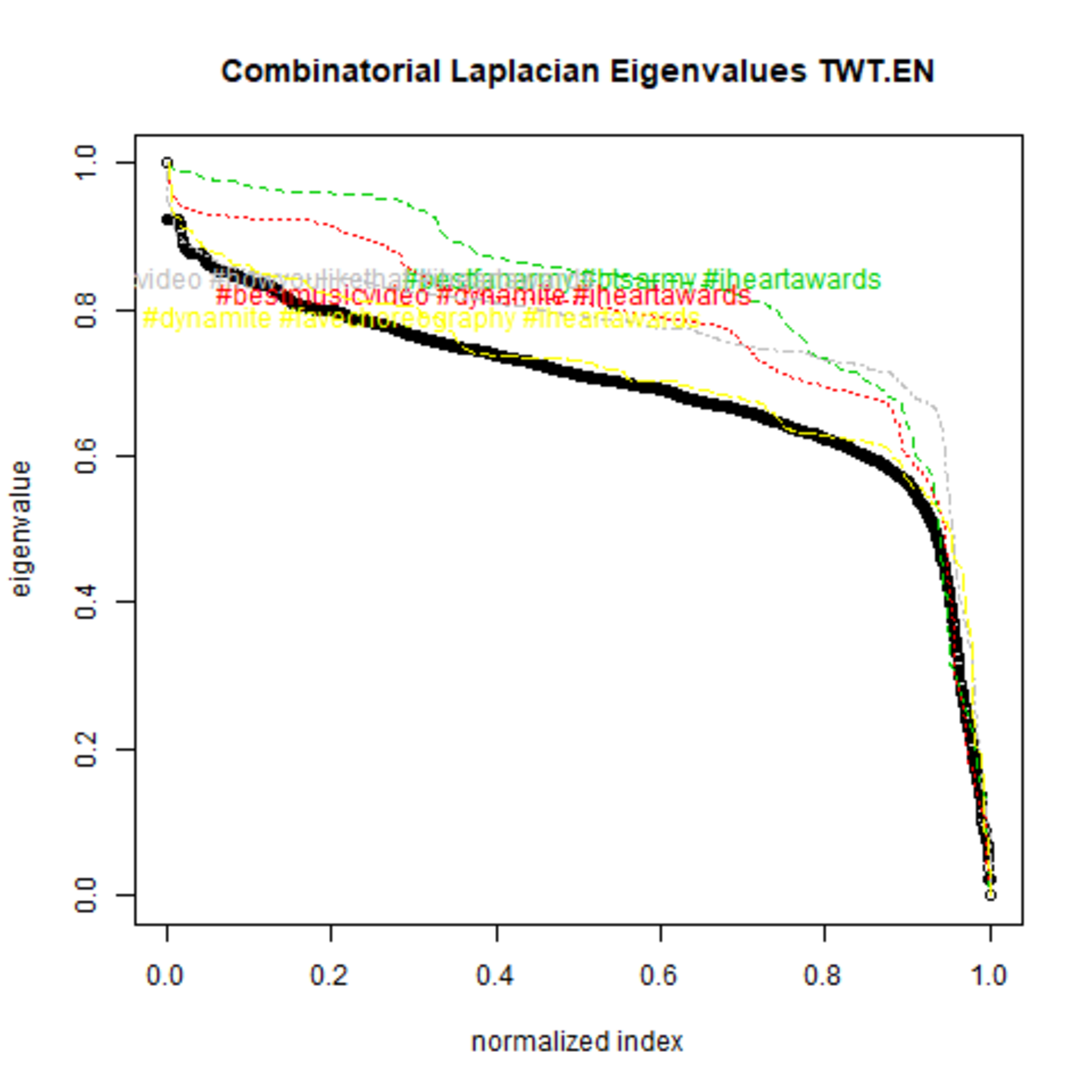

Supplement: S5 File — Names and captions are exactly the same as in S4 File, except that the extension is now TIFF. (ZIP) [file pone.0283413.s005.zip › TWT.ENclassesComb.tiff]
