# Supplementary material for: Eigenvalue based spectral classification
Source: PLoS One. 2023 Apr 6;18(4):e0283413. doi: 10.1371/journal.pone.0283413 (PMC10079090; doi:10.1371/journal.pone.0283413)
Supplement: S1 Appendix — (PDF) [file pone.0283413.s006.pdf]

Appendix

A The generator for BLK.4_0.2_0.5

The BLK generator has been implemented to create synthetic datasets that can be subject to evaluation via the algorithms studied in this paper. It is driven by the following parameters:

- group_count (set to 4) – the number of groups/classes to which the generated documents belong
- ext (set to 2)
- noDocs (set to 2000) – the number of documents that will be generated
- overlap (set to 0.20) – the extent to which the vocabulary of distinct classes shall overlap
- minprob (set to 0.5)

Meth./set	ANO.8	X2	X4	X8
csc.b	44.51	32.14	19.51	4.4
csc.ur	21.98	20.33	6.04	1.37
csc.urdp	23.08	20.33	2.47	1.65
csc.ka	21.98	20.33	2.2	1.1
csc.kadp	23.08	20.33	2.47	1.65
nsc.b	36.81	3.02	3.02	6.59
nsc.ur	7.14	3.02	4.95	3.85
nsc.urdp	6.87	3.02	6.04	3.57
nsc.ursvd	NA	NA	NA	NA
nsc.ursvddp	NA	NA	NA	NA

Table 20. Error percentage for cluster-based classification for the set ANO.8. Columns: 1,2,4,8 times increased number of clusters, row names: GSC methods considered

- noiseprob (set to 10.01)

The name of the generated dataset consists of the components: "BLK.",group_count,"_",overlap,"_",minprob.

It is assumed that the vocabulary used is twice as large as the number of documents. Furthermore, it is assumed that each group uses a separate basic vocabulary (of cardinality gnw plus an overlap with the preceding group), subject to potential noise and overlaps with other groups. Each document contains the same basic number of words dnw (1/30th of gnw times minprob). dnw samples from dnw normal distributions (one from each) are taken to point the position in the dictionary from which the word is to be taken. The standard deviations are the same (1/12th of the group dictionary size), while the means are separated by the group dictionary size divided by the group id plus ext. In this way a kind of different literary styles are simulated: each group has a different number of words at which it is focusing (the first: ext, the second ext+1 etc.). The idea of different literary styles was drawn from observation that different groups of people discuss different number of topics.

After this basic process of generating documents noise is added. The number of noisy points equals noiseprob times number of documents (hence noiseprob is not really a probability, but rather a factor). A noisy point is added by picking two documents and a word from the entire vocabulary. Then with probability of minprob a word is inserted into each of them (the probability is applied separately to both, so that a word is inserted in both at the same time with probability $minprob^2$).

The generator tries to assign nearly the same number of documents to each group. R code is available in Supporting Information File `S3.zip`

B Results of cluster based classification experiments

The detailed description of the contents of the tables can be found in Section 4.3.

C Derivation of formula 3

Let the similarity matrix B of $X_1 \cup X_2 \times X_1 \cup X_2$ would have the form:

$$B = \begin{bmatrix} S & S \\ S & S \end{bmatrix}$$

Meth./set	ANO.8	X2	X4	X8
csc.b	29.57	59.12	73.33	95.59
csc.ur	69.87	72.52	93.98	98.59
csc.urdp	68.55	72.52	97.49	98.3
csc.ka	69.87	72.52	97.8	98.84
csc.kadp	68.55	72.52	97.56	98.3
nsc.b	51.28	97.22	96.63	92.95
nsc.ur	92.37	97.11	94.72	95.21
nsc.urdp	92.64	97.11	93.73	95.73
nsc.ursvd	NA	NA	NA	NA
nsc.ursvddp	NA	NA	NA	NA

Table 21. F1 score for cluster-based classification for the set ANO.8. Columns: 1,2,4,8 times increased number of clusters, row names: GSC methods considered

Meth./set	ANO.26	X2	X4	X8
csc.b	78.1	73.74	52.29	40.56
csc.ur	65.59	48.72	32.51	17.88
csc.urdp	66.26	44.58	31.4	17.54
csc.ka	64.92	45.81	30.17	17.54
csc.kadp	65.81	39.89	31.28	17.32
nsc.b	36.76	22.12	15.98	17.32
nsc.ur	31.06	15.08	20	19.11
nsc.urdp	30.84	14.19	21.45	21.56
nsc.ursvd	NA	NA	NA	NA
nsc.ursvddp	NA	NA	NA	NA

Table 22. Error percentage for cluster-based classification for the set ANO.26. Columns: 1,2,4,8 times increased number of clusters, row names: GSC methods considered

Meth./set	ANO.26	X2	X4	X8
csc.b	8.64	15.02	37.6	52.08
csc.ur	19.04	41.81	58.03	77.51
csc.urdp	18.28	46.4	58.33	74.19
csc.ka	20.62	45.94	60.75	77.95
csc.kadp	18.59	49.4	57.97	79.41
nsc.b	46.3	69.46	80.79	80.36
nsc.ur	51.31	80.11	76.76	75.67
nsc.urdp	52.09	81.21	75.01	76.15
nsc.ursvd	NA	NA	NA	NA
nsc.ursvddp	NA	NA	NA	NA

Table 23. F1 score for cluster-based classification for the set ANO.26. Columns: 1,2,4,8 times increased number of clusters, row names: GSC methods considered

Meth./set	ANO.44	X2	X4	X8
csc.b	21.23	0	4.11	4.11
csc.ur	20.55	0	0.68	3.42
csc.urdp	1.37	0	1.37	4.11
csc.ka	20.55	0	0.68	3.42
csc.kadp	1.37	0	1.37	4.11
nsc.b	1.37	0.68	2.74	3.42
nsc.ur	0.68	3.42	2.74	3.42
nsc.urdp	0	7.53	3.42	3.42
nsc.ursvd	0.68	3.42	2.05	3.42
nsc.ursvddp	0	7.53	3.42	3.42

Table 24. Error percentage for cluster-based classification for the set ANO.44. Columns: 1,2,4,8 times increased number of clusters, row names: GSC methods considered

Meth./set	ANO.44	X2	X4	X8
csc.b	70.03	100	95.5	95.54
csc.ur	71.52	100	99.25	96.32
csc.urdp	98.55	100	98.51	95.72
csc.ka	71.52	100	99.25	96.32
csc.kadp	98.55	100	98.51	95.72
nsc.b	98.61	99.31	97.01	96.52
nsc.ur	99.31	96.55	97.01	96.52
nsc.urdp	100	92.15	96.27	96.52
nsc.ursvd	99.31	96.55	97.75	96.52
nsc.ursvddp	100	92.15	96.27	96.52

Table 25. F1 score for cluster-based classification for the set ANO.44. Columns: 1,2,4,8 times increased number of clusters, row names: GSC methods considered

Meth./set	ANO.94	X2	X4	X8
csc.b	63.37	63.26	63	35.3
csc.ur	63.37	35.73	34.02	18.61
csc.urdp	63.37	35.67	14.14	15.52
csc.ka	63.37	35.73	34.02	22.65
csc.kadp	63.37	35.73	14.14	14.89
nsc.b	49.71	6.91	18.61	17.86
nsc.ur	63.32	3.46	3.67	17.7
nsc.urdp	63.37	4.68	10.15	16.11
nsc.ursvd	35.19	4.36	8.98	18.71
nsc.ursvddp	5.42	7.71	9.84	21.85

Table 26. Error percentage for cluster-based classification for the set ANO.94. Columns: 1,2,4,8 times increased number of clusters, row names: GSC methods considered

Meth./set	ANO.94	X2	X4	X8
csc.b	18.14	18.38	18.93	56.27
csc.ur	18.15	55.32	58.93	81.69
csc.urdp	18.15	55.35	86.25	85.04
csc.ka	18.15	55.32	58.93	76.91
csc.kadp	18.15	55.32	86.25	85.66
nsc.b	38.8	93.41	79.65	82.52
nsc.ur	18.27	96.7	96.45	82.78
nsc.urdp	18.15	95.49	90.36	84.43
nsc.ursvd	55.52	95.83	91.36	82.13
nsc.ursvddp	94.84	92.71	90.5	78.82

Table 27. F1 score for cluster-based classification for the set ANO.94. Columns: 1,2,4,8 times increased number of clusters, row names: GSC methods considered

Meth./set	SEN.EN.maj	X2	X4	X8
csc.b	31.56	31.56	31.38	31.38
csc.ur	31.56	31.56	31.38	31.21
csc.urdp	31.56	31.56	31.38	31.03
csc.ka	31.56	31.56	31.38	31.21
csc.kadp	31.56	31.56	31.38	31.21
nsc.b	31.56	29.08	29.43	28.55
nsc.ur	31.56	29.43	30.32	30.32
nsc.urdp	31.56	29.96	29.96	30.14
nsc.ursvd	31.56	29.43	30.32	30.5
nsc.ursvddp	31.56	29.96	29.96	30.14

Table 28. Error percentage for cluster-based classification for the set SEN.EN.maj. Columns: 1,2,4,8 times increased number of clusters, row names: GSC methods considered

Meth./set	SEN.EN.maj	X2	X4	X8
csc.b	40.63	40.63	41.24	41.24
csc.ur	40.63	40.63	41.76	42.85
csc.urdp	40.63	40.63	41.76	43.42
csc.ka	40.63	40.63	41.76	42.85
csc.kadp	40.63	40.63	41.76	42.85
nsc.b	40.63	57.86	60.19	59.66
nsc.ur	40.63	58.06	52.84	57.83
nsc.urdp	40.63	55.68	53.08	57.96
nsc.ursvd	40.63	58.06	52.84	53.33
nsc.ursvddp	40.63	55.68	53.08	57.96

Table 29. F1 score for cluster-based classification for the set SEN.EN.maj. Columns: 1,2,4,8 times increased number of clusters, row names: GSC methods considered

Meth./set	SEN.EN.ent	X2	X4	X8
csc.b	24.32	24.22	24.22	23.92
csc.ur	24.32	24.32	24.42	20.92
csc.urdp	24.42	24.32	24.42	20.32
csc.ka	24.32	24.32	24.42	21.42
csc.kadp	24.42	24.32	24.42	20.72
nsc.b	24.42	20.12	19.22	19.42
nsc.ur	24.42	21.82	20.32	19.32
nsc.urdp	21.92	24.42	20.62	18.62
nsc.ursvd	24.42	21.82	20.32	19.32
nsc.ursvddp	21.92	24.42	20.62	19.32

Table 30. Error percentage for cluster-based classification for the set SEN.EN.ent. Columns: 1,2,4,8 times increased number of clusters, row names: GSC methods considered

Meth./set	SEN.EN.ent	X2	X4	X8
csc.b	29.23	29.76	29.76	31.49
csc.ur	29.23	29.73	30.32	48.79
csc.urdp	29.2	29.72	28.7	49.27
csc.ka	29.23	29.73	30.32	47.89
csc.kadp	29.2	29.72	29.25	48.82
nsc.b	28.7	49.44	58.6	59.72
nsc.ur	28.7	48.48	54.66	60.41
nsc.urdp	48.6	28.7	47.17	59.21
nsc.ursvd	28.7	48.48	54.66	60.41
nsc.ursvddp	48.6	28.7	47.17	50.39

Table 31. F1 score for cluster-based classification for the set SEN.EN.ent. Columns: 1,2,4,8 times increased number of clusters, row names: GSC methods considered

Meth./set	SEN.PL.maj	X2	X4	X8
csc.b	0	44.27	44.1	43.42
csc.ur	0	43.42	43.59	34.87
csc.urdp	44.44	44.44	42.39	31.97
csc.ka	44.44	43.42	43.59	33.68
csc.kadp	44.44	44.44	42.39	37.09
nsc.b	0	33.68	33.33	28.72
nsc.ur	0	37.09	32.31	29.91
nsc.urdp	34.19	31.28	31.97	28.55
nsc.ursvd	34.19	31.28	31.97	28.55
nsc.ursvddp	33.5	35.9	31.62	28.72

Table 32. Error percentage for cluster-based classification for the set SEN.PL.maj. Columns: 1,2,4,8 times increased number of clusters, row names: GSC methods considered

Meth./set	SEN.PL.maj	X2	X4	X8
csc.b	35.72	36.14	36.56	38.21
csc.ur	35.72	39.78	38.12	64.94
csc.urdp	35.72	35.72	50.24	64.26
csc.ka	35.72	39.78	38.12	66.14
csc.kadp	35.72	35.72	50.24	62.91
nsc.b	35.72	59.91	59.92	71.16
nsc.ur	35.72	60.98	62.59	69.3
nsc.urdp	63.23	65.44	62.98	70.73
nsc.ursvd	64.12	65.44	62.98	70.73
nsc.ursvddp	64.37	61.16	63.06	70.83

Table 33. F1 score for cluster-based classification for the set SEN.PL.maj. Columns: 1,2,4,8 times increased number of clusters, row names: GSC methods considered

Meth./set	SEN.PL.ent	X2	X4	X8
csc.b	57.31	57.08	56.39	55.35
csc.ur	57.42	57.54	55.93	47.3
csc.urdp	57.54	57.54	55.7	45.57
csc.ka	57.42	57.54	55.93	50.06
csc.kadp	57.54	57.54	55.81	49.25
nsc.b	49.83	44.99	42.69	40.74
nsc.ur	48.33	47.3	42.58	39.36
nsc.urdp	47.18	48.1	42.69	40.62
nsc.ursvd	48.33	47.3	42.58	39.47
nsc.ursvddp	47.18	47.99	42.69	40.16

Table 34. Error percentage for cluster-based classification for the set SEN.PL.ent. Columns: 1,2,4,8 times increased number of clusters, row names: GSC methods considered

Meth./set	SEN.PL.ent	X2	X4	X8
csc.b	12.72	13.32	15.62	18.64
csc.ur	12.32	13.59	19.37	42.99
csc.urdp	11.92	13.59	18.38	36.01
csc.ka	12.32	13.59	18.64	41.53
csc.kadp	11.92	13.59	19.5	40.8
nsc.b	23.19	38.02	43.09	49.88
nsc.ur	24.41	33.2	41.94	52.4
nsc.urdp	33.45	36.95	42.11	50.33
nsc.ursvd	24.41	33.2	41.94	51.26
nsc.ursvddp	33.45	37.03	41.72	51.34

Table 35. F1 score for cluster-based classification for the set SEN.PL.ent. Columns: 1,2,4,8 times increased number of clusters, row names: GSC methods considered

Meth./set	TWT.EN	X2	X4	X8
csc.b	75.16	74.89	74.35	73.32
csc.ur	61.5	61.33	63.61	52.77
csc.urdp	67.19	63.67	60.74	54.01
csc.ka	61.5	61.39	63.56	54.77
csc.kadp	67.14	64.75	55.91	52.77
nsc.b	51.25	51.03	53.47	47.83
nsc.ur	49.78	55.97	52.28	46.26
nsc.urdp	50.38	55.37	53.69	48.86
nsc.ursvd	49.78	52.6	53.58	47.61
nsc.ursvddp	50.38	55.1	53.15	46.75

Table 36. Error percentage for cluster-based classification for the set TWT.EN. Columns: 1,2,4,8 times increased number of clusters, row names: GSC methods considered

Meth./set	TWT.EN	X2	X4	X8
csc.b	8.37	8.94	9.94	11.75
csc.ur	28.02	33.48	26.09	40.54
csc.urdp	18.13	20.28	27.18	33.51
csc.ka	28.02	33.39	26.14	39.51
csc.kadp	18.16	19.69	30.56	34.96
nsc.b	44	40.95	43.01	48.04
nsc.ur	45.2	40.67	43.48	50.04
nsc.urdp	44.77	36.72	42.04	49.08
nsc.ursvd	45.2	41.49	40.31	48.53
nsc.ursvddp	44.77	37.14	41.09	51.39

Table 37. F1 score for cluster-based classification for the set TWT.EN. Columns: 1,2,4,8 times increased number of clusters, row names: GSC methods considered

Meth./set	TWT.PL	X2	X4	X8
csc.b	58.08	57.81	57.41	56.54
csc.ur	58.15	57.01	57.34	53.39
csc.urdp	58.01	57.75	56.94	53.92
csc.ka	58.15	57.41	57.28	54.59
csc.kadp	58.01	57.75	56.94	53.66
nsc.b	58.28	57.95	54.73	51.58
nsc.ur	58.28	58.08	55.8	51.37
nsc.urdp	58.28	58.22	54.33	51.31
nsc.ursvd	58.28	58.28	55.47	51.44
nsc.ursvddp	58.28	58.22	53.59	51.11

Table 38. Error percentage for cluster-based classification for the set TWT.PL. Columns: 1,2,4,8 times increased number of clusters, row names: GSC methods considered

Meth./set	TWT.PL	X2	X4	X8
csc.b	15.29	15.96	16.91	19.01
csc.ur	15.06	19.04	20	27.37
csc.urdp	15.4	16.11	22.53	27.57
csc.ka	15.06	18.24	19.8	27.1
csc.kadp	16.58	16.11	22.53	28.83
nsc.b	14.72	18.72	25.94	32.31
nsc.ur	14.72	18.74	26.24	36.38
nsc.urdp	14.72	18.41	29.87	34.97
nsc.ursvd	14.72	14.72	24.02	36.29
nsc.ursvddp	14.72	18.41	30.28	37.08

Table 39. F1 score for cluster-based classification for the set TWT.PL. Columns: 1,2,4,8 times increased number of clusters, row names: GSC methods considered

Meth./set	BLK.4.0.2.0.5	X2	X4	X8
csc.b	34.4	20.05	14.9	14.9
csc.ur	24.75	14.85	8.2	3.45
csc.urdp	25	14.85	5	5.05
csc.ka	24.75	14.85	9.9	3.2
csc.kadp	25	14.85	9.95	5.05
nsc.b	25.05	24.2	23.3	18.65
nsc.ur	25.05	20.05	19.5	14.3
nsc.urdp	25.3	19.9	18.85	12.95
nsc.ursvd	25.05	20.05	19.85	14.15
nsc.ursvddp	25.3	19.9	17.7	14.25

Table 40. Error percentage for cluster-based classification for the set BLK.4-0.2-0.5. Columns: 1,2,4,8 times increased number of clusters, row names: GSC methods considered

Meth./set	BLK.4.0.2.0.5	X2	X4	X8
csc.b	57.54	80.72	85.38	85.38
csc.ur	74.65	85.1	91.54	96.55
csc.urdp	74.4	85.1	94.95	94.9
csc.ka	74.65	85.1	90.05	96.8
csc.kadp	74.4	85.1	90	94.9
nsc.b	75.34	76.1	76.98	82.28
nsc.ur	75.34	79.43	80.14	85.62
nsc.urdp	75.07	79.53	80.57	87.05
nsc.ursvd	75.34	79.43	79.81	85.77
nsc.ursvddp	75.07	79.53	81.83	85.69

Table 41. F1 score for cluster-based classification for the set BLK.4-0.2-0.5. Columns: 1,2,4,8 times increased number of clusters, row names: GSC methods considered

Let $L(S)$ be the combinatorial Laplacian of S. Note that accidentally even if $S[j,j]=1$, $L(S)$ would be the same as in case of $S[j,j]$ would have been set to zero or any other number (this does not hold for normalized Laplacians). Let λ be an eigenvalue associated with the eigenvector $\mathbf{v}$ of $L(S)$. Let $d(S)$ be the diagonal of matrix of S, and $D(S)$ be the diagonal matrix where each diagonal element corresponds to column sum of S. With this notation: $L(S)=D(S)-S=D(S-d(S))-(S-d(S))$.

So

$$\begin{aligned} L(B) = D(B) - B &= D\left(\begin{bmatrix} S & S \\ S & S \end{bmatrix}\right) - \begin{bmatrix} S & S \\ S & S \end{bmatrix} \\ &= \begin{bmatrix} 2D(S) & \mathbf{0} \\ \mathbf{0} & 2D(S) \end{bmatrix} - \begin{bmatrix} S & S \\ S & S \end{bmatrix} \\ &= \begin{bmatrix} L(S)+D(S) & -S \\ -S & L(S)+D(S) \end{bmatrix} \end{aligned}$$

If now $(\lambda, \mathbf{v})$ is the eigenpair of the Laplacian $L(S)$, then we get

$$L(B)\begin{bmatrix} \mathbf{v} \\ \mathbf{v} \end{bmatrix} = \begin{bmatrix} L(S)\mathbf{v} + \big(D(S)-S\big)\mathbf{v} \\ \big(D(S)-S\big)\mathbf{v} + L(S)\mathbf{v} \end{bmatrix} = 2\lambda \begin{bmatrix} \mathbf{v} \\ \mathbf{v} \end{bmatrix}$$

which means that 2λ is the eigenvalue of $L(B)$ and $(\mathbf{v}^T, \mathbf{v}^T)^T$ is its eigenvector. It turns out that for twice as big "exact" samples from some document set with a well defined "style", or "theme", or "topic", as used in PLSA or LDA document analysis, have twice as big eigenvalues. Same can be repeated for splitting the dataset into more equally sized subsets. This fact justifies the usage of sample size normalization which we apply in our algorithm. It easily seen that if S is replicated in the matrix B not twice but n times in a row, then $n\lambda$ is the eigenvalue of $L(B)$.

D A note on the issue of similarity of eigenvalue distributions of normalized Laplacian of a document class and its subsets

Let us consider also briefly the normalized Laplacian, as defined by Eq (2), that is $\mathcal{L} = D^{-1/2} L D^{-1/2} = I - D^{-1/2} S D^{-1/2}$. A general assumption is the literature is that the diagonal of S must be 0. But we assume to the contrary that it is the typical similarity value. With the notation from section 7.2:
$\mathcal{L}(S) = D(S)^{-1/2}(D(S)-S)D(S)^{-1/2}$.

So

$$\begin{aligned}
\mathcal{L}(B) &= D^{-1/2}(B)(D(B)-B)D^{-1/2}(B) = \\
&= D^{-1/2}(B)\left(D\left(\begin{bmatrix} S & S \\ S & S \end{bmatrix}\right) - \begin{bmatrix} S & S \\ S & S \end{bmatrix}\right) D^{-1/2}(B) \\
&= \begin{bmatrix} \frac{D^{-\frac{1}{2}}(S)}{\sqrt{2}} & \mathbf{0} \\ \mathbf{0} & \frac{D^{-\frac{1}{2}}(S)}{\sqrt{2}} \end{bmatrix} \begin{bmatrix} 2D(S)-S & -S \\ -S & 2D(S)-S \end{bmatrix} \begin{bmatrix} \frac{D^{-\frac{1}{2}}(S)}{\sqrt{2}} & \mathbf{0} \\ \mathbf{0} & \frac{D^{-\frac{1}{2}}(S)}{\sqrt{2}} \end{bmatrix} \\
&= \begin{bmatrix} \sqrt{2}D^{\frac{1}{2}}(S) - \frac{D^{-\frac{1}{2}}(S)}{\sqrt{2}}S & -\frac{D^{-\frac{1}{2}}(S)}{\sqrt{2}}S \\ -\frac{D^{-\frac{1}{2}}(S)}{\sqrt{2}}S & \sqrt{2}D^{\frac{1}{2}}(S) - \frac{D^{-\frac{1}{2}}(S)}{\sqrt{2}}S \end{bmatrix} \begin{bmatrix} \frac{D^{-\frac{1}{2}}(S)}{\sqrt{2}} & \mathbf{0} \\ \mathbf{0} & \frac{D^{-\frac{1}{2}}(S)}{\sqrt{2}} \end{bmatrix} \\
&= \begin{bmatrix} I - \frac{1}{2}D^{-1/2}(S)SD^{-1/2}(S) & -\frac{1}{2}D^{-1/2}(S)SD^{-1/2}(S) \\ -\frac{1}{2}D^{-1/2}(S)SD^{-1/2}(S) & I - \frac{1}{2}D^{-1/2}(S)SD^{-1/2}(S) \end{bmatrix} \\
&= \begin{bmatrix} \frac{1}{2}\mathcal{L}(S) + \frac{1}{2}I & \frac{1}{2}\mathcal{L}(S) - \frac{1}{2}I \\ \frac{1}{2}\mathcal{L}(S) - \frac{1}{2}I & \frac{1}{2}\mathcal{L}(S) + \frac{1}{2}I \end{bmatrix} = \frac{1}{2}\begin{bmatrix} \mathcal{L}(S)+I & \mathcal{L}(S)-I \\ \mathcal{L}(S)-I & \mathcal{L}(S)+I \end{bmatrix}
\end{aligned}$$

If now $(\lambda, \mathbf{v})$ is the eigenpair of the Laplacian $L(S)$, then we get

$$\begin{aligned}
\mathcal{L}(B)[\mathbf{v}^T\mathbf{v}^T]^T &= \\
&= \frac{1}{2}\begin{bmatrix} \mathcal{L}(S)+I & \mathcal{L}(S)-I \\ \mathcal{L}(S)-I & \mathcal{L}(S)+I \end{bmatrix}\begin{bmatrix} \mathbf{v} \\ \mathbf{v} \end{bmatrix} \\
&= \frac{1}{2}\begin{bmatrix} 2\mathcal{L}(S)\mathbf{v} \\ 2\mathcal{L}(S)\mathbf{v} \end{bmatrix} \\
&= \lambda[\mathbf{v}^T\mathbf{v}^T]^T
\end{aligned}$$

which implies that there is no need for scaling under normalized Laplacian, λ is eigenvalue of both $\mathcal{L}(B)$ and $\mathcal{L}(S)$. However, as mentioned at the beginning of this section, an inaccurate assumption was made. We need in fact to consider $\mathcal{L}(B - d(B))$ versus $\mathcal{L}(S - d(S))$ Let us denote $B' = B - d(B)$ and $S' = S - d(S)$. Then

So

$$\begin{aligned}
\mathcal{L}(B') &= D^{-\frac{1}{2}}(B')(D(B')-B')D^{-\frac{1}{2}}(B') = \\
&= D^{-\frac{1}{2}}(B')\left(D\left(\begin{bmatrix} S' & S \\ S & S' \end{bmatrix}\right) - \begin{bmatrix} S' & S \\ S & S' \end{bmatrix}\right) D^{-\frac{1}{2}}(B') \\
&= \begin{bmatrix} (2D(S')+d(S))^{-\frac{1}{2}} & \mathbf{0} \\ \mathbf{0} & (2D(S')+d(S))^{-\frac{1}{2}} \end{bmatrix} \\
&\cdot \begin{bmatrix} 2D(S')+d(S)-S' & -S \\ -S & 2D(S')+d(S)-S' \end{bmatrix} \\
&\cdot \begin{bmatrix} (2D(S')+d(S))^{-\frac{1}{2}} & \mathbf{0} \\ \mathbf{0} & (2D(S')+d(S))^{-\frac{1}{2}} \end{bmatrix} \\
&= \begin{bmatrix} I - D_d^{-\frac{1}{2}}S'D_d^{-\frac{1}{2}} & -D_d^{-\frac{1}{2}}SD_d^{-\frac{1}{2}} \\ -D_d^{-\frac{1}{2}}SD_d^{-\frac{1}{2}} & I - D_d^{-\frac{1}{2}}S'D_d^{-\frac{1}{2}} \end{bmatrix}
\end{aligned}$$

where $D_d = (2D(S') + d(S))$ Obviously, there is no way for expressing $\mathcal{L}(B')$ in terms of $\mathcal{L}(S')$ and therefore the classification results will be approximate only. Maybe this insight constitutes a hint that the concept of normalized Laplacian needs to be revisited or at least considered in two versions.
